# Changing trends in the disease burden of uterine cancer globally from 1990 to 2019 and its predicted level in 25 years

**DOI:** 10.3389/fonc.2024.1361419

**Published:** 2024-04-22

**Authors:** Shuang Song, Dandan Zhang, Yizi Wang, Zixuan Song

**Affiliations:** ^1^Department of Obstetrics and Gynecology, Shengjing Hospital of China Medical University, Shenyang, China; ^2^Department of Obstetrics and Gynecology, Hirosaki University Graduate School of Medicine, Hirosaki, Aomori, Japan; ^3^Department of Health Management, Shengjing Hospital of China Medical University, Shenyang, China

**Keywords:** uterine cancer, global, burden, predict, trend

## Abstract

**Background:**

We aim to evaluate the global, regional, and national burden of Uterine Cancer (UC) from 1990 to 2019.

**Methods:**

We gathered UC data across 204 countries and regions for the period 1990-2019, utilizing the Global Burden of Disease Database (GBD) 2019 public dataset. Joinpoint regression analysis was employed to pinpoint the year of the most significant changes in global trends. To project the UC trajectory from 2020 to 2044, we applied the Nordpred analysis, extrapolating based on the average trend observed in the data. Furthermore, the Bayesian Age-Period-Cohort (BAPC) model with integrated nested Laplace approximations was implemented to confirm the stability of the Nordpred analysis predictions.

**Results:**

Globally, the age-standardized rate (ASR) of incidence for UC has increased from 1990 to 2019 with an Average Annual Percentage Change (AAPC) of 0.50%. The ASR for death has declined within the same period (AAPC: -0.8%). An increase in the ASR of incidence was observed across all Socio-demographic Index (SDI) regions, particularly in High SDI regions (AAPC: 1.12%), while the ASR for death decreased in all but the Low SDI regions. Over the past 30 years, the highest incidence rate was observed in individuals aged 55-59 (AAPC: 0.76%). Among 204 countries and regions, there was an increase in the ASR of incidence in 165 countries and an increase in the ASR of deaths in 77 countries. Our projections suggest that both the incidence and death rates for UC are likely to continue their decline from 2020 to 2044.

**Conclusions:**

UC has significantly impacted global health negatively, with its influence stemming from a range of factors including geographical location, age-related and racial disparities, and SDI.

## 1 Introduction

In 2015, the United Nations unveiled the Sustainable Development Goals (SDG). SDG Goal 3.4 aims to reduce the global premature mortality rate from noncommunicable diseases, including cancer, by one-third by 2030 (1). Uterine Cancer (UC) is the sixth most prevalent malignant disorder among females globally. It accounts for approximately 4% of all cancer-related fatalities in women (2). UC is characterized by tumors developing in the upper two-thirds of the uterus, termed the corpus, situated above the uterus’s internal orifice. GLOBOCAN 2020 reported 417,367 new cases of UC globally and 97,370 associated deaths (1). It is important to recognize that the incidence and mortality rates of UC exhibit significant variations across geographical regions. High Socio-demographic Index (SDI) areas generally see increased incidence rates of UC, whereas regions with Low SDI often experience higher mortality rates. Northern America recorded the highest incidence rate at 21.1 age-standardized rate (ASR) per 100,000 population, while Polynesia had the highest death rate, with an ASR of 4.3 per 100,000 population (3). For global cases of UC, the ASR has increased from 8.67 to 9.99 per 100,000 population over the three decades spanning from 1990 to 2019. In contrast, the ASR of mortality has declined from 2.6 to 2.09 per 100,000 population within this same timeframe.

Over the span of three decades, there has been an increase in the incidence ASR across all SDI regions, with a pronounced rise in high SDI regions. In contrast, the ASR of death has diminished in all SDI regions, with the exception of the low SDI regions. The most significant decline was observed in the High-middle and Middle SDI regions. Understanding the geographic and temporal trends of uterine cancer prevalence on a global, regional, and national scale is imperative. Developing informed policies and allocating resources judiciously require an understanding of diverse population challenges. Consequently, we employed Global Burden of Disease Database (GBD) to assess the incidence, prevalence, mortality, disability-adjusted life-years (DALYs), years lived with disability (YLDs), years of life lost (YLLs), and ASR of UC across 204 countries and regions worldwide. Over a span of three decades, from 1990 to 2019, this analysis identified critical years with the most marked shifts in these indicators, enabling the categorization of global patterns by age brackets and SDI. This analysis facilitated the delineation of trends on both regional and national scales. The projections for incidence and mortality rates of the coming 25 years are expected to yield insights that will be instrumental in informing policy development and preventive strategies.

## 2 Methods

### 2.1 Data source

This research aggregated UC data for the period 1990 to 2019, covering 204 countries and regions, extracted from the GBD 2019 public dataset available at http://ghdx.healthdata.org/gbd-results-tool (accessed on March 11, 2022). We adopted standardized methodologies to estimate a range of epidemiological indicators, such as incidence, prevalence, mortality, DALYs, YLDs, and YLLs, as well as their associated 95% uncertainty intervals (UI).The general methodology for estimating these epidemiological indicators has been elucidated in prior publications (4, 5).

### 2.2 Standard definitions

In the GBD 2019 dataset, UC is classified according to the International Classification of Diseases for Oncology, third edition [ICD-O-3] code as follows: C54, C54.0, C54.1, C54.2, C54.3, C54.4, C54.8, C54.9 (6). SDI is a composite indicator developed by researchers of the GBD to assess several key factors. These factors include the total fertility rate for individuals under the age of 25, mean education levels for those aged 15 and over, and income distribution lag. The index is measured on a scale from 0 to 1, with SDI values of 0 and 1 indicating the lowest and highest potential levels of development, each correlating with specific health outcomes. Based on SDI, geographic areas are categorized into SDI quintiles, encompassing High, High-middle, Middle, Low-middle, and Low SDI regions (7).

### 2.3 Statistical analysis

Incidence, prevalence, mortality, DALYs, YLDs, YLLs, along with their corresponding ASR, were used to assess trends in UC.ASR is particularly effective in adjusting for age-related variations among regions or countries with different age demographics. ASR was calculated using the direct method, referencing the World Health Organization (WHO) world standard population (2000-2025) utilized as the reference. The formula used for this calculation is as follows (8).


$$ASR=\frac{\sum_{i=1}^{A}a_{i}w_{i}}{\sum_{i=1}^{A}w_{i}}*100,000$$

$a_{i}$ represents the specific age ratio of the *i*th age group, $w_{i}$ represents the number (or weight) of the corresponding age group in the selected reference standard population, and A represents the number of age groups. Each ASR was reported per 100,000 population.

We employed the Average Annual Percentage Change (AAPC) to quantify temporal trends in ASR of UC for incidence, prevalence, mortality, DALYs, YLDs, and YLLs over the period spanning 1990 to 2019. The AAPC represents a singular metric derived from Joinpoint Regression Analysis, employing a weighted average of the Annual Percentage Change (APC) to detect continuous shifts in disease data across the study duration (9, 10).

The AAPC for each interval was computed as the weighted average of the slope of the linear regression line at the juncture point. Subsequently, this weighted average of the slope was converted into a percentage representing the annual change. Joinpoint employs a model that combines the most optimal fit of varying quantities of linear regressions, as outlined below:


$$\ln ASR=\;b_{0}+b_{1}x+c$$

$$AAPC=100*\left(\exp\left(b_{1}\right)-1\right)$$

x represents the calendar year. When AAPC values and their 95% confidence interval (CI) > 0, the trend is defined as increasing. In contrast, when AAPC values and their 95% CI< 0, the trend shows a downward trend. Otherwise, the burden is thought to be relatively stable over time.

This study calculated the Annual Average Percentage Changes (AAPCs) for four distinct time intervals: 1990–1999, 2000–2009, 2010–2019, and the full span from 1990 to 2019.Joinpoint regression analysis, also termed piecewise regression model, investigates the temporal trends of diseases by fitting the most parsimonious model that joins multiple linear segments on a logarithmic scale. The ‘Joinpoints’ are the transition points where different trend segments intersect, and each was evaluated using the Monte Carlo permutation method.

The final model was selected within the Joinpoint Trend Analysis Software, employing a combination of the Weighted Bayesian Information Criterion method and the expertise of the authors (11). The Nordpred analysis, conducted in five-year age-period-cohort intervals, forms the basis for projecting trend data for each period (12). In this study, the Nordpred analysis was applied to forecast the scenario of UC from 2020 to 2044, relying on the average trajectory derived from observed data. Additionally, we employed the Bayesian Age-Period-Cohort (BAPC) model, integrated with nested Laplace approximations, to validate the stability of the Nordpred analysis’s projected outcomes (13).

Considering the relatively stability of the annual SDI for each country, we opted for the SDI values of the 204 countries and regions in 2019 to represent the SDI for each country during the 2020-2044 period. This method improves the representativeness of our predictions and delineates the developmental patterns of UC across countries with diverse SDI levels.

## 3 Results

### 3.1 Global trends

**Figure 1:**
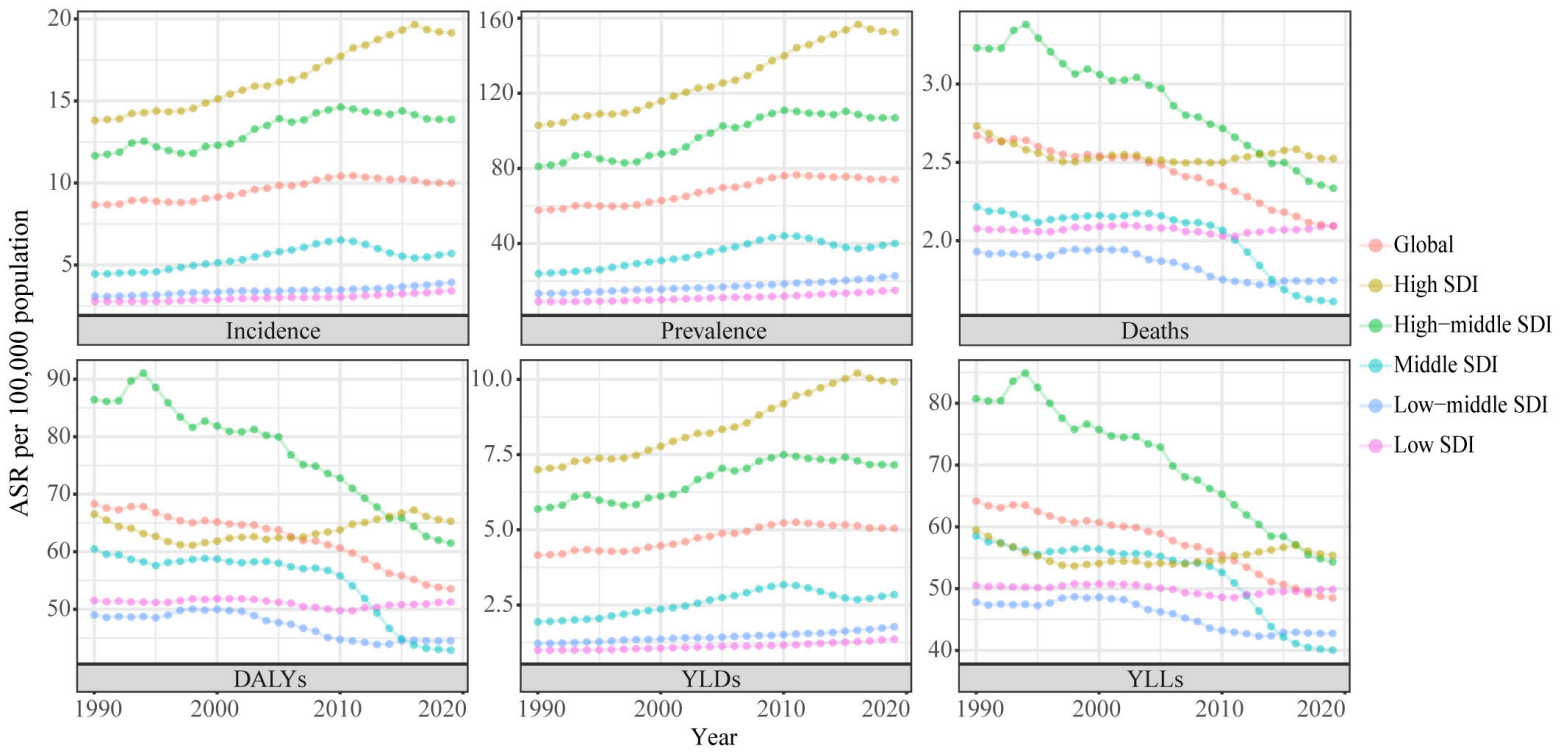
Trends in the global disease burden of uterine cancer, 1990-2019. ASR, age-standardized rates; DALYs, disability-adjusted life-years; YLDs, years lived with disability; YLLs, years of life lost; SDI, socio-demographic index.

**Figure 2:**
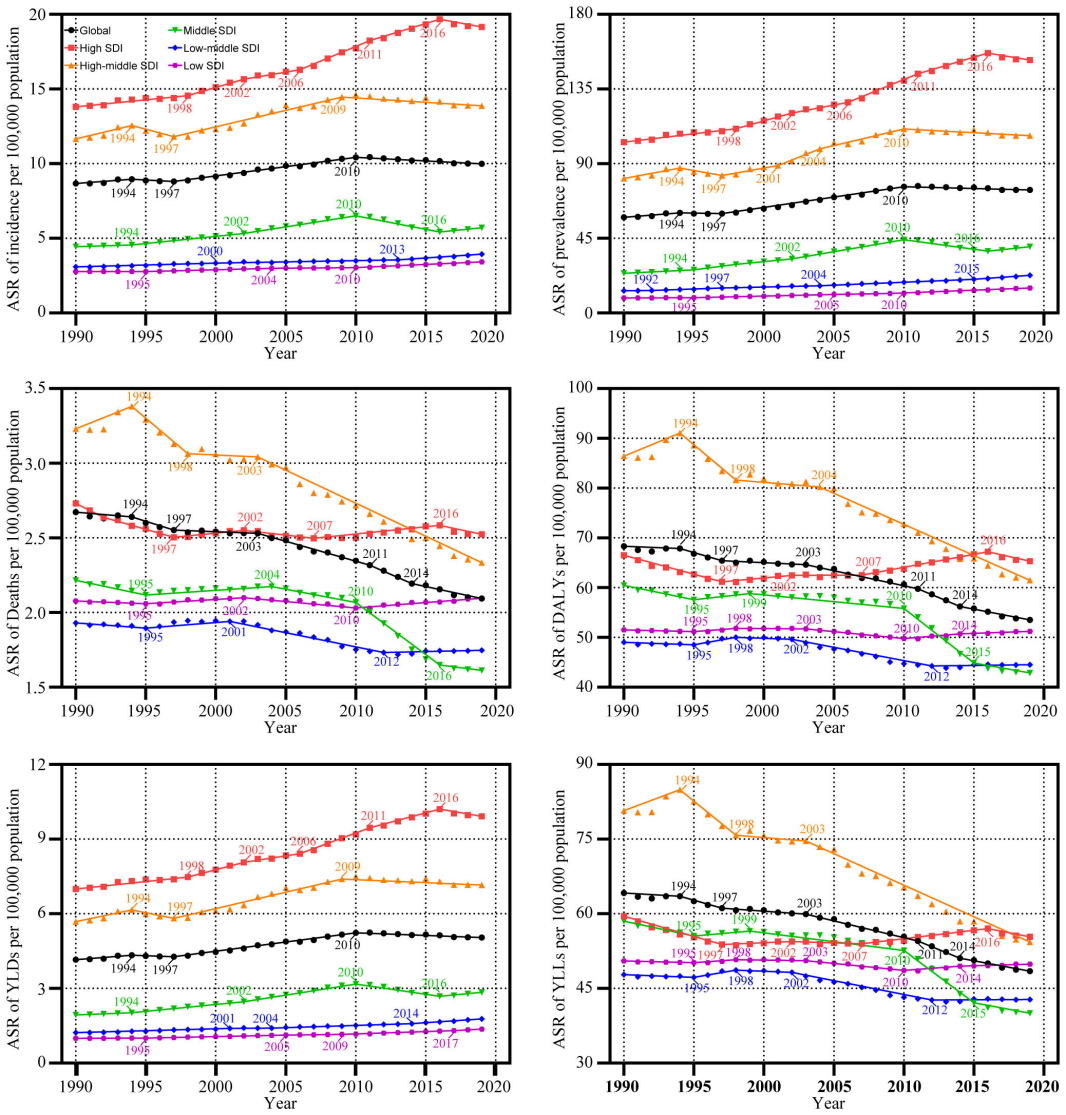
Trends in the global disease burden of uterine cancer by Joinpoint regression analysis. ASR, age-standardized rates; DALYs, disability-adjusted life-years; YLDs, years lived with disability; YLLs, years of life lost; SDI, socio-demographic index.

In the global context, the ASR of UC incidence has shown an overall increase over the past three decades, from 1990 to 2019 (AAPC: 0.50%, [95%CI: 0.31%, 0.69%]). The ASR rose from 8.67 (95% Uncertainty Interval (UI): 8.1, 9.08) per 100,000 population to 9.99 (95%UI: 9.12, 11.02) per 100,000 population during this timeframe. Notably, the trend exhibited an upward trajectory during 1990-1999 (AAPC: 0.50%, [95%CI: -0.08%, 1.08%]), followed by an accelerated increase during 2000-2009 (AAPC: 1.34%, [95%CI: 1.24%, 1.44%]). However, from 2010 to 2019, a downward trend emerged (AAPC: -0.51%, [95%CI: -0.67%, -0.36%]). ASRs for prevalence and Years Lived with Disability (YLDs) exhibited similar patterns (**Supplementary Table S1**; **Table 1**; **Figure 1**). Joinpoint regression analysis revealed distinct transition points in the ASR trends for incidence, prevalence, and YLDs in 1994, 1997, and 2010 (**Figure 2**; **Supplementary Table S2**).

**Table 1:** AAPCs in global and different SDI regions.

Year	Incidence		Prevalence		Deaths		DALYs		YLDs		YLLs	
	AAPC (%) (95% CI)	P value	AAPC (%) (95% CI)	P value	AAPC (%) (95% CI)	P value	AAPC (%) (95% CI)	P value	AAPC (%) (95% CI)	P value	AAPC (%) (95% CI)	P value
Global												
1990-2019	0.50 (0.31, 0.69)	<0.001*	0.88 (0.69, 1.07)	<0.001*	-0.84 (-1.03, -0.64)	<0.001*	-0.83 (-1.04, -0.62)	<0.001*	0.69 (0.49, 0.88)	<0.001*	-0.95 (-1.16, -0.75)	<0.001*
1990-1999	0.50 (-0.08, 1.08)	0.090	0.81 (0.23, 1.39)	0.006*	-0.51 (-0.94, -0.07)	0.022*	-0.47 (-0.93, 0.00)	0.048*	0.69 (0.09, 1.29)	0.023*	-0.55 (-0.99, -0.10)	0.017*
2000-2009	1.34 (1.24, 1.44)	<0.001*	1.94 (1.83, 2.04)	<0.001*	-0.76 (-0.89, -0.62)	<0.001*	-0.69 (-0.84, -0.54)	<0.001*	1.61 (1.50, 1.71)	<0.001*	-0.87 (-1.01, -0.73)	<0.001*
2010-2019	-0.51 (-0.67, -0.36)	<0.001*	-0.31 (-0.46, -0.16)	<0.001*	-1.30 (-1.71, -0.88)	<0.001*	-1.39 (-1.84, -0.95)	<0.001*	-0.43 (-0.59, -0.27)	<0.001*	-1.49 (-1.93, -1.06)	<0.001*
High SDI												
1990-2019	1.12 (0.91, 1.33)	<0.001*	1.34 (1.11, 1.57)	<0.001*	-0.27 (-0.36, -0.17)	<0.001*	-0.06 (-0.14, 0.02)	0.123	1.19 (0.95, 1.43)	<0.001*	-0.24 (-0.33, -0.16)	<0.001*
1990-1999	0.79 (0.62, 0.96)	<0.001*	1.07 (0.89, 1.26)	<0.001*	-0.84 (-0.95, -0.72)	<0.001*	-0.81 (-0.91, -0.71)	<0.001*	0.94 (0.75, 1.13)	<0.001*	-1.04 (-1.14, -0.94)	<0.001*
2000-2009	1.56 (1.15, 1.98)	<0.001*	1.86 (1.41, 2.31)	<0.001*	-0.10 (-0.27, 0.07)	0.238	0.29 (0.14, 0.43)	<0.001*	1.63 (1.17, 2.10)	<0.001*	0.09 (-0.07, 0.24)	0.268
2010-2019	0.79 (0.42, 1.17)	<0.001*	0.89 (0.48, 1.29)	<0.001*	-0.01 (-0.18, 0.15)	0.861	0.18 (0.04, 0.33)	0.011*	0.79 (0.37, 1.21)	<0.001*	0.07 (-0.07, 0.21)	0.343
High-middle SDI												
1990-2019	0.63 (0.23, 1.04)	0.002*	0.98 (0.45, 1.51)	<0.001*	-1.10 (-1.32, -0.88)	<0.001*	-1.16 (-1.41, -0.91)	<0.001*	0.83 (0.41, 1.24)	<0.001*	-1.36 (-1.61, -1.10)	<0.001*
1990-1999	0.54 (-0.69, 1.78)	0.392	0.70 (-0.43, 1.83)	0.225	-0.48 (-1.07, 0.10)	0.107	-0.48 (-1.16, 0.20)	0.167	0.71 (-0.55, 1.99)	0.272	-0.56 (-1.24, 0.12)	0.106
2000-2009	1.86 (1.61, 2.11)	<0.001*	2.46 (1.37, 3.55)	<0.001*	-1.19 (-1.43, -0.95)	<0.001*	-1.17 (-1.43, -0.90)	<0.001*	2.19 (1.94, 2.45)	<0.001*	-1.48 (-1.76, -1.21)	<0.001*
2010-2019	-0.51 (-0.79, -0.22)	0.001*	-0.4 (-0.69, -0.10)	0.013	-1.66 (-1.75, -1.57)	<0.001*	-1.85 (-1.96, -1.74)	<0.001*	-0.42 (-0.71, -0.13)	0.007*	-2.05 (-2.15, -1.95)	<0.001*
Middle SDI												
1990-2019	0.86 (0.72, 1.00)	<0.001*	1.79 (1.62, 1.96)	<0.001*	-1.09 (-1.18, -1.00)	<0.001*	-1.20 (-1.32, -1.08)	<0.001*	1.33 (1.18, 1.49)	<0.001*	-1.32 (-1.44, -1.19)	<0.001*
1990-1999	1.39 (1.16, 1.62)	<0.001*	2.51 (2.23, 2.79)	<0.001*	-0.33 (-0.45, -0.21)	<0.001*	-0.26 (-0.54, 0.03)	0.078	1.93 (1.68, 2.19)	<0.001*	-0.33 (-0.62, -0.04)	0.028*
2000-2009	2.53 (2.37, 2.68)	<0.001*	3.81 (3.61, 4.00)	<0.001*	-0.33 (-0.45, -0.21)	<0.001*	-0.38 (-0.47, -0.28)	<0.001*	3.15 (2.97, 3.32)	<0.001*	-0.55 (-0.65, -0.45)	<0.001*
2010-2019	-1.61 (-1.92, -1.30)	<0.001*	-1.28 (-1.66, -0.89)	<0.001*	-2.78 (-2.97, -2.58)	<0.001*	-3.11 (-3.36, -2.87)	<0.001*	-1.38 (-1.72, -1.03)	<0.001*	-3.22 (-3.47, -2.97)	<0.001*
Low-middle SDI												
1990-2019	0.87 (0.79, 0.96)	<0.001*	1.84 (1.70, 1.99)	<0.001*	-0.33 (-0.43, -0.23)	<0.001*	-0.31 (-0.48, -0.14)	<0.001*	1.33 (1.15, 1.51)	<0.001*	-0.36 (-0.54, -0.18)	<0.001*
1990-1999	0.93 (0.80, 1.07)	<0.001*	1.65 (1.29, 2.02)	<0.001*	0.08 (-0.14, 0.29)	0.476	0.25 (-0.20, 0.71)	0.274	1.29 (1.18, 1.40)	<0.001*	0.23 (-0.24, 0.69)	0.339
Low-middle SDI												
2000-2009	0.43 (0.32, 0.53)	<0.001*	1.65 (1.54, 1.76)	<0.001*	-0.98 (-1.07, -0.88)	<0.001*	-1.00 (-1.16, -0.83)	<0.001*	0.95 (0.43, 1.47)	<0.001*	-1.06 (-1.22, -0.89)	<0.001*
2010-2019	1.30 (1.11, 1.49)	<0.001*	2.28 (2.10, 2.47)	<0.001*	-0.09 (-0.23, 0.05)	0.195	-0.08 (-0.21, 0.06)	0.262	1.76 (1.56, 1.97)	<0.001*	-0.14 (-0.28, -0.01)	0.037*
Low SDI												
1990-2019	0.72 (0.67, 0.78)	<0.001*	1.76 (1.67, 1.84)	<0.001*	0.03 (-0.02, 0.08)	0.288	-0.02 (-0.10, 0.06)	0.671	1.09 (1.01, 1.18)	<0.001*	-0.04 (-0.12, 0.04)	0.288
1990-1999	0.40 (0.30, 0.51)	<0.001*	1.01 (0.87, 1.15)	<0.001*	0.04 (-0.06, 0.14)	0.460	0.06 (-0.12, 0.24)	0.528	0.61 (0.51, 0.70)	<0.001*	0.05 (-0.14, 0.23)	0.606
2000-2009	0.50 (0.40, 0.60)	<0.001*	1.68 (1.52, 1.84)	<0.001*	-0.26 (-0.34, -0.18)	<0.001*	-0.39 (-0.46, -0.31)	<0.001*	0.83 (0.66, 0.99)	<0.001*	-0.41 (-0.49, -0.33)	<0.001*
2010-2019	1.32 (1.24, 1.39)	<0.001*	2.62 (2.51, 2.72)	<0.001*	0.32 (0.25, 0.39)	<0.001*	0.34 (0.20, 0.47)	<0.001*	1.79 (1.61, 1.96)	<0.001*	0.30 (0.17, 0.43)	<0.001*

AAPC, average annual percentage change; DALYs, disability-adjusted life-years; YLDs, years lived with disability; YLLs, years of life lost; SDI, socio-demographic index; CI, confidence interval; *: P<0.05.

### 3.2 SDI trends

From 1990 to 2019, the ASR of UC incidence experienced an upward trajectory across all SDI regions. Notably, this upward trend was most pronounced in regions with High SDI regions (AAPC: 1.12%, [95%CI: 0.91%, 1.33%]). However, a notable shift occurred from 2010 to 2019 in the High SDI, High-middle SDI, and Middle SDI regions, where the incidence rates showed a deceleration or decline. In contrast, the Low-middle and Low-SDI regions experienced a significant increase in incidence rates during the same period. These trends in incidence were paralleled by similar patterns in prevalence and YLDs (**Supplementary Table S1**; **Table 1**; **Figure 1**).

Between 1990 and 2019, the ASR for mortality of UC decreased in all SDI regions, except the Low SDI regions. The greatest declines in mortality were seen in the High-middle and Middle SDI regions, with the Middle SDI region experiencing the most significant reduction from 2010 to 2019 (AAPC: -2.78%, [95%CI: -2.97%, -2.58%]). Significantly, the lowest ASR among the various SDI regions was observed during the 2010-2019 period. Conversely, in the Low SDI regions, the AAPC results indicated a stable ASR of mortality from 1990 to 2019 (P >= 0.05), though this stability was marked by fluctuations. Specifically, the ASR of mortality remained relatively unchanged from 1990 to 1999 (P >= 0.05). It exhibited a significant decline during 2000-2009 (AAPC: -0.26%, [95%CI: -0.34%, -0.18%]), and demonstrated a noteworthy increase from 2000-2009 (AAPC: 0.32%, [95%CI: 0.25%, 0.39%]). DALYs and YLLs exhibited trends similar to that of mortality (**Supplementary Table S1**; **Table 1**; **Figure 1**). Detailed results of the Joinpoint regression analysis for different SDI regions are presented in **Figure 2**; **Supplementary Table S2**.

### 3.3 Global trends in ages groups

Over the last three decades, from 1990 to 2019, there has been a noticeable trend in the incidence rates and YLDs for UC, which have either increased or remained stable across all age groups. The highest incidence rate was observed in the 55-59 age group (AAPC of incidence: 0.76%, [95%CI: 0.41%, 1.12%]), with rates rising from 28.91 per 100,000 population [95% UI: 26.72, 30.77] in 1990 to 35.71 per 100,000 population [95% UI: 32.27, 39.84] in 2019. Similarly, the rate of YLDs increased from 14.52 per 100,000 population [95% UI: 10.2, 19.54] in 1990 to 18.56 per 100,000 population [95% UI: 12.9, 25.17] in 2019. The prevalence rate of UC across all age groups has either increased or remained unchanged. The age group of 90-94 years experienced the greatest increase in rates, from 36.05 per 100,000 population [95% UI: 28.57, 40.02] in 1990 to 56.34 per 100,000 population [95% UI: 42.87, 65.24] in 2019 (AAPC: 1.60%, [95% CI: 1.42%, 1.78%]). From 1990 to 2019, the rates of deaths, DALYs, and YLLs due to UC either decreased or remained stable across all age groups. Notably, the most substantial declines were observed in the 20-24 age group (AAPC of Deaths: -1.96%, [95% CI: -2.53%, -1.40%]; AAPC of DALYs: -1.80%, [95% CI: -2.36%, -1.25%]; AAPC of YLLs: -1.96%, [95% CI: -2.52%, -1.40%]). The death rate per 100,000 population decreased from 0.06 [95% UI: 0.04, 0.08] in 1990 to 0.03 [95% UI: 0.02, 0.04] in 2019. The DALYs rate per 100,000 population decreased from 4.33 [95% UI: 2.52, 5.41] in 1990 to 2.47[95% UI: 1.68, 2.85] in 2019. Additionally, the YLLs rate per 100,000 population decreased from 4.13 [95% UI: 2.37, 5.18] in 1990 to 2.26 [95% UI: 1.54, 2.59] in 2019 (**Table 2**; **Supplementary Table S3**).

**Table 2:** AAPCs in different age groups, 1990-2019.

Age	Incidence		Prevalence		Deaths		DALYs		YLDs		YLLs	
	AAPC (%) (95% CI)	P value	AAPC (%) (95% CI)	P value	AAPC (%) (95% CI)	P value	AAPC (%) (95% CI)	P value	AAPC (%) (95% CI)	P value	AAPC (%) (95% CI)	P value
20 to 24	0.08 (-0.58, 0.75)	0.8	0.24 (-0.40, 0.89)	0.463	-1.96 (-2.53, -1.40)	<0.001*	-1.80 (-2.36, -1.25)	<0.001*	0.17 (-0.26, 0.60)	0.428	-1.96 (-2.52, -1.40)	<0.001*
25 to 29	0.29 (-0.65, 1.25)	0.5	0.46 (-0.45, 1.38)	0.320	-1.85 (-2.78, -0.90)	<0.001*	-1.55 (-2.24, -0.86)	<0.001*	0.39 (-0.54, 1.32)	0.412	-1.85 (-2.78, -0.90)	<0.001*
30 to 34	0.69 (-0.06, 1.45)	0.1	0.86 (0.17, 1.54)	0.014*	-1.49 (-2.31, -0.66)	<0.001*	-1.33 (-2.21, -0.45)	0.003*	0.78 (0.06, 1.51)	0.034*	-1.49 (-2.32, -0.65)	0.001*
35 to 39	0.47 (0.16, 0.77)	0.003*	0.67 (0.37, 0.97)	<0.001*	-1.66 (-1.87, -1.44)	<0.001*	-1.50 (-1.75, -1.24)	<0.001*	0.58 (0.28, 0.89)	<0.001*	-1.65 (-1.91, -1.40)	<0.001*
40 to 44	0.60 (0.35, 0.86)	<0.001*	0.86 (0.64, 1.08)	<0.001*	-1.23 (-1.40, -1.07)	<0.001*	-1.11 (-1.26, -0.95)	<0.001*	0.75 (0.53, 0.96)	<0.001*	-1.23 (-1.40, -1.07)	<0.001*
45 to 49	0.65 (0.07, 1.23)	0.028*	0.89 (0.37, 1.42)	0.001*	-1.15 (-1.76, -0.54)	<0.001*	-1.01 (-1.62, -0.40)	0.001*	0.77 (0.21, 1.33)	0.007*	-1.15 (-1.76, -0.54)	<0.001*
50 to 54	0.51 (0.32, 0.70)	<0.001*	0.72 (0.52, 0.92)	<0.001*	-1.30 (-1.42, -1.18)	<0.001*	-1.11 (-1.26, -0.96)	<0.001*	0.62 (0.43, 0.82)	<0.001*	-1.30 (-1.42, -1.18)	<0.001*
55 to 59	0.76 (0.41, 1.12)	<0.001*	1.02 (0.71, 1.33)	<0.001*	-1.02 (-1.21, -0.83)	<0.001*	-0.85 (-1.26, -0.43)	<0.001*	0.89 (0.57, 1.20)	<0.001*	-1.02 (-1.21, -0.83)	<0.001*
60 to 64	0.57 (0.27, 0.88)	<0.001*	0.90 (0.61, 1.20)	<0.001*	-0.86 (-1.15, -0.57)	<0.001*	-0.73 (-1.03, -0.44)	<0.001*	0.74 (0.43, 1.04)	<0.001*	-0.86 (-1.15, -0.56)	<0.001*
65 to 69	0.36 (0.17, 0.55)	<0.001*	0.73 (0.47, 1.00)	<0.001*	-0.80 (-1.01, -0.59)	<0.001*	-0.70 (-0.94, -0.47)	<0.001*	0.53 (0.19, 0.88)	0.002*	-0.80 (-1.01, -0.59)	<0.001*
70 to 74	0.44 (0.28, 0.59)	<0.001*	0.98 (0.85, 1.11)	<0.001*	-0.69 (-0.85, -0.53)	<0.001*	-0.60 (-0.76, -0.43)	<0.001*	0.68 (0.48, 0.88)	<0.001*	-0.69 (-0.85, -0.53)	<0.001*
75 to 79	0.06 (-0.21, 0.33)	0.6	0.68 (0.33, 1.03)	<0.001*	-0.87 (-0.97, -0.76)	<0.001*	-0.79 (-0.90, -0.68)	<0.001*	0.32 (0.03, 0.61)	0.032*	-0.88 (-0.98, -0.77)	<0.001*
80 to 84	0.25 (0.04, 0.46)	0.022*	1.10 (0.91, 1.29)	<0.001*	-0.58 (-0.69, -0.47)	<0.001*	-0.52 (-0.63, -0.40)	<0.001*	0.59 (0.37, 0.81)	<0.001*	-0.59 (-0.70, -0.49)	<0.001*
85 to 89	0.24 (-0.03, 0.51)	0.1	1.24 (0.98, 1.50)	<0.001*	-0.51 (-0.72, -0.30)	<0.001*	-0.45 (-0.66, -0.24)	<0.001*	0.64 (0.35, 0.92)	<0.001*	-0.52 (-0.73, -0.31)	<0.001*
90 to 94	0.28 (0.05, 0.50)	0.017*	1.60 (1.42, 1.78)	<0.001*	-0.24 (-0.39, -0.08)	0.003*	-0.21 (-0.37, -0.05)	0.009*	0.70 (0.50, 0.91)	<0.001*	-0.25 (-0.41, -0.1)	0.002*
95 plus	0.52 (0.44, 0.61)	<0.001*	0.74 (0.63, 0.84)	<0.001*	-0.01 (-0.12, 0.11)	0.896	-0.05 (-0.18, 0.09)	0.495	0.64 (0.53, 0.74)	<0.001*	-0.07 (-0.20, 0.06)	0.310

AAPC, average annual percentage change; DALYs, disability-adjusted life-years; YLDs, years lived with disability; YLLs, years of life lost; CI, confidence interval; *: P<0.05.

### 3.4 National trends

**Figure 3:**
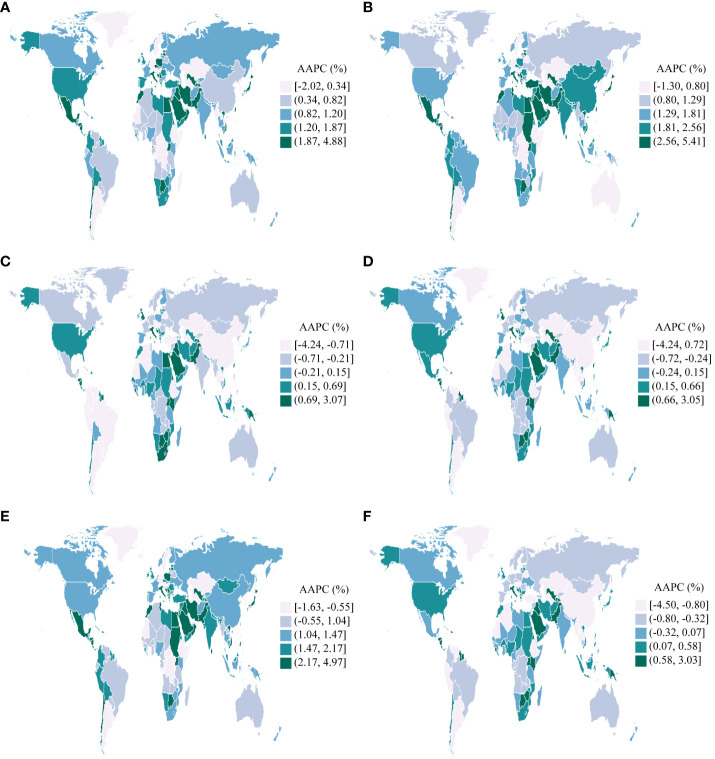
AAPCs in 204 countries and territories, 1990-2019. **(A)**: Incidence; **(B)**: Prevalence; **(C)**: Deaths; **(D)**: Disability-Adjusted Life Years(DALYs); **(E)**: Years Lived with Disability(YLDs); **(F)**: Years of Life Lost(YLLs).

From 1990 to 2019, across 204 countries and regions, the incidence of UC as measured by the ASR increased in 165 countries, decreased in 9 countries, and remained stable in 30 countries between 1990 and 2019(p-value for the AAPC >= 0.05). Similarly, the ASR of prevalence increased in 184 countries, decreased in 5 countries, and remained stable in 15 countries. The ASR of deaths increased in 77 countries, decreased in 72 countries, and remained stable in 55 countries. Concurrently, the ASR of DALYs increased in 70 countries, decreased in 77 countries, and remained stable in 57 countries. Furthermore, the ASR of YLDs increased in 177 countries, decreased in 6 countries, and remained stable in 21 countries. In conclusion, the ASR of YLLs increased in 62 countries, decreased in 88 countries, and remained stable in 54 countries (**Figure 3**).

Taiwan (Province of China) experienced the most significant increases in the ASR of incidence, prevalence, and YLDs for UC among the 204 countries and regions assessed between 1990 and 2019. (AAPC of incidence: 4.88%, [95%CI: 3.92%, 5.84%]; AAPC of prevalence: 5.41%, [95%CI: 4.66%, 6.16%]; AAPC of YLDs: 4.97%, [95%CI: 4.00%, 5.95%]). Contrarily, Turkmenistan experienced the most substantial decreases during the same period (AAPC of incidence: -2.02%, [95%CI: -2.53%, -1.50%]; AAPC of prevalence: -1.30%, [95%CI: -2.02%, -0.56%]; AAPC of YLDs: -1.63%, [95%CI: -2.24%, -1.01%]). Furthermore, Jamaica exhibited the most significant increases in the ASR of deaths, DALYs, and YLLs for UC (AAPC of deaths: 3.07%, [95%CI: 2.11%, 4.04%]; AAPC of DALYs: 3.05%, [95%CI: 2.14%, 3.96%]; AAPC of YLLs: 3.03%, [95%CI: 2.10%, 3.96%]). In a notable contrast, the Republic of Korea exhibited the most substantial reductions in the ASR of deaths, DALYs, and YLLs (AAPC of deaths: -4.24%, [95%CI: -4.53%, -3.94%]; AAPC of DALYs: -4.24%, [95%CI: -4.52%, -3.96%]; AAPC of YLLs: -4.50%, [95%CI: -4.77%, -4.23%]) (**Figure 3**).

### 3.5 GBD region trends

**Figure 4:**
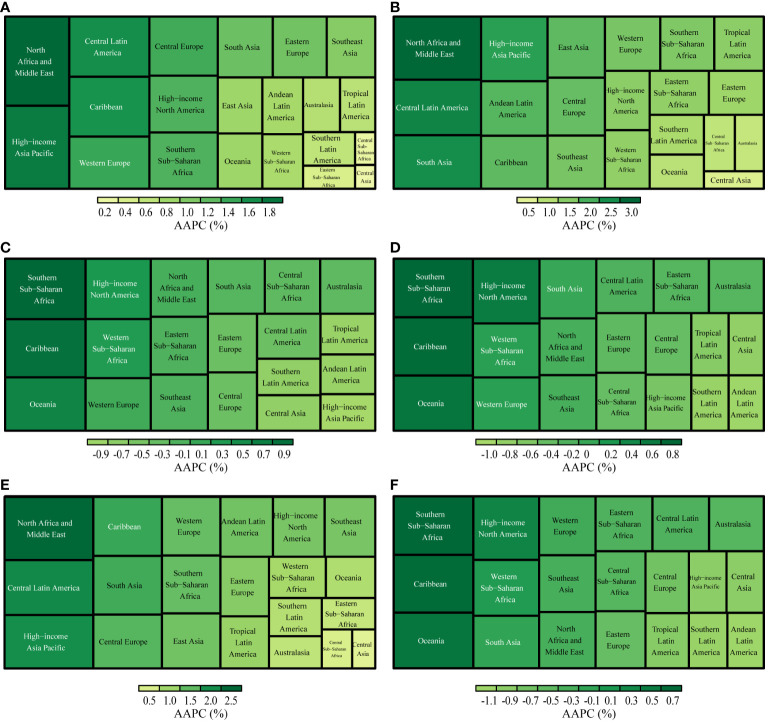
AAPCs in GBD regions, 1990-2019. **(A)**: Incidence; **(B)**: Prevalence; **(C)**: Deaths; **(D)**: Disability-Adjusted Life Years(DALYs); **(E)**: Years Lived with Disability(YLDs); **(F)**: Years of Life Lost(YLLs).

Between 1990 and 2019, the ASR of UC incidence, prevalence, and YLD either increased or remained stable across GBD regions, with p-values for the AAPC >= 0.05%. Significantly, the High-income Asia Pacific region observed the most pronounced increase in UC incidence (AAPC: 1.77%, [95%CI: 1.44%, 2.11%]). Similarly, the North Africa and Middle East region recorded the highest rates of UC prevalence and YLDs (AAPC of prevalence: 2.94%, [95%CI: 2.68%, 3.19%]; AAPC of YLDs: 2.37%, [95%CI: 2.12%, 2.63%]). Conversely, Central Asia exhibited the smallest increases in the ASR of incidence, prevalence, and YLDs (AAPC of incidence: 0.16%, [95%CI: 0.00%, 0.32%]; AAPC of prevalence:0.56%, [95%CI: 0.40%, 0.73%]; AAPC of YLDs: 0.38%, [95%CI: 0.22%, 0.53%]) (**Figure 4**).

From 1990 to 2019, the ASR of deaths, DAYLs, and YLLs associated with UC exhibited a decreasing trend in most GBD regions. The most significant decrease in the ASR of deaths was observed in the High-income Asia Pacific region (AAPC: -0.96%, [95%CI: -1.13%, -0.79%]). Furthermore, Andean Latin America observed the largest reduction in the ASR of DAYLs and YLLs (AAPC of DAYLs: -0.96%, [95%CI: -1.07%, -0.85%]; AAPC of YLLs: -1.06%, [95%CI: -1.17%, -0.95%]) (**Figure 4**).

### 3.6 Global uterine cancer incidence and mortality rate projections

**Figure 5:**
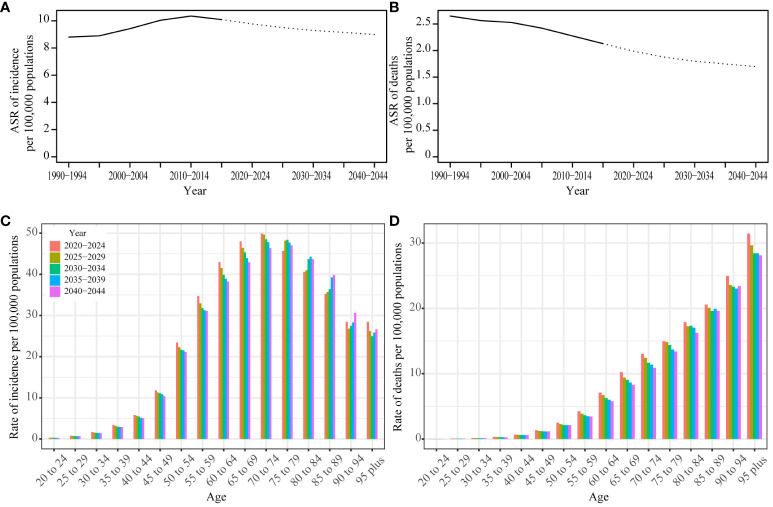
Prediction result by Norpred Prediction ASR result of incidence **(A)** and deaths **(B)**: observed is solid lines and predicted rates of the Norpred model is dashed lines, the blue region shows the upper and lower limits of the 95% UIs. Prediction different ages rate result of incidence **(C)** and deaths **(D)**.

We employed the Nordpred model to project UC incidence and mortality rates from 2020 to 2044. Our analysis indicates a sustained decrease in the ASR for both incidence and deaths in the foreseeable future. An age-specific analysis of incidence revealed the highest rates within the 70-79 age group. Notably, we observed a consistent yearly decline in incidence rates among individuals aged 20-74. In contrast, those in the 75-84 age group experienced an initial increase, followed by a subsequent decrease. Meanwhile, individuals aged 85 to 89 encountered a year-over-year rise in incidence rates. For individuals aged 90 years or older, the incidence rate first showed a decline, then reversed to an upward trend. Analyzing death rates across different age groups, we observed the highest mortality rate in the 95-plus age group. Mortality rates showed fluctuations among individuals aged 90 years, but for other age groups, they consistently decreased over time (**Figure 5**; **Supplementary Table S6**).

To assess the reliability of our predictions, we also applied the BAPC model to forecast UC incidence and mortality. Notably, the BAPC model yielded results that closely mirrored the trends identified by the Nordpred model (**Supplementary Figure S1**, **Supplementary Table S7**), further substantiating the robustness of our projections.

## 4 Discussion

To our knowledge, this represents the inaugural research detailing the global time trends and future predictions for uterine cancer from 1990 to 2019 across 204 countries and regions.

We conduct a comprehensive analysis and discussion on the prevalence and mortality rates, segmented by regions and age groups, and we project the incidence and death rates for the next 25 years. From 1990 to 2019, the global incidence of UC demonstrated an upward trend, with the most significant increase observed between 2000 and 2009, indicated by the AAPC of 1.34%. Regions with High and High-middle SDI demonstrated significantly higher ASR of incidence per 100,000 population, mirroring the patterns observed globally. During these thirty years, there has been a discernible decline in the worldwide mortality rate, as indicated by an AAPC of -0.84%. From 2010 to 2019, the High-middle SDI region experienced the most substantial decline, as measured by an AAPC of -1.30%. Particularly, since 1994, mortality rates in this region have decreased significantly. However, there have been no significant changes in the indicators for the Low-middle and Low SDI regions throughout these 30 years. Regions with a High SDI exhibit higher incidence rates but lower mortality rates. Conversely, areas with Low or Low-middle SDI display lower incidence rates, yet the reduction in mortality is less pronounced. These conspicuous distinctions arise from a multitude of factors.

Factors contributing to the development of UC include elevated estrogen levels, attributable to conditions such as obesity (14), diabetes, a diet high in fat, and Polycystic Ovary Syndrome (PCOS); nulliparity; early onset of menstruation; the late onset of menopause; the presence of Lynch syndrome, being within the age range of 55 to 64 years; and the use of tamoxifen (15). Large epidemiological studies show that obesity, hormonal imbalances, and other metabolic factors are crucial risk factors for uterine cancer (16). Health issues are more prevalent in regions characterized by High SDI and High-middle SDI levels, because of aging populations, declining fertility rates, and rising obesity levels, among other factors. The primary consideration is the presence of comprehensive healthcare systems in these regions, which contribute to an increased reported incidence of UC through the facilitation of early detection and diagnosis. Consequently, UC is the most common gynecological cancer in High SDI regions (17). The notable decrease in UC mortality rates in High and High-middle SDI regions is largely due to the use of advanced medical technologies and the presence of comprehensive healthcare systems. Tailored treatment strategies are developed based on molecular analysis and genetic factors, including hormonal therapy, chemotherapy, vaginal brachytherapy, adjuvant pelvic radiotherapy, immunotherapy, and systemic therapy, with special emphasis on post-treatment surveillance. Immunotherapy combined with chemotherapy, specifically pembrolizumab/carboplatin/paclitaxel and dostarlimab/carboplatin/paclitaxel regimens, is the preferred first-line treatment for advanced stages of the disease (18, 19). Additionally, annual endometrial biopsies are recommended for those with Lynch syndrome and their relatives to assess cancer risk, highlighting the role of preventative measures (20). Surgical precision and the advancement in robotic surgery, supported by high-end medical devices, contribute to higher success rates and fewer complications. The standardized approach to treatment in these regions, driven by substantial research investment, not only improves patient outcomes but also sets a precedent for the global management of UC.

Abnormal uterine bleeding or postmenopausal vaginal hemorrhage are the main indicators of UC, which can be recognized and treated in the initial phases of the condition, leading to improved outcomes. Regions classified as High SDI, with access to advanced medical resources, along with those classified as High-middle and Middle SDI, where medical standards are continuously improving, have experienced significant decreases in mortality rates. This has resulted in a consistent year-over-year decrease in the global mortality rate Low SDI regions often present unique environmental conditions and lifestyle choices compared to those in higher SDI regions, which may influence health outcomes. For example, lower obesity rates and healthier dietary patterns in these areas could potentially contribute to a reduced risk of uterine cancer. In contrast, over the past 30 years, Taiwan (Province of China), a high-income area within the Asia Pacific region, has experienced a significant increase in both the incidence and prevalence of uterine cancer. Meanwhile, Turkmenistan, located in Central Asia, has seen a notable decrease in these metrics. The younger median age of the population in Low SDI areas may also play a role in the lower incidence of UC. However, the limited medical infrastructure and diagnostic capabilities in lower SDI regions might lead to an underestimation of the true incidence rate of UC, as cases may not be fully identified and recorded. Additionally, despite some advancements, the mortality rate from UC in these regions has not seen a significant decrease and may have even increased over the past decade.

The ASR of deaths, DALYs, and YLLs due to UC has seen the most significant increase in Jamaica. Additionally, Southern Sub-Saharan Africa has been identified as the region experiencing the highest rise in the ASR of deaths, DALYs, and YLLs related to this condition.

Beyond the influences of economic development, racial differences also play a critical role in the prognosis of UC. The outcome of this condition is influenced by a variety of factors, including the tumor grade and depth of myometrial invasion, the patient’s age, the histopathologic type of cancer, lymph node involvement, tumor size, the presence of lymph vascular space invasion (LVSI), and invasion of the lower uterine segment. These factors collectively determine the disease’s progression and the patient’s overall prognosis (21). The Cancer Genome Atlas (TCGA) established four molecular categories of UC in 2013. These categories are microsatellite instability (MSI), copy-number low, copy-number high, and DNA polymerase epsilon catalytic subunit mutated (POLE) (22). UC exhibits significant racial disparities in mortality rates among common cancers (23, 24). Studies have shown that Black women with UC are often diagnosed at an earlier age but with more aggressive types of the disease, leading to advanced stages and poorer outcomes compared to White women (25). Serous histology and carcinosarcoma are more prevalent in Black patients with UC, whereas endometrioid cancer, a less aggressive type, is less common among them (26). Various factors contribute to the differences among racial groups, including genetic or molecular variations, socioeconomic status, access to specialized medical facilities, the time it takes to receive treatment, and the type of treatment provided.

Our analysis reveals that the incidence rate of UC escalates most rapidly among individuals aged 55-59, with an AAPC of 0.76%. Predictions from the Norpred and BAPC models indicate the highest incidence rates in the 70-79 age group. Meanwhile, rates for those aged 85-89 continue to rise annually. Predictive outcomes indicate that the incidence rate of UC will peak in the 70-79 age group in the future, highlighting an increased disease burden on the elderly population due to extended life expectancy and the cumulative effect of risk factors over time. Notably, the continuous rise in the incidence rate for the 85-89 age group suggests that the risk of UC does not stabilize but instead escalates even among the oldest demographics. This trend signifies that the healthcare system must be equipped to address not only the heightened risk of UC in the elderly but also the complexity and severity of the disease presentations in this cohort. To counter the rising trend of UC among the elderly, future healthcare planning must incorporate these considerations, adopting a comprehensive treatment approach that includes multidisciplinary care to meet the extensive health needs of older patients. This entails focusing on comorbidities and enhancing the overall quality of life of patients. In summary, as the aging population increases, the healthcare system must be prepared to meet the growing medical needs and challenges posed by the rising incidence of UC among the elderly.

In regions with High and High-middle SDI, obesity, diabetes, hypertension, and estrogen-induced metabolic syndrome are major risk factors for UC. To address these, primary prevention efforts focus on promoting healthy diets and physical activity. Furthermore, these regions are encouraged to adopt advanced screening and prevention strategies where possible. According to the classification by the TCGA, testing for high-risk genes is recommended. Notably, Black patients often have a higher incidence of serous-type tumors with high copy-number variations (CNV-high) and TP53 mutations (27). Conversely, Asian individuals are more prone to DNA mismatch repair gene mutations (28). Prevention strategies should, therefore, include management and regular screening for individuals at high risk, including those with conditions like Lynch syndrome (20). In the future, conducting tests for high-risk genes based on ethnicity to diagnose and prevent uterine cancer more early and accurately may become a focal point of work. This approach aims to tailor prevention and treatment strategies to effectively manage and reduce the risk of UC across diverse populations.

In Low-middle and Low SDI regions, limited healthcare resources significantly contribute to delays in diagnosing and treating diseases, notably UC. This lag between disease onset and treatment commencement is a major factor behind the higher mortality rates and poorer health outcomes observed in these areas. Addressing this issue, enhancing public awareness about the early detection and treatment of UC emerges as a critical intervention. By disseminating information on UC’s early symptoms, prevention methods, and screening procedures, these communities can better navigate their constrained medical landscapes. Media campaigns and face-to-face educational initiatives organized by community centers, schools, and religious institutions serve as effective platforms for encouraging proactive patient engagement with early screening and treatment options. This strategic approach not only promises to elevate treatment success and survival rates but also offers a practical solution to reducing healthcare expenses and patient financial burdens. Similarly, in terms of medical technology support, augmenting economic investments in accessible disease screening techniques, such as transvaginal ultrasound—a cost-effective and highly accurate diagnostic tool—emerges as a critical strategy for enhancing early detection rates and overall health outcomes in these regions with limited resources.

In 2009, the International Federation of Gynecology and Obstetrics (FIGO) updated the staging system for UC, introducing a new categorization within the IB stage and refining the stage’s grading. This revision enabled more precise assessments of patients’ conditions, leading to the development of more targeted treatment strategies. Subsequent analysis revealed noticeable shifts in incidence rates in 2009 and 2010, especially in High-middle and Middle SDI regions, indicating a worldwide impact. The adjustments in the surgical approach following the FIGO staging modification have been instrumental in reducing mortality rates and DALYs associated with UC. The treatment of UC mainly includes surgery, chemotherapy, hormone therapy, radiotherapy, and/or immunotherapy. Given the diverse treatment options, the customization of treatment plans for UC patients is of paramount importance. The National Comprehensive Cancer Network (NCCN) guidelines for 2023 elaborate on these specific treatment principles, offering a comprehensive framework to ensure that patients receive the most effective and personalized therapeutic strategies (20). Patients in economically disadvantaged situations often face a higher incidence of comorbidities and an increased risk of cancer recurrence (29, 30). Therefore, for patients in Low and Low-middle SDI regions, it is crucial to increase financial investments in healthcare, conduct regular post-operative follow-ups, and strengthen post-surgical management. These measures are essential for reducing both the mortality rate and the DALYs associated with diseases. Access to UC treatments should be universal, transcending race, ethnicity, or economic status. It’s also crucial to continuously promote national awareness campaigns for both the general public and medical professionals, highlighting the significance of detecting cancer early.

## 5 Conclusion

This study demonstrates the significant negative impact of UC on global health, influenced by various factors including geographical location, age, racial disparities, and the SDI. Predictions from the Norpred and BAPC models indicate that the incidence and mortality rates of uterine cancer are expected to decrease from 2020 to 2044. Given these findings, it is crucial for policymakers to develop targeted prevention and treatment strategies for uterine cancer and to take prompt action to reduce the burden of this condition.

## 6 Limitations

Although we used AAPC to eliminate the influence of age, some regions still lack high-quality data. Our prediction for UC was based on data prior to 2019, and the model did not incorporate the global impact of COVID-19 after 2019 into future predictions.

## Data availability statement

The datasets presented in this study can be found in online repositories. The names of the repository/repositories and accession number(s) can be found in the article/**Supplementary Material**.

## Ethics statement

This study does not require an ethical review because only large amounts of aggregated data without individual identifiers were used in the data analysis.

## Author contributions

SS: Conceptualization, Data curation, Formal analysis, Funding acquisition, Investigation, Methodology, Project administration, Resources, Software, Supervision, Validation, Visualization, Writing – original draft, Writing – review & editing. DZ: Funding acquisition, Resources, Visualization, Writing – review & editing. YW: Formal analysis, Project administration, Validation, Writing – original draft. ZS: Conceptualization, Data curation, Formal analysis, Funding acquisition, Investigation, Methodology, Project administration, Resources, Software, Supervision, Validation, Visualization, Writing – original draft, Writing – review & editing.
