# Supplementary material for: Changing trends in the disease burden of uterine cancer globally from 1990 to 2019 and its predicted level in 25 years
Source: Front Oncol. 2024 Apr 22;14:1361419. doi: 10.3389/fonc.2024.1361419 (PMC11070460; doi:10.3389/fonc.2024.1361419)
Supplement: Supplementary file 1 [file DataSheet_1.docx]

Table S1 Disease burden of uterine cancer in 1990 and 2019

| **Location** | **Number *10^4 (95%UI)** | | | | **Rate per 100,000 population (95%UI)** | | | | **ASR per 100,000 population (95%UI)** | | | |
| --- | --- | --- | --- | --- | --- | --- | --- | --- | --- | --- | --- | --- |
|  | **1990** | **2000** | **2010** | **2019** | **1990** | **2000** | **2010** | **2019** | **1990** | **2000** | **2010** | **2019** |
| **Incidence** |  |  |  |  |  |  |  |  |  |  |  |  |
| Global | 18.72 (17.46, 19.6) | 24.44 (22.4, 25.33) | 35.86 (31.69, 37.54) | 43.5 (39.7, 47.97) | 7.05 (6.57, 7.38) | 8 (7.33, 8.3) | 10.32 (9.12, 10.8) | 11.28 (10.29, 12.44) | 8.67 (8.1, 9.08) | 9.14 (8.41, 9.47) | 10.42 (9.24, 10.9) | 9.99 (9.12, 11.02) |
| High SDI | 7.75 (7.46, 7.94) | 9.76 (9.4, 10.01) | 13.42 (12.81, 13.82) | 16.8 (14.86, 18.86) | 18.58 (17.89, 19.04) | 21.85 (21.05, 22.41) | 27.92 (26.64, 28.75) | 33.1 (29.27, 37.14) | 13.82 (13.36, 14.15) | 15.12 (14.66, 15.49) | 17.73 (17.06, 18.2) | 19.16 (16.94, 21.48) |
| High-middle SDI | 7.05 (6.74, 7.37) | 8.81 (8.25, 9.14) | 12.9 (11.48, 13.57) | 14.91 (13.35, 16.5) | 12.19 (11.66, 12.74) | 13.94 (13.06, 14.47) | 18.96 (16.87, 19.95) | 20.83 (18.65, 23.05) | 11.66 (11.15, 12.19) | 12.3 (11.52, 12.79) | 14.63 (12.99, 15.4) | 13.87 (12.4, 15.37) |
| Middle SDI | 2.58 (1.99, 2.95) | 4.01 (2.89, 4.41) | 6.91 (4.65, 7.76) | 7.86 (6.47, 9.2) | 3.06 (2.35, 3.49) | 4.12 (2.97, 4.53) | 6.34 (4.27, 7.11) | 6.6 (5.43, 7.73) | 4.45 (3.48, 5.05) | 5.14 (3.78, 5.63) | 6.51 (4.46, 7.28) | 5.7 (4.72, 6.67) |
| Low-middle SDI | 0.99 (0.8, 1.18) | 1.4 (1.11, 1.6) | 1.97 (1.67, 2.29) | 2.94 (2.5, 3.56) | 1.78 (1.45, 2.13) | 2.12 (1.68, 2.42) | 2.55 (2.16, 2.96) | 3.35 (2.85, 4.06) | 3.09 (2.53, 3.71) | 3.35 (2.75, 3.85) | 3.49 (3.01, 4.11) | 3.94 (3.36, 4.8) |
| Low SDI | 0.34 (0.27, 0.42) | 0.45 (0.36, 0.54) | 0.62 (0.51, 0.77) | 0.95 (0.78, 1.17) | 1.29 (1.03, 1.62) | 1.31 (1.06, 1.59) | 1.39 (1.15, 1.71) | 1.69 (1.38, 2.09) | 2.78 (2.2, 3.5) | 2.9 (2.35, 3.55) | 3.04 (2.5, 3.74) | 3.43 (2.81, 4.21) |

| **Location** | **Number *10^4 (95%UI)** | | | | **Rate per 100,000 population (95%UI)** | | | | **ASR per 100,000 population (95%UI)** | | | |
| --- | --- | --- | --- | --- | --- | --- | --- | --- | --- | --- | --- | --- |
|  | **1990** | **2000** | **2010** | **2019** | **1990** | **2000** | **2010** | **2019** | **1990** | **2000** | **2010** | **2019** |
| **Prevalence** |  |  |  |  |  |  |  |  |  |  |  |  |
| Global | 126.04 (118.64, 130.97) | 169.33 (155.87, 174.99) | 262.81 (232.26, 275.04) | 322.32 (293.32, 354.11) | 47.45 (44.67, 49.31) | 55.45 (51.04, 57.3) | 75.62 (66.83, 79.14) | 83.58 (76.06, 91.82) | 57.74 (54.45, 59.99) | 62.89 (58.08, 64.96) | 76.09 (67.46, 79.54) | 74.14 (67.49, 81.48) |
| High SDI | 56.29 (54.6, 57.62) | 72.62 (70.48, 74.35) | 103.13 (99.27, 105.93) | 129.89 (114.91, 146.09) | 134.94 (130.88, 138.11) | 162.55 (157.75, 166.4) | 214.56 (206.52, 220.38) | 255.86 (226.35, 287.77) | 102.93 (99.89, 105.28) | 115.87 (112.81, 118.56) | 139.98 (135.43, 143.42) | 152.48 (134.64, 171.62) |
| High-middle SDI | 48.97 (46.94, 51.04) | 62.5 (58.43, 64.9) | 97.31 (86.09, 102.54) | 113.83 (101.73, 126.22) | 84.67 (81.17, 88.25) | 98.88 (92.44, 102.68) | 142.99 (126.51, 150.68) | 159 (142.11, 176.31) | 81.05 (77.54, 84.57) | 87.75 (81.96, 91.18) | 110.92 (97.95, 116.96) | 106.92 (95.4, 118.82) |
| Middle SDI | 14.76 (11.19, 16.91) | 25.31 (18.12, 28.03) | 48.41 (31.88, 54.73) | 56.17 (45.62, 66.11) | 17.48 (13.26, 20.03) | 26.01 (18.62, 28.8) | 44.4 (29.24, 50.2) | 47.17 (38.31, 55.53) | 23.99 (18.47, 27.38) | 30.91 (22.39, 34.13) | 44.15 (29.48, 49.73) | 40 (32.6, 46.99) |
| Low-middle SDI | 4.69 (3.73, 5.64) | 7.05 (5.43, 8.08) | 11.08 (9.16, 12.48) | 17.6 (14.92, 21) | 8.48 (6.75, 10.19) | 10.69 (8.23, 12.25) | 14.31 (11.82, 16.12) | 20.06 (17, 23.94) | 13.36 (10.73, 15.94) | 15.62 (12.2, 17.84) | 18.53 (15.52, 21.14) | 22.73 (19.34, 27.13) |
| Low SDI | 1.26 (1, 1.55) | 1.73 (1.4, 2.09) | 2.72 (2.21, 3.32) | 4.62 (3.76, 5.7) | 4.8 (3.81, 5.94) | 5.09 (4.13, 6.15) | 6.08 (4.94, 7.43) | 8.22 (6.69, 10.14) | 9.12 (7.25, 11.34) | 10.08 (8.2, 12.23) | 11.95 (9.83, 14.57) | 15.12 (12.34, 18.67) |

Table S1 (continued)

Table S1 (continued)

| **Location** | **Number *10^4 (95%UI)** | | | | **Rate per 100,000 population (95%UI)** | | | | **ASR per 100,000 population (95%UI)** | | | |  |
| --- | --- | --- | --- | --- | --- | --- | --- | --- | --- | --- | --- | --- | --- |
|  | **1990** | **2000** | **2010** | **2019** | **1990** | **2000** | **2010** | **2019** | **1990** | **2000** | **2010** | **2019** | |
| **Deaths** |  |  |  |  |  |  |  |  |  |  |  |  | |
| Global | 5.61 (5.11, 6.02) | 6.67 (5.97, 7.08) | 7.96 (6.92, 8.41) | 9.16 (8.24, 10.15) | 2.11 (1.92, 2.27) | 2.19 (1.95, 2.32) | 2.29 (1.99, 2.42) | 2.38 (2.14, 2.63) | 2.67 (2.44, 2.86) | 2.54 (2.27, 2.69) | 2.35 (2.05, 2.48) | 2.09 (1.88, 2.32) | |
| High SDI | 1.69 (1.59, 1.74) | 1.87 (1.73, 1.94) | 2.22 (2.01, 2.32) | 2.66 (2.4, 2.81) | 4.05 (3.81, 4.17) | 4.18 (3.87, 4.34) | 4.61 (4.19, 4.83) | 5.25 (4.73, 5.54) | 2.73 (2.57, 2.81) | 2.53 (2.38, 2.61) | 2.5 (2.32, 2.6) | 2.52 (2.32, 2.64) | |
| High-middle SDI | 1.95 (1.83, 2.07) | 2.23 (2.09, 2.33) | 2.44 (2.2, 2.58) | 2.64 (2.4, 2.88) | 3.37 (3.17, 3.57) | 3.52 (3.3, 3.69) | 3.59 (3.23, 3.79) | 3.69 (3.35, 4.03) | 3.23 (3.03, 3.42) | 3.06 (2.86, 3.21) | 2.72 (2.44, 2.87) | 2.33 (2.12, 2.55) | |
| Middle SDI | 1.18 (0.93, 1.35) | 1.53 (1.14, 1.69) | 2 (1.41, 2.2) | 2.1 (1.75, 2.43) | 1.4 (1.1, 1.6) | 1.58 (1.17, 1.74) | 1.83 (1.3, 2.01) | 1.76 (1.47, 2.04) | 2.22 (1.77, 2.52) | 2.16 (1.64, 2.38) | 2.07 (1.49, 2.26) | 1.61 (1.36, 1.87) | |
| Low-middle SDI | 0.56 (0.46, 0.68) | 0.74 (0.61, 0.87) | 0.91 (0.79, 1.11) | 1.22 (1.04, 1.53) | 1.01 (0.83, 1.22) | 1.12 (0.93, 1.32) | 1.18 (1.02, 1.43) | 1.4 (1.19, 1.74) | 1.93 (1.61, 2.36) | 1.95 (1.63, 2.31) | 1.75 (1.53, 2.17) | 1.75 (1.49, 2.21) | |
| Low SDI | 0.23 (0.18, 0.29) | 0.3 (0.24, 0.37) | 0.38 (0.31, 0.47) | 0.53 (0.43, 0.66) | 0.89 (0.7, 1.12) | 0.87 (0.7, 1.08) | 0.85 (0.69, 1.05) | 0.94 (0.77, 1.18) | 2.08 (1.64, 2.65) | 2.09 (1.69, 2.6) | 2.03 (1.65, 2.51) | 2.1 (1.72, 2.63) | |

Table S1 (continued)

| **Location** | **Number *10^4 (95%UI)** | | | | **Rate per 100,000 population (95%UI)** | | | | **ASR per 100,000 population (95%UI)** | | | |
| --- | --- | --- | --- | --- | --- | --- | --- | --- | --- | --- | --- | --- |
|  | **1990** | **2000** | **2010** | **2019** | **1990** | **2000** | **2010** | **2019** | **1990** | **2000** | **2010** | **2019** |
| **DALYs** |  |  |  |  |  |  |  |  |  |  |  |  |
| Global | 148.33 (131.75, 161.27) | 175.72 (152.52, 187.16) | 208.61 (175.58, 221.85) | 232.91 (209.29, 256.09) | 55.84 (49.6, 60.72) | 57.54 (49.95, 61.29) | 60.02 (50.52, 63.83) | 60.39 (54.27, 66.4) | 68.33 (60.86, 74.23) | 65.18 (56.88, 69.4) | 60.66 (51.27, 64.48) | 53.54 (48.13, 58.84) |
| High SDI | 38.01 (36.17, 39.56) | 41.02 (38.82, 43) | 49.87 (46.61, 52.66) | 59.68 (55.58, 63.61) | 91.11 (86.69, 94.82) | 91.82 (86.88, 96.24) | 103.75 (96.97, 109.55) | 117.55 (109.49, 125.29) | 66.52 (63.07, 69.26) | 61.85 (58.79, 64.91) | 63.79 (60, 67.33) | 65.31 (60.99, 69.65) |
| High-middle SDI | 52.32 (48.64, 55.87) | 59.17 (54.21, 62.26) | 64.25 (56.03, 68.51) | 66.79 (60.62, 73.52) | 90.47 (84.1, 96.6) | 93.61 (85.76, 98.5) | 94.41 (82.33, 100.67) | 93.3 (84.68, 102.7) | 86.43 (80.17, 92.33) | 81.87 (75.02, 86.32) | 72.79 (63.27, 77.56) | 61.5 (55.81, 67.85) |
| Middle SDI | 35.42 (26.69, 40.94) | 45.85 (32.84, 50.86) | 58.39 (39.22, 64.76) | 58.46 (47.58, 67.48) | 41.97 (31.63, 48.51) | 47.12 (33.75, 52.27) | 53.55 (35.97, 59.39) | 49.1 (39.96, 56.67) | 60.49 (46.35, 69.57) | 58.75 (42.92, 64.87) | 55.81 (38.07, 61.58) | 42.88 (34.87, 49.47) |
| Low-middle SDI | 15.94 (12.95, 19.36) | 21.26 (16.74, 24.66) | 25.44 (21.43, 29.57) | 33.22 (28.09, 40.49) | 28.81 (23.4, 34.97) | 32.23 (25.37, 37.39) | 32.85 (27.67, 38.17) | 37.86 (32.01, 46.14) | 49.01 (39.98, 59.48) | 49.96 (40.26, 58.11) | 44.74 (38.31, 52.91) | 44.53 (37.69, 54.57) |
| Low SDI | 6.54 (5.14, 8.24) | 8.28 (6.65, 10.1) | 10.49 (8.58, 12.79) | 14.53 (11.79, 18.15) | 24.98 (19.65, 31.46) | 24.31 (19.55, 29.67) | 23.43 (19.17, 28.58) | 25.82 (20.96, 32.27) | 51.5 (40.84, 65.01) | 51.83 (41.63, 63.92) | 49.77 (40.7, 61.13) | 51.24 (41.73, 64.02) |
|  |  |  |  |  |  |  |  |  |  |  |  |  |

Table S1 (continued)

| **Location** | **Number *10^4 (95%UI)** | | | | **Rate per 100,000 population (95%UI)** | | | | **ASR per 100,000 population (95%UI)** | | | |
| --- | --- | --- | --- | --- | --- | --- | --- | --- | --- | --- | --- | --- |
|  | **1990** | **2000** | **2010** | **2019** | **1990** | **2000** | **2010** | **2019** | **1990** | **2000** | **2010** | **2019** |
| **YLDs** |  |  |  |  |  |  |  |  |  |  |  |  |
| Global | 9.03 (6.47, 11.85) | 11.99 (8.51, 15.76) | 18.03 (12.76, 23.95) | 21.96 (15.54, 29.4) | 3.4 (2.44, 4.46) | 3.93 (2.79, 5.16) | 5.19 (3.67, 6.89) | 5.69 (4.03, 7.62) | 4.16 (2.99, 5.45) | 4.47 (3.17, 5.87) | 5.23 (3.7, 6.94) | 5.05 (3.57, 6.77) |
| High SDI | 3.89 (2.83, 5.09) | 4.96 (3.58, 6.55) | 6.88 (4.93, 9.07) | 8.59 (6.04, 11.42) | 9.33 (6.79, 12.19) | 11.11 (8.02, 14.65) | 14.31 (10.25, 18.87) | 16.92 (11.9, 22.49) | 7 (5.07, 9.22) | 7.77 (5.57, 10.32) | 9.19 (6.56, 12.14) | 9.92 (6.92, 13.28) |
| High-middle SDI | 3.44 (2.46, 4.55) | 4.38 (3.1, 5.77) | 6.6 (4.63, 8.82) | 7.66 (5.37, 10.24) | 5.95 (4.26, 7.86) | 6.92 (4.9, 9.12) | 9.7 (6.8, 12.97) | 10.7 (7.5, 14.3) | 5.69 (4.08, 7.52) | 6.12 (4.33, 8.07) | 7.5 (5.23, 10.04) | 7.16 (4.99, 9.61) |
| Middle SDI | 1.16 (0.76, 1.6) | 1.88 (1.2, 2.58) | 3.42 (2.04, 4.82) | 3.95 (2.72, 5.68) | 1.37 (0.9, 1.9) | 1.94 (1.23, 2.65) | 3.14 (1.87, 4.42) | 3.32 (2.28, 4.77) | 1.94 (1.29, 2.67) | 2.36 (1.53, 3.21) | 3.17 (1.92, 4.46) | 2.84 (1.96, 4.07) |
| Low-middle SDI | 0.41 (0.27, 0.57) | 0.59 (0.4, 0.81) | 0.88 (0.6, 1.21) | 1.35 (0.92, 1.87) | 0.73 (0.49, 1.04) | 0.9 (0.6, 1.23) | 1.13 (0.77, 1.56) | 1.54 (1.05, 2.14) | 1.22 (0.84, 1.71) | 1.37 (0.93, 1.87) | 1.51 (1.03, 2.08) | 1.78 (1.22, 2.48) |
| Low SDI | 0.13 (0.09, 0.19) | 0.17 (0.12, 0.24) | 0.25 (0.17, 0.36) | 0.4 (0.27, 0.57) | 0.49 (0.33, 0.71) | 0.5 (0.34, 0.7) | 0.56 (0.38, 0.8) | 0.7 (0.47, 1.02) | 1 (0.67, 1.45) | 1.06 (0.71, 1.49) | 1.16 (0.79, 1.66) | 1.37 (0.92, 1.96) |
|  |  |  |  |  |  |  |  |  |  |  |  |  |

Table S1 (continued)

| **Location** | **Number *10^4 (95%UI)** | | | | **Rate per 100,000 population (95%UI)** | | | | **ASR per 100,000 population (95%UI)** | | | |
| --- | --- | --- | --- | --- | --- | --- | --- | --- | --- | --- | --- | --- |
|  | **1990** | **2000** | **2010** | **2019** | **1990** | **2000** | **2010** | **2019** | **1990** | **2000** | **2010** | **2019** |
| **YLLs** |  |  |  |  |  |  |  |  |  |  |  |  |
| Global | 139.3 (123.74, 151.14) | 163.72 (141.64, 174.54) | 190.58 (160.51, 201.93) | 210.95 (190.19, 231.97) | 52.45 (46.59, 56.9) | 53.61 (46.38, 57.15) | 54.83 (46.18, 58.1) | 54.7 (49.32, 60.15) | 64.17 (57.3, 69.6) | 60.72 (52.81, 64.71) | 55.43 (46.84, 58.7) | 48.49 (43.73, 53.28) |
| High SDI | 34.12 (32.65, 34.97) | 36.06 (34.37, 37.07) | 43 (40.47, 44.45) | 51.09 (47.63, 53.27) | 81.78 (78.27, 83.83) | 80.71 (76.92, 82.97) | 89.45 (84.19, 92.47) | 100.64 (93.83, 104.92) | 59.52 (56.99, 60.96) | 54.08 (52.01, 55.5) | 54.6 (51.98, 56.2) | 55.4 (52.32, 57.62) |
| High-middle SDI | 48.88 (45.52, 52.24) | 54.79 (50.37, 57.47) | 57.65 (50.36, 60.87) | 59.13 (53.64, 64.78) | 84.52 (78.7, 90.32) | 86.69 (79.69, 90.92) | 84.71 (74, 89.45) | 82.6 (74.93, 90.49) | 80.75 (74.95, 86.38) | 75.75 (69.6, 79.63) | 65.29 (56.84, 69) | 54.34 (49.41, 59.54) |
| Middle SDI | 34.26 (25.83, 39.71) | 43.97 (31.58, 48.99) | 54.97 (36.99, 60.79) | 54.51 (44.28, 62.7) | 40.6 (30.61, 47.05) | 45.18 (32.45, 50.35) | 50.41 (33.92, 55.75) | 45.78 (37.19, 52.66) | 58.55 (45.04, 67.31) | 56.38 (41, 62.49) | 52.64 (36.12, 58.1) | 40.04 (32.65, 46.08) |
| Low-middle SDI | 15.54 (12.64, 18.87) | 20.67 (16.28, 24.04) | 24.56 (20.82, 28.7) | 31.87 (26.9, 38.6) | 28.07 (22.85, 34.1) | 31.33 (24.67, 36.45) | 31.71 (26.88, 37.05) | 36.32 (30.65, 43.99) | 47.79 (38.9, 57.97) | 48.59 (39.22, 56.43) | 43.23 (37.27, 51.1) | 42.76 (36.18, 52.19) |
| Low SDI | 6.41 (5.04, 8.06) | 8.1 (6.51, 9.89) | 10.24 (8.38, 12.48) | 14.13 (11.49, 17.55) | 24.49 (19.27, 30.79) | 23.81 (19.11, 29.05) | 22.88 (18.72, 27.89) | 25.12 (20.42, 31.21) | 50.5 (39.96, 63.61) | 50.77 (40.77, 62.59) | 48.61 (39.7, 59.43) | 49.87 (40.64, 62.32) |

ASR: age-standardized rates; DALYs: disability-adjusted life-years; YLDs: years lived with a disability; YLLs: years of life lost; SDI: socio-demographic index; UI: uncertainty interval.

Table S2 Joinpoint regression analysis results

| **Location** | **Segment** | **APC (%)** | **P-Value** |
| --- | --- | --- | --- |
| **Incidence** |  |  |  |
| *Global* | 1990-1994 | 0.91 (0.37, 1.45) | 0.002* |
|  | 1994-1997 | -0.59 (-2.27, 1.11) | 0.472 |
|  | 1997-2010 | 1.34 (1.24, 1.44) | <0.001* |
|  | 2010-2019 | -0.51 (-0.67, -0.36) | <0.001* |
| *High SDI* | 1990-1998 | 0.66 (0.47, 0.84) | <0.001* |
|  | 1998-2002 | 1.87 (1.02, 2.73) | <0.001* |
|  | 2002-2006 | 0.88 (0.04, 1.73) | 0.042* |
|  | 2006-2011 | 2.27 (1.73, 2.81) | <0.001* |
|  | 2011-2016 | 1.51 (0.97, 2.05) | <0.001* |
|  | 2016-2019 | -0.88 (-1.7, -0.04) | 0.041* |
| *High-middle SDI* | 1990-1994 | 2.01 (0.85, 3.19) | 0.002* |
|  | 1994-1997 | -2.25 (-5.73, 1.35) | 0.203 |
|  | 1997-2009 | 1.86 (1.61, 2.11) | <0.001* |
|  | 2009-2019 | -0.51 (-0.79, -0.22) | 0.001* |
| *Middle SDI* | 1990-1994 | 0.57 (0.08, 1.06) | 0.025* |
|  | 1994-2002 | 2.05 (1.85, 2.26) | <0.001* |
|  | 2002-2010 | 2.66 (2.45, 2.87) | <0.001* |
|  | 2010-2016 | -3.30 (-3.63, -2.96) | <0.001* |
|  | 2016-2019 | 1.84 (1.07, 2.63) | <0.001* |
| *Low-middle SDI* | 1990-2000 | 0.93 (0.80, 1.07) | <0.001* |
|  | 2000-2013 | 0.43 (0.32, 0.53) | <0.001* |
|  | 2013-2019 | 1.74 (1.44, 2.04) | <0.001* |
| *Low SDI* | 1990-1995 | 0.05 (-0.14, 0.23) | 0.605 |
|  | 1995-2004 | 0.85 (0.76, 0.94) | <0.001* |
|  | 2004-2010 | 0.22 (0.03, 0.40) | 0.023* |
|  | 2010-2019 | 1.32 (1.24, 1.39) | <0.001* |
| **Prevalence** |  |  |  |
| *Global* | 1990-1994 | 1.23 (0.69, 1.77) | <0.001* |
|  | 1994-1997 | -0.50 (-2.17, 1.20) | 0.544 |
|  | 1997-2010 | 1.94 (1.83, 2.04) | <0.001* |
|  | 2010-2019 | -0.31 (-0.46, -0.16) | <0.001* |
| *High SDI* | 1990-1998 | 0.95 (0.75, 1.15) | <0.001* |
|  | 1998-2002 | 2.08 (1.16, 3.01) | <0.001* |
|  | 2002-2006 | 1.21 (0.30, 2.13) | 0.013* |
|  | 2006-2011 | 2.57 (1.99, 3.16) | <0.001* |
|  | 2011-2016 | 1.64 (1.06, 2.22) | <0.001* |
|  | 2016-2019 | -0.91 (-1.8, 0.00) | 0.049* |
| *High-middle SDI* | 1990-1994 | 2.00 (0.95, 3.06) | 0.001* |
|  | 1994-1997 | -1.8 (-4.96, 1.45) | 0.250 |
| **Location** | **Segment** | **APC (%)** | **P-Value** |
| **Prevalence** |  |  |  |
|  | 1997-2001 | 1.91 (0.26, 3.58) | 0.026* |
|  | 2001-2004 | 3.64 (0.31, 7.08) | 0.034* |
|  | 2004-2010 | 1.86 (1.12, 2.61) | <0.001* |
|  | 2010-2019 | -0.40 (-0.69, -0.10) | 0.013* |
| *Middle SDI* | 1990-1994 | 1.69 (1.09, 2.3) | <0.001* |
|  | 1994-2002 | 3.17 (2.91, 3.43) | <0.001* |
|  | 2002-2010 | 3.99 (3.73, 4.25) | <0.001* |
|  | 2010-2016 | -3.14 (-3.55, -2.73) | <0.001* |
|  | 2016-2019 | 2.56 (1.60, 3.53) | <0.001* |
| *Low-middle SDI* | 1990-1992 | 0.76 (-0.60, 2.14) | 0.254 |
|  | 1992-1997 | 2.11 (1.67, 2.55) | <0.001* |
|  | 1997-2004 | 1.40 (1.17, 1.64) | <0.001* |
|  | 2004-2015 | 1.85 (1.74, 1.96) | <0.001* |
|  | 2015-2019 | 2.83 (2.39, 3.27) | <0.001* |
| *Low SDI* | 1990-1995 | 0.29 (0.04, 0.54) | 0.026* |
|  | 1995-2005 | 1.92 (1.81, 2.02) | <0.001* |
|  | 2005-2010 | 1.38 (1.03, 1.74) | <0.001* |
|  | 2010-2019 | 2.62 (2.51, 2.72) | <0.001* |
| **Deaths** |  |  |  |
| *Global* | 1990-1994 | -0.25 (-0.66, 0.17) | 0.219 |
|  | 1994-1997 | -1.10 (-2.39, 0.20) | 0.090* |
|  | 1997-2003 | -0.13 (-0.42, 0.17) | 0.365 |
|  | 2003-2011 | -1.07 (-1.25, -0.90) | <0.001* |
|  | 2011-2014 | -1.78 (-3.05, -0.48) | 0.011* |
|  | 2014-2019 | -1.05 (-1.34, -0.76) | <0.001* |
| *High SDI* | 1990-1997 | -1.2 (-1.33, -1.07) | <0.001* |
|  | 1997-2002 | 0.44 (0.13, 0.75) | 0.008* |
|  | 2002-2007 | -0.52 (-0.82, -0.21) | 0.003* |
|  | 2007-2016 | 0.39 (0.29, 0.50) | <0.001* |
|  | 2016-2019 | -0.82 (-1.31, -0.34) | 0.002* |
| *High-middle SDI* | 1990-1994 | 1.26 (0.50, 2.02) | 0.003* |
|  | 1994-1998 | -2.25 (-3.41, -1.08) | 0.001* |
|  | 1998-2003 | -0.25 (-0.99, 0.50) | 0.498 |
|  | 2003-2019 | -1.66 (-1.75, -1.57) | <0.001* |
| *Middle SDI* | 1990-1995 | -0.77 (-0.98, -0.55) | <0.001* |
|  | 1995-2004 | 0.22 (0.12, 0.33) | <0.001* |
|  | 2004-2010 | -0.76 (-0.98, -0.55) | <0.001* |
|  | 2010-2016 | -3.93 (-4.13, -3.72) | <0.001* |
|  | 2016-2019 | -0.43 (-0.92, 0.05) | 0.078 |
| *Low-middle SDI* | 1990-1995 | -0.23 (-0.55, 0.09) | 0.154 |
| **Location** | **Segment** | **APC (%)** | **P-Value** |
| **Deaths** | 1995-2001 | 0.46 (0.14, 0.78) | 0.007* |
|  | 2001-2012 | -1.15 (-1.26, -1.04) | <0.001* |
|  | 2012-2019 | 0.21 (0.02, 0.40) | 0.032* |
| *Low SDI* | 1990-1995 | -0.19 (-0.36, -0.02) | 0.033* |
|  | 1995-2002 | 0.32 (0.19, 0.45) | <0.001* |
|  | 2002-2010 | -0.43 (-0.53, -0.32) | <0.001* |
|  | 2010-2019 | 0.32 (0.25, 0.39) | <0.001* |
| **DALYs** |  |  |  |
| *Global* | 1990-1994 | -0.12 (-0.56, 0.33) | 0.576 |
|  | 1994-1997 | -1.12 (-2.49, 0.27) | 0.106 |
|  | 1997-2003 | -0.18 (-0.49, 0.13) | 0.234 |
|  | 2003-2011 | -0.94 (-1.13, -0.76) | <0.001* |
|  | 2011-2014 | -2.08 (-3.44, -0.71) | 0.006* |
|  | 2014-2019 | -1.06 (-1.37, -0.75) | <0.001* |
| *High SDI* | 1990-1997 | -1.17 (-1.28, -1.06) | <0.001* |
|  | 1997-2002 | 0.46 (0.20, 0.73) | 0.002* |
|  | 2002-2007 | 0.01 (-0.26, 0.27) | 0.958 |
|  | 2007-2016 | 0.81 (0.71, 0.90) | <0.001* |
|  | 2016-2019 | -1.05 (-1.46, -0.63) | <0.001* |
| *High-middle SDI* | 1990-1994 | 1.45 (0.56, 2.34) | 0.003* |
|  | 1994-1998 | -2.42 (-3.76, -1.06) | 0.002* |
|  | 1998-2004 | -0.30 (-0.92, 0.31) | 0.315 |
|  | 2004-2019 | -1.85 (-1.96, -1.74) | <0.001* |
| *Middle SDI* | 1990-1995 | -0.89 (-1.16, -0.63) | <0.001* |
|  | 1995-1999 | 0.55 (-0.06, 1.16) | 0.073 |
|  | 1999-2010 | -0.38 (-0.47, -0.28) | <0.001* |
|  | 2010-2015 | -4.63 (-4.99, -4.27) | <0.001* |
|  | 2015-2019 | -1.18 (-1.56, -0.80) | <0.001* |
| *Low-middle SDI* | 1990-1995 | -0.12 (-0.42, 0.18) | 0.408 |
|  | 1995-1998 | 1.07 (-0.29, 2.45) | 0.115 |
|  | 1998-2002 | -0.32 (-0.99, 0.36) | 0.331 |
|  | 2002-2012 | -1.19 (-1.31, -1.07) | <0.001* |
|  | 2012-2019 | 0.24 (0.06, 0.43) | 0.012* |
| *Low SDI* | 1990-1995 | -0.14 (-0.27, -0.02) | 0.029* |
|  | 1995-1998 | 0.42 (-0.15, 0.99) | 0.139 |
|  | 1998-2003 | 0.01 (-0.17, 0.19) | 0.884 |
|  | 2003-2010 | -0.58 (-0.68, -0.49) | <0.001* |
|  | 2010-2014 | 0.47 (0.18, 0.76) | 0.003* |
|  | 2014-2019 | 0.23 (0.10, 0.36) | 0.002* |
| **YLDs** |  |  |  |
| *Global* | 1990-1994 | 1.17 (0.61, 1.73) | <0.001* |
| **Location** | **Segment** | **APC (%)** | **P-Value** |
| **YLDs** | 1994-1997 | -0.54 (-2.27, 1.22) | 0.524 |
|  | 1997-2010 | 1.61 (1.5, 1.71) | <0.001* |
|  | 2010-2019 | -0.43 (-0.59, -0.27) | <0.001* |
| *High SDI* | 1990-1998 | 0.82 (0.61, 1.03) | <0.001* |
|  | 1998-2002 | 1.91 (0.96, 2.87) | 0.001* |
|  | 2002-2006 | 0.97 (0.03, 1.92) | 0.044* |
|  | 2006-2011 | 2.34 (1.73, 2.94) | <0.001* |
|  | 2011-2016 | 1.53 (0.93, 2.13) | <0.001* |
|  | 2016-2019 | -0.93 (-1.86, 0.00) | 0.050 |
| *High-middle SDI* | 1990-1994 | 2.15 (0.96, 3.36) | 0.001* |
|  | 1994-1997 | -2.15 (-5.72, 1.56) | 0.237 |
|  | 1997-2009 | 2.19 (1.94, 2.45) | <0.001* |
|  | 2009-2019 | -0.42 (-0.71, -0.13) | 0.007* |
| *Middle SDI* | 1990-1994 | 1.14 (0.6, 1.68) | <0.001* |
|  | 1994-2002 | 2.57 (2.34, 2.80) | <0.001* |
|  | 2002-2010 | 3.31 (3.08, 3.55) | <0.001* |
|  | 2010-2016 | -3.09 (-3.45, -2.72) | <0.001* |
|  | 2016-2019 | 2.13 (1.27, 3.00) | <0.001* |
| *Low-middle SDI* | 1990-2001 | 1.29 (1.18, 1.40) | <0.001* |
|  | 2001-2004 | 0.50 (-1.13, 2.15) | 0.531 |
|  | 2004-2014 | 1.15 (1.00, 1.31) | <0.001* |
|  | 2014-2019 | 2.25 (1.88, 2.63) | <0.001* |
| *Low SDI* | 1990-1995 | 0.17 (0.00, 0.35) | 0.048* |
|  | 1995-2005 | 1.15 (1.08, 1.22) | <0.001* |
|  | 2005-2009 | 0.42 (0.04, 0.81) | 0.033* |
|  | 2009-2017 | 1.63 (1.53, 1.74) | <0.001* |
|  | 2017-2019 | 2.32 (1.54, 3.11) | <0.001* |
| **YLLs** |  |  |  |
| *Global* | 1990-1994 | -0.20 (-0.63, 0.23) | 0.326 |
|  | 1994-1997 | -1.15 (-2.49, 0.20) | 0.088 |
|  | 1997-2003 | -0.31 (-0.62, -0.01) | 0.044* |
|  | 2003-2011 | -1.15 (-1.33, -0.97) | <0.001* |
|  | 2011-2014 | -2.22 (-3.54, -0.88) | 0.003* |
|  | 2014-2019 | -1.12 (-1.42, -0.82) | <0.001* |
| *High SDI* | 1990-1997 | -1.42 (-1.53, -1.31) | <0.001* |
|  | 1997-2002 | 0.30 (0.03, 0.57) | 0.033* |
|  | 2002-2007 | -0.21 (-0.48, 0.06) | 0.119 |
|  | 2007-2016 | 0.61 (0.52, 0.71) | <0.001* |
|  | 2016-2019 | -1.01 (-1.43, -0.59) | <0.001* |
| *High-middle SDI* | 1990-1994 | 1.43 (0.55, 2.32) | 0.003* |
|  | 1994-1998 | -2.57 (-3.9, -1.22) | 0.001* |
| **Location** | **Segment** | **APC (%)** | **P-Value** |
|  | 1998-2003 | -0.34 (-1.20, 0.53) | 0.424 |
|  | 2003-2019 | -2.05 (-2.15, -1.95) | <0.001* |
| *Middle SDI* | 1990-1995 | -0.98 (-1.25, -0.70) | <0.001* |
|  | 1995-1999 | 0.49 (-0.13, 1.11) | 0.114 |
|  | 1999-2010 | -0.55 (-0.65, -0.45) | <0.001* |
|  | 2010-2015 | -4.72 (-5.1, -4.35) | <0.001* |
|  | 2015-2019 | -1.31 (-1.69, -0.92) | <0.001* |
| *Low-middle SDI* | 1990-1995 | -0.15 (-0.46, 0.16) | 0.313 |
|  | 1995-1998 | 1.05 (-0.34, 2.46) | 0.129 |
|  | 1998-2002 | -0.35 (-1.04, 0.34) | 0.299 |
|  | 2002-2012 | -1.26 (-1.38, -1.13) | <0.001* |
|  | 2012-2019 | 0.18 (-0.01, 0.36) | 0.061 |
| *Low SDI* | 1990-1995 | -0.15 (-0.28, -0.02) | 0.023* |
|  | 1995-1998 | 0.40 (-0.17, 0.97) | 0.150 |
|  | 1998-2003 | -0.01 (-0.19, 0.17) | 0.875 |
|  | 2003-2010 | -0.61 (-0.71, -0.52) | <0.001* |
|  | 2010-2014 | 0.44 (0.16, 0.72) | 0.005* |
|  | 2014-2019 | 0.19 (0.06, 0.32) | 0.007* |

APC: annual percentage change; DALYs: disability-adjusted life-years; YLDs: years lived with disability; YLLs: years of life lost; SDI: socio-demographic index; *: P<0.05.

Table S3 Disease burden of uterine cancer in different age groups, 1990 and 2019

| **Rate per 100,000 population (95%UI)** | | | | | |
| --- | --- | --- | --- | --- | --- |
| **Age** | | **Incidence** | | **Prevalence** | |
|  | | **1990** | **2019** | **1990** | **2019** |
| 20 to24 | 0.38 (0.25, 0.45) | | 0.38 (0.26, 0.45) | 3.08 (2.04, 3.63) | 3.24 (2.24, 3.81) |
| 25 to 29 | | 0.81 (0.59, 0.93) | 0.9 (0.68, 1.04) | 6.51 (4.82, 7.45) | 7.61 (5.81, 8.83) |
| 30 to 34 | | 1.62 (1.28, 1.83) | 1.94 (1.53, 2.23) | 12.96 (10.41, 14.49) | 16.26 (12.85, 18.74) |
| 35 to 39 | | 3.2 (2.62, 3.56) | 3.65 (3.03, 4.14) | 25.18 (21.02, 27.84) | 30.54 (25.31, 34.58) |
| 40 to 44 | | 5.2 (4.47, 5.69) | 6.18 (5.29, 6.94) | 39.72 (34.79, 43.16) | 50.66 (43.52, 56.83) |
| 45 to 49 | | 10.2 (8.9, 10.97) | 12.34 (10.95, 13.81) | 78.15 (69.36, 83.57) | 101.41 (90.23, 113.56) |
| 50 to 54 | | 20.86 (18.94, 22.32) | 24.31 (21.69, 27.38) | 161.47 (148.7, 171.78) | 199.96 (177.94, 225.67) |
| 55 to 59 | | 28.91 (26.72, 30.77) | 35.71 (32.27, 39.84) | 217.65 (203.31, 229.63) | 289.29 (261.44, 322.3) |
| 60 to 64 | | 37.78 (36.01, 39.61) | 45.09 (40.9, 49.64) | 268.28 (257.45, 279.8) | 351.08 (318, 386.58) |
| 65 to 69 | | 42.73 (40.87, 44.9) | 47.56 (43.48, 52.4) | 283.59 (273.5, 295.04) | 352.12 (321.51, 386.74) |
| 70 to 74 | | 42.7 (40.63, 44.8) | 48.69 (44.31, 53.91) | 254.24 (243.14, 263.72) | 337.02 (305.04, 373.21) |
| 75 to 79 | | 44.07 (41.36, 46.04) | 45.17 (40.34, 50.33) | 229.15 (215.36, 239.24) | 280.39 (249.45, 311.48) |
| 80 to 84 | | 38.38 (34.36, 40.76) | 41 (34.41, 46.36) | 152.77 (136.13, 162.5) | 208.72 (173.58, 235.29) |
| 85 to 89 | | 34.36 (29.3, 37.1) | 36.55 (28.89, 41.63) | 94.6 (79.96, 102.67) | 136.11 (106.3, 157.16) |
| 90 to 94 | | 27.92 (22.43, 30.77) | 30.4 (23.17, 34.92) | 36.05 (28.57, 40.02) | 56.34 (42.87, 65.24) |
| 95 plus | | 26.4 (20.23, 29.64) | 30.63 (22.54, 35.41) | 13.94 (10.69, 15.66) | 17.2 (12.63, 19.89) |

|  |
| --- |

Table S3 (continued)

| **Rate per 100,000 population (95%UI)** | | | | | |
| --- | --- | --- | --- | --- | --- |
| **Age** | | **Deaths** | | **DALYs** | |
|  | | **1990** | **2019** | **1990** | **2019** |
| 20 to24 | 0.06 (0.04, 0.08) | | 0.03 (0.02, 0.04) | 4.33 (2.52, 5.41) | 2.47 (1.68, 2.85) |
| 25 to 29 | | 0.13 (0.08, 0.16) | 0.08 (0.06, 0.09) | 8.47 (5.44, 10.21) | 5.35 (3.95, 6.14) |
| 30 to 34 | | 0.26 (0.18, 0.31) | 0.17 (0.13, 0.19) | 15.73 (11.04, 18.64) | 10.67 (7.99, 12.18) |
| 35 to 39 | | 0.58 (0.42, 0.69) | 0.36 (0.28, 0.4) | 31.65 (23.09, 37.27) | 20.48 (16.05, 23.04) |
| 40 to 44 | | 1.1 (0.86, 1.27) | 0.76 (0.61, 0.86) | 54.2 (42.04, 62.5) | 39.01 (31.33, 43.73) |
| 45 to 49 | | 2.09 (1.65, 2.37) | 1.46 (1.23, 1.64) | 92.93 (73.9, 104.74) | 67.75 (57.1, 76.03) |
| 50 to 54 | | 3.91 (3.31, 4.36) | 2.7 (2.32, 3.02) | 155.65 (131.48, 173.06) | 112.89 (97.96, 126.05) |
| 55 to 59 | | 6.11 (5.25, 6.75) | 4.52 (3.99, 5.08) | 212.86 (182.06, 235.17) | 165.15 (146.54, 184.87) |
| 60 to 64 | | 9.99 (9.12, 10.87) | 7.84 (7.03, 8.61) | 296.46 (270.16, 323.21) | 241.44 (217.17, 267.98) |
| 65 to 69 | | 13.43 (12.38, 14.67) | 10.62 (9.72, 11.87) | 334.28 (308.23, 364.13) | 272.03 (247.9, 306.65) |
| 70 to 74 | | 16.33 (15.13, 17.83) | 13.33 (12.25, 15.08) | 331.35 (307.47, 361.97) | 278.31 (255.51, 314.02) |
| 75 to 79 | | 19.89 (18.39, 21.32) | 15.45 (13.93, 17.48) | 319.72 (296.18, 342.19) | 254.17 (229.18, 286.26) |
| 80 to 84 | | 22.29 (20.08, 23.97) | 18.72 (16.03, 21.38) | 273.76 (246.41, 294.65) | 234.24 (199.73, 265.11) |
| 85 to 89 | | 24.52 (21.06, 26.47) | 21.41 (17.26, 24.55) | 228.95 (196.67, 247.72) | 202.84 (163.42, 231.7) |
| 90 to 94 | | 28.04 (22.81, 30.94) | 26.16 (20.07, 29.73) | 199.49 (162.54, 220.74) | 187.35 (143.48, 213.83) |
| 95 plus | | 32.73 (24.98, 36.83) | 32.78 (24.45, 37.66) | 175.69 (133.54, 197.94) | 173.95 (130.46, 199.58) |

Table S3 (continued)

| **Rate per 100,000 population (95%UI)** | | | | | |
| --- | --- | --- | --- | --- | --- |
| **Age** | | **YLDs** | | **YLLs** | |
|  | | **1990** | **2019** | **1990** | **2019** |
| 20 to24 | 0.21 (0.12, 0.3) | | 0.21 (0.12, 0.31) | 4.13 (2.37, 5.18) | 2.26 (1.54, 2.59) |
| 25 to 29 | | 0.43 (0.27, 0.61) | 0.49 (0.31, 0.7) | 8.05 (5.09, 9.73) | 4.86 (3.59, 5.57) |
| 30 to 34 | | 0.85 (0.55, 1.19) | 1.04 (0.67, 1.46) | 14.89 (10.38, 17.7) | 9.63 (7.19, 11.02) |
| 35 to 39 | | 1.66 (1.09, 2.31) | 1.97 (1.3, 2.76) | 29.99 (21.77, 35.42) | 18.51 (14.46, 20.72) |
| 40 to 44 | | 2.66 (1.78, 3.69) | 3.28 (2.19, 4.54) | 51.54 (40, 59.54) | 35.73 (28.51, 40.03) |
| 45 to 49 | | 5.22 (3.63, 7.12) | 6.54 (4.45, 9.18) | 87.71 (69.23, 99.08) | 61.21 (51.58, 68.71) |
| 50 to 54 | | 10.62 (7.45, 14.29) | 12.77 (8.72, 17.7) | 145.03 (123, 161.98) | 100.11 (86.18, 112.11) |
| 55 to 59 | | 14.52 (10.2, 19.54) | 18.56 (12.9, 25.17) | 198.34 (170.4, 219.08) | 146.59 (129.39, 164.73) |
| 60 to 64 | | 18.57 (13.39, 24.54) | 23.2 (16.15, 31.27) | 277.89 (253.84, 302.42) | 218.24 (195.62, 239.66) |
| 65 to 69 | | 20.59 (14.92, 26.93) | 24.16 (16.82, 32) | 313.69 (289.07, 342.63) | 247.87 (226.96, 276.96) |
| 70 to 74 | | 19.71 (14.59, 25.44) | 24.04 (17.39, 31.63) | 311.64 (288.65, 340.25) | 254.27 (233.66, 287.64) |
| 75 to 79 | | 19.56 (14.53, 25.2) | 21.57 (15.64, 28.22) | 300.15 (277.43, 321.83) | 232.6 (209.64, 263.09) |
| 80 to 84 | | 15.32 (11.3, 19.67) | 18.07 (12.89, 23.31) | 258.44 (232.82, 277.89) | 216.17 (185.12, 247) |
| 85 to 89 | | 12.06 (8.53, 15.63) | 14.45 (10, 18.73) | 216.89 (186.33, 234.15) | 188.4 (151.96, 216.05) |
| 90 to 94 | | 7.35 (5.02, 9.7) | 9.08 (6, 12.05) | 192.14 (156.32, 212) | 178.27 (136.79, 202.67) |
| 95 plus | | 5.26 (3.42, 7.2) | 6.32 (4.02, 8.71) | 170.43 (130.19, 191.87) | 167.64 (125.21, 192.54) |

Table S4 AAPCs in 204 countries and territories, 1990-2019

| **location** | **Incidence** | | **Prevalence** | | **Deaths** | | **DALYs** | | **YLDs** | | **YLLs** | |
| --- | --- | --- | --- | --- | --- | --- | --- | --- | --- | --- | --- | --- |
|  | **AAPC（%） (95% CI)** | **P-Value** | **AAPC （%） (95% CI)** | **P-Value** | **AAPC（%） (95% CI)** | **P-Value** | **AAPC（%） (95% CI)** | **P-Value** | **AAPC（%） (95% CI)** | **P-Value** | **AAPC（%） (95% CI)** | **P-Value** |
| Republic of Korea | -1.37 (-1.75, -0.99) | <0.001* | -0.58 (-0.95, -0.2) | 0.002* | -4.24 (-4.53, -3.94) | <0.001* | -4.24 (-4.52, -3.96) | <0.001* | -0.94 (-1.34, -0.55) | <0.001* | -4.5 (-4.77, -4.23) | <0.001* |
| Turkmenistan | -2.02 (-2.53, -1.5) | <0.001* | -1.3 (-2.02, -0.56) | 0.001* | -3.43 (-4.26, -2.59) | <0.001* | -3.3 (-4.33, -2.25) | <0.001* | -1.63 (-2.24, -1.01) | <0.001* | -3.34 (-4.29, -2.39) | <0.001* |
| Jamaica | 3.48 (2.8, 4.16) | <0.001* | 3.67 (2.94, 4.4) | <0.001* | 3.07 (2.11, 4.04) | <0.001* | 3.05 (2.14, 3.96) | <0.001* | 3.49 (2.79, 4.2) | <0.001* | 3.03 (2.1, 3.96) | <0.001* |
| China | 0.77 (0.56, 0.97) | <0.001* | 1.85 (1.62, 2.09) | <0.001* | -2.41 (-2.56, -2.27) | <0.001* | -2.34 (-2.49, -2.19) | <0.001* | 1.33 (1.11, 1.55) | <0.001* | -2.56 (-2.71, -2.41) | <0.001* |
| Maldives | 0.06 (-0.32, 0.44) | 0.748 | 1.02 (0.54, 1.5) | <0.001* | -2.27 (-2.65, -1.88) | <0.001* | -2.6 (-2.94, -2.26) | <0.001* | 0.55 (0.16, 0.94) | 0.006* | -2.77 (-3.1, -2.43) | <0.001* |
| Lesotho | 2.46 (2, 2.92) | <0.001* | 2.69 (2.26, 3.12) | <0.001* | 2.2 (1.77, 2.63) | <0.001* | 2.22 (1.72, 2.72) | <0.001* | 2.43 (2.02, 2.84) | <0.001* | 2.21 (1.72, 2.71) | <0.001* |
| Italy | 4.04 (3.51, 4.58) | <0.001* | 4.25 (3.71, 4.79) | <0.001* | 2.04 (1.67, 2.41) | <0.001* | 2.42 (2, 2.84) | <0.001* | 4.08 (3.54, 4.62) | <0.001* | 2.03 (1.7, 2.37) | <0.001* |
| Taiwan (Province of China) | 4.88 (3.92, 5.84) | <0.001* | 5.41 (4.66, 6.16) | <0.001* | 1.95 (1.36, 2.55) | <0.001* | 2.85 (2.05, 3.66) | <0.001* | 4.97 (4, 5.95) | <0.001* | 2.6 (1.96, 3.24) | <0.001* |
| Antigua and Barbuda | 2.38 (2.17, 2.59) | <0.001* | 2.53 (2.34, 2.73) | <0.001* | 1.94 (1.66, 2.23) | <0.001* | 1.67 (1.48, 1.86) | <0.001* | 2.42 (2.24, 2.59) | <0.001* | 1.63 (1.44, 1.82) | <0.001* |
| Kazakhstan | -0.2 (-0.62, 0.23) | 0.361 | 0.38 (-0.11, 0.87) | 0.128 | -1.78 (-2.69, -0.86) | <0.001* | -1.81 (-2.76, -0.85) | <0.001* | 0.16 (-0.29, 0.61) | 0.49 | -1.93 (-2.88, -0.96) | <0.001* |
| Luxembourg | -0.24 (-0.8, 0.32) | 0.399 | 0.02 (-0.6, 0.63) | 0.961 | -1.68 (-2.01, -1.36) | <0.001* | -1.66 (-2, -1.31) | <0.001* | -0.13 (-0.72, 0.47) | 0.676 | -1.87 (-2.2, -1.55) | <0.001* |
| Iceland | -0.52 (-0.84, -0.21) | 0.001* | -0.42 (-0.68, -0.16) | 0.002* | -1.56 (-1.74, -1.37) | <0.001* | -1.54 (-1.74, -1.35) | <0.001* | -0.44 (-0.72, -0.17) | 0.002* | -1.73 (-1.9, -1.55) | <0.001* |
| Hungary | 0.29 (-0.24, 0.81) | 0.284 | 0.76 (0.24, 1.28) | 0.004* | -1.53 (-2.18, -0.87) | <0.001* | -1.49 (-2.23, -0.73) | <0.001* | 0.54 (0.01, 1.08) | 0.047* | -1.67 (-2.41, -0.92) | <0.001* |
| Austria | -0.37 (-1.09, 0.36) | 0.325 | -0.18 (-0.93, 0.57) | 0.635 | -1.53 (-1.85, -1.2) | <0.001* | -1.62 (-2, -1.24) | <0.001* | -0.28 (-1.01, 0.45) | 0.447 | -1.83 (-2.19, -1.47) | <0.001* |
| Uganda | 2.12 (1.78, 2.46) | <0.001* | 3.15 (2.63, 3.68) | <0.001* | 1.53 (1.29, 1.77) | <0.001* | 1.5 (1.24, 1.76) | <0.001* | 2.34 (1.91, 2.77) | <0.001* | 1.48 (1.22, 1.74) | <0.001* |
| Nicaragua | 3.13 (2.22, 4.04) | <0.001* | 3.85 (3.08, 4.64) | <0.001* | 1.52 (0.57, 2.48) | 0.002* | 1.38 (0.63, 2.15) | <0.001* | 3.54 (2.7, 4.38) | <0.001* | 1.26 (0.51, 2.02) | 0.001* |
| Honduras | 2.54 (1.92, 3.17) | <0.001* | 3.4 (3.01, 3.79) | <0.001* | 1.51 (0.84, 2.19) | <0.001* | 1.26 (0.56, 1.97) | <0.001* | 2.9 (2.48, 3.32) | <0.001* | 1.2 (0.5, 1.91) | 0.001* |
| C么te d'Ivoire | 0.22 (0.03, 0.4) | 0.022* | 0.61 (0.42, 0.81) | <0.001* | -1.5 (-1.64, -1.36) | <0.001* | -1.55 (-1.63, -1.47) | <0.001* | 0.38 (0.19, 0.57) | <0.001* | -1.74 (-1.81, -1.66) | <0.001* |
| Grenada | 2.13 (1.8, 2.45) | <0.001* | 2.33 (1.7, 2.97) | <0.001* | 1.49 (0.82, 2.16) | <0.001* | 1.27 (0.75, 1.81) | <0.001* | 2.17 (1.51, 2.82) | <0.001* | 1.24 (0.71, 1.77) | <0.001* |
| American Samoa | 1.86 (1.1, 2.63) | <0.001* | 2.04 (1.21, 2.88) | <0.001* | 1.43 (1.03, 1.83) | <0.001* | 1.54 (1.08, 2.01) | <0.001* | 1.92 (1.12, 2.72) | <0.001* | 1.52 (1.07, 1.97) | <0.001* |
| Panama | 3.13 (2.66, 3.59) | <0.001* | 3.93 (3.5, 4.36) | <0.001* | 1.41 (1.03, 1.8) | <0.001* | 1.5 (1.12, 1.89) | <0.001* | 3.45 (3.05, 3.86) | <0.001* | 1.39 (1.01, 1.78) | <0.001* |
| Belarus | 0.43 (-0.05, 0.91) | 0.079 | 0.77 (0.29, 1.25) | 0.002* | -1.35 (-2.16, -0.54) | 0.001* | -1.32 (-2.17, -0.47) | 0.003* | 0.63 (0.14, 1.11) | 0.011* | -1.53 (-2.4, -0.66) | 0.001* |
| Tajikistan | 1.92 (1.24, 2.61) | <0.001* | 2.31 (1.36, 3.26) | <0.001* | 1.33 (0.93, 1.73) | <0.001* | 1.2 (0.81, 1.58) | <0.001* | 2.08 (1.22, 2.94) | <0.001* | 1.16 (0.78, 1.55) | <0.001* |
| Ecuador | 0.42 (-0.6, 1.46) | 0.418 | 1.59 (0.39, 2.82) | 0.010* | -1.31 (-2.05, -0.57) | 0.001* | -1.39 (-2.13, -0.65) | <0.001* | 1 (-0.08, 2.09) | 0.069 | -1.49 (-2.22, -0.75) | <0.001* |
| Zimbabwe | 1.25 (0.43, 2.08) | 0.003* | 1.25 (0.71, 1.79) | <0.001* | 1.3 (0.64, 1.95) | <0.001* | 1.43 (0.71, 2.16) | <0.001* | 1.24 (0.24, 2.26) | 0.015* | 1.44 (0.72, 2.17) | <0.001* |
| Algeria | 0.52 (0.36, 0.68) | <0.001* | 1.53 (1.38, 1.67) | <0.001* | -1.29 (-1.38, -1.2) | <0.001* | -1.16 (-1.24, -1.07) | <0.001* | 1.02 (0.87, 1.17) | <0.001* | -1.27 (-1.35, -1.18) | <0.001* |
| Bermuda | 0.33 (-0.08, 0.75) | 0.118 | 0.79 (0.57, 1.02) | <0.001* | -1.27 (-1.46, -1.07) | <0.001* | -1.29 (-1.63, -0.95) | <0.001* | 0.53 (0.11, 0.96) | 0.014* | -1.43 (-1.71, -1.14) | <0.001* |
| Barbados | 1.88 (1.47, 2.3) | <0.001* | 2.13 (1.66, 2.61) | <0.001* | 1.26 (0.98, 1.54) | <0.001* | 1.16 (0.86, 1.46) | <0.001* | 1.99 (1.53, 2.45) | <0.001* | 1.11 (0.81, 1.42) | <0.001* |
| Northern Mariana Islands | 1.58 (0.75, 2.41) | <0.001* | 1.67 (0.82, 2.53) | <0.001* | 1.19 (0.78, 1.6) | <0.001* | 1.19 (0.75, 1.64) | <0.001* | 1.61 (0.74, 2.48) | <0.001* | 1.17 (0.73, 1.61) | <0.001* |
| Bangladesh | 0.25 (0, 0.49) | 0.051 | 1.84 (1.55, 2.13) | <0.001* | -1.17 (-1.52, -0.82) | <0.001* | -1.27 (-1.76, -0.77) | <0.001* | 0.91 (0.64, 1.17) | <0.001* | -1.33 (-1.82, -0.83) | <0.001* |
| Portugal | 0.9 (0.71, 1.09) | <0.001* | 1.26 (1.06, 1.45) | <0.001* | -1.17 (-1.32, -1.01) | <0.001* | -1.14 (-1.26, -1.02) | <0.001* | 1.08 (0.89, 1.26) | <0.001* | -1.39 (-1.67, -1.11) | <0.001* |
| Ethiopia | -0.56 (-0.78, -0.34) | <0.001* | 0.64 (0.35, 0.93) | <0.001* | -1.17 (-1.3, -1.05) | <0.001* | -1.58 (-1.76, -1.4) | <0.001* | -0.18 (-0.44, 0.08) | 0.175 | -1.61 (-1.78, -1.43) | <0.001* |
| Mauritius | -0.35 (-0.78, 0.07) | 0.105 | -0.04 (-0.48, 0.4) | 0.861 | -1.16 (-1.6, -0.72) | <0.001* | -1.09 (-2.04, -0.12) | 0.027* | -0.21 (-0.64, 0.23) | 0.351 | -1.15 (-2.1, -0.18) | 0.020* |
| Pakistan | 1.98 (1.81, 2.15) | <0.001* | 2.91 (2.67, 3.15) | <0.001* | 1.16 (1.06, 1.27) | <0.001* | 1.16 (1.02, 1.31) | <0.001* | 2.36 (2.11, 2.6) | <0.001* | 1.13 (0.99, 1.28) | <0.001* |
| Denmark | 0.04 (-0.43, 0.51) | 0.88 | 0.29 (-0.16, 0.74) | 0.213 | -1.15 (-1.4, -0.89) | <0.001* | -1.34 (-1.59, -1.08) | <0.001* | 0.16 (-0.24, 0.56) | 0.429 | -1.55 (-1.77, -1.32) | <0.001* |
| Costa Rica | 2.63 (1.89, 3.38) | <0.001* | 3.01 (2.55, 3.47) | <0.001* | 1.14 (0.62, 1.67) | <0.001* | 1.24 (0.79, 1.69) | <0.001* | 2.75 (2.01, 3.5) | <0.001* | 1.12 (0.67, 1.57) | <0.001* |
| Qatar | 2.85 (2.22, 3.48) | <0.001* | 3.71 (3.1, 4.31) | <0.001* | 1.12 (0.31, 1.94) | 0.007* | 0.61 (-0.23, 1.45) | 0.156 | 3.1 (2.5, 3.71) | <0.001* | 0.43 (-0.4, 1.27) | 0.311 |
| Singapore | 3.43 (2.55, 4.31) | <0.001* | 4.18 (3.3, 5.06) | <0.001* | 1.1 (0.16, 2.04) | 0.022* | 1.41 (0.56, 2.26) | 0.001* | 3.72 (2.89, 4.55) | <0.001* | 1.22 (0.38, 2.08) | 0.005* |
| Dominican Republic | 2.14 (1.62, 2.66) | <0.001* | 2.84 (2.22, 3.45) | <0.001* | 1.09 (0.56, 1.63) | <0.001* | 1.11 (0.55, 1.67) | <0.001* | 2.38 (1.81, 2.95) | <0.001* | 1.06 (0.49, 1.63) | <0.001* |
| Venezuela (Bolivarian Republic of) | 0.55 (0.29, 0.81) | <0.001* | 1.3 (0.99, 1.61) | <0.001* | -1.08 (-1.28, -0.89) | <0.001* | -1.07 (-1.28, -0.86) | <0.001* | 0.91 (0.63, 1.19) | <0.001* | -1.18 (-1.39, -0.97) | <0.001* |
| Switzerland | 0.05 (-0.21, 0.32) | 0.707 | 0.19 (-0.08, 0.46) | 0.165 | -1.06 (-1.41, -0.7) | <0.001* | -0.96 (-1.38, -0.52) | <0.001* | 0.12 (-0.15, 0.38) | 0.391 | -1.12 (-1.54, -0.71) | <0.001* |
| Cyprus | 1.69 (0.93, 2.46) | <0.001* | 1.88 (1.13, 2.63) | <0.001* | 1.05 (0.73, 1.37) | <0.001* | 0.93 (0.3, 1.56) | 0.004* | 1.78 (1.02, 2.56) | <0.001* | 0.86 (0.23, 1.5) | 0.007* |
| Malta | 0.42 (0.06, 0.78) | 0.021* | 0.68 (0.32, 1.05) | <0.001* | -1.04 (-1.28, -0.79) | <0.001* | -0.94 (-1.11, -0.77) | <0.001* | 0.52 (0.16, 0.89) | 0.005* | -1.11 (-1.31, -0.92) | <0.001* |
| Slovenia | 0.63 (0.45, 0.8) | <0.001* | 0.93 (0.78, 1.09) | <0.001* | -1.04 (-1.24, -0.85) | <0.001* | -1.15 (-1.35, -0.95) | <0.001* | 0.77 (0.59, 0.95) | <0.001* | -1.38 (-1.58, -1.17) | <0.001* |
| Gambia | 1.48 (0.47, 2.49) | 0.004* | 2.12 (1, 3.25) | <0.001* | 1.04 (0.47, 1.62) | <0.001* | 1.13 (0.45, 1.81) | 0.001* | 1.73 (0.68, 2.8) | 0.001* | 1.11 (0.43, 1.79) | 0.001* |
| Thailand | 1 (0.78, 1.22) | <0.001* | 1.66 (1.38, 1.95) | <0.001* | -0.99 (-1.19, -0.79) | <0.001* | -0.83 (-1.02, -0.64) | <0.001* | 1.33 (1.09, 1.57) | <0.001* | -0.97 (-1.17, -0.78) | <0.001* |
| Botswana | 2.07 (1.75, 2.4) | <0.001* | 3.07 (2.72, 3.42) | <0.001* | 0.99 (0.73, 1.26) | <0.001* | 0.95 (0.63, 1.27) | <0.001* | 2.44 (2.04, 2.83) | <0.001* | 0.9 (0.59, 1.21) | <0.001* |
| United Arab Emirates | 0.53 (-0.45, 1.51) | 0.29 | 1.28 (0.32, 2.25) | 0.009* | -0.97 (-2.17, 0.25) | 0.119 | -0.76 (-1.72, 0.22) | 0.129 | 0.9 (0.05, 1.77) | 0.038* | -0.84 (-1.82, 0.15) | 0.096 |
| Jordan | 1.09 (0.48, 1.7) | <0.001* | 1.94 (1.21, 2.67) | <0.001* | -0.96 (-1.47, -0.45) | <0.001* | -1.05 (-1.52, -0.58) | <0.001* | 1.56 (0.93, 2.2) | <0.001* | -1.2 (-1.67, -0.73) | <0.001* |
| Iraq | 2.75 (2.32, 3.18) | <0.001* | 3.75 (3.07, 4.45) | <0.001* | 0.94 (0.57, 1.3) | <0.001* | 1.06 (0.68, 1.45) | <0.001* | 3.18 (2.54, 3.81) | <0.001* | 0.95 (0.58, 1.33) | <0.001* |
| Rwanda | -0.24 (-0.47, 0) | 0.049* | 1.09 (0.69, 1.5) | <0.001* | -0.92 (-1.15, -0.7) | <0.001* | -1.28 (-1.55, -1.01) | <0.001* | 0.17 (-0.1, 0.44) | 0.222 | -1.31 (-1.58, -1.04) | <0.001* |
| South Africa | 1.33 (0.57, 2.1) | 0.001* | 1.57 (0.99, 2.15) | <0.001* | 0.9 (0.24, 1.58) | 0.008* | 0.53 (-0.32, 1.4) | 0.224 | 1.42 (0.61, 2.24) | 0.001* | 0.53 (-0.24, 1.31) | 0.176 |
| Peru | 0.97 (0.04, 1.9) | 0.041* | 2.18 (1.2, 3.17) | <0.001* | -0.89 (-1.74, -0.03) | 0.042* | -0.98 (-1.97, 0.02) | 0.056 | 1.56 (0.59, 2.53) | 0.001* | -1.1 (-2.08, -0.1) | 0.031* |
| Slovakia | 0.93 (0.43, 1.42) | <0.001* | 1.43 (0.91, 1.95) | <0.001* | -0.88 (-1.36, -0.4) | <0.001* | -0.92 (-1.31, -0.52) | <0.001* | 1.17 (0.65, 1.69) | <0.001* | -1.09 (-1.48, -0.7) | <0.001* |
| Saudi Arabia | 4.11 (3.72, 4.5) | <0.001* | 5.27 (4.71, 5.83) | <0.001* | 0.88 (0.66, 1.09) | <0.001* | 1.07 (0.87, 1.26) | <0.001* | 4.63 (4.12, 5.14) | <0.001* | 0.86 (0.69, 1.04) | <0.001* |
| Eritrea | 1.42 (1.33, 1.52) | <0.001* | 2.55 (2.35, 2.75) | <0.001* | 0.87 (0.74, 1.01) | <0.001* | 0.67 (0.58, 0.77) | <0.001* | 1.62 (1.49, 1.74) | <0.001* | 0.65 (0.56, 0.75) | <0.001* |
| Turkey | 1.65 (1.42, 1.89) | <0.001* | 2.82 (2.6, 3.03) | <0.001* | -0.86 (-1.04, -0.67) | <0.001* | -1.02 (-1.33, -0.72) | <0.001* | 2.17 (1.88, 2.46) | <0.001* | -1.2 (-1.51, -0.89) | <0.001* |
| Guatemala | 0.84 (0.01, 1.68) | 0.046* | 2.15 (1.83, 2.48) | <0.001* | -0.85 (-1.44, -0.26) | 0.005* | -0.75 (-1.32, -0.18) | 0.011* | 1.31 (1.01, 1.61) | <0.001* | -0.82 (-1.39, -0.25) | 0.005* |
| Marshall Islands | 1.16 (1.02, 1.3) | <0.001* | 1.36 (1.11, 1.61) | <0.001* | 0.85 (0.8, 0.89) | <0.001* | 0.93 (0.88, 0.98) | <0.001* | 1.22 (1.02, 1.43) | <0.001* | 0.92 (0.88, 0.97) | <0.001* |
| Solomon Islands | 1.37 (0.99, 1.76) | <0.001* | 1.74 (1.48, 2.01) | <0.001* | 0.85 (0.62, 1.08) | <0.001* | 0.85 (0.62, 1.07) | <0.001* | 1.48 (1.19, 1.78) | <0.001* | 0.81 (0.58, 1.05) | <0.001* |
| North Macedonia | 2.77 (2.22, 3.31) | <0.001* | 3.3 (2.87, 3.73) | <0.001* | 0.85 (0.54, 1.15) | <0.001* | 0.77 (0.39, 1.16) | <0.001* | 3.05 (2.66, 3.43) | <0.001* | 0.6 (0.22, 0.99) | 0.002* |
| Sao Tome and Principe | 1.69 (1.54, 1.83) | <0.001* | 2.75 (2.56, 2.95) | <0.001* | 0.84 (0.71, 0.97) | <0.001* | 0.77 (0.6, 0.94) | <0.001* | 2.1 (1.91, 2.3) | <0.001* | 0.74 (0.56, 0.91) | <0.001* |
| Argentina | 0.23 (-0.04, 0.5) | 0.098 | 0.69 (0.41, 0.97) | <0.001* | -0.82 (-1.01, -0.63) | <0.001* | -1 (-1.18, -0.82) | <0.001* | 0.48 (0.24, 0.72) | <0.001* | -1.08 (-1.26, -0.91) | <0.001* |
| Kenya | 1.05 (0.9, 1.2) | <0.001* | 1.84 (1.68, 2.01) | <0.001* | 0.82 (0.69, 0.95) | <0.001* | 0.75 (0.63, 0.87) | <0.001* | 1.36 (1.18, 1.54) | <0.001* | 0.74 (0.62, 0.86) | <0.001* |
| Brazil | 0.6 (0.46, 0.75) | <0.001* | 1.44 (1.29, 1.59) | <0.001* | -0.81 (-0.9, -0.71) | <0.001* | -0.7 (-0.81, -0.6) | <0.001* | 1.03 (0.88, 1.18) | <0.001* | -0.79 (-0.89, -0.69) | <0.001* |
| Georgia | -0.2 (-1.09, 0.7) | 0.663 | 0.22 (-0.97, 1.43) | 0.714 | -0.8 (-2.17, 0.59) | 0.257 | -0.77 (-2.15, 0.64) | 0.283 | -0.14 (-1.05, 0.78) | 0.766 | -0.81 (-2.17, 0.58) | 0.251 |
| Lao People's Democratic Republic | 0.14 (0.06, 0.21) | <0.001* | 1.15 (1.09, 1.21) | <0.001* | -0.8 (-0.87, -0.73) | <0.001* | -0.91 (-0.97, -0.84) | <0.001* | 0.57 (0.5, 0.64) | <0.001* | -0.95 (-1.01, -0.88) | <0.001* |
| Uruguay | 0.28 (0.1, 0.46) | 0.002* | 0.56 (0.37, 0.76) | <0.001* | -0.79 (-1.11, -0.48) | <0.001* | -0.87 (-1.18, -0.56) | <0.001* | 0.49 (0.32, 0.65) | <0.001* | -0.95 (-1.26, -0.64) | <0.001* |
| Egypt | 2.27 (1.98, 2.56) | <0.001* | 3.16 (2.75, 3.56) | <0.001* | 0.79 (0.71, 0.88) | <0.001* | 0.63 (0.46, 0.8) | <0.001* | 2.74 (2.39, 3.09) | <0.001* | 0.48 (0.31, 0.65) | <0.001* |
| Suriname | 1.42 (0.67, 2.17) | <0.001* | 1.85 (1.03, 2.68) | <0.001* | 0.79 (0.24, 1.36) | 0.005* | 0.8 (0.17, 1.44) | 0.013* | 1.52 (0.79, 2.26) | <0.001* | 0.78 (0.15, 1.41) | 0.015* |
| Dominica | 0.57 (0.27, 0.88) | <0.001* | 0.49 (0.02, 0.97) | 0.041* | 0.78 (0.61, 0.95) | <0.001* | 0.59 (0.43, 0.75) | <0.001* | 0.49 (0.09, 0.9) | 0.016* | 0.59 (0.42, 0.75) | <0.001* |
| Saint Vincent and the Grenadines | 1.06 (0.9, 1.22) | <0.001* | 1.21 (0.95, 1.47) | <0.001* | 0.78 (0.53, 1.03) | <0.001* | 0.77 (0.45, 1.09) | <0.001* | 1.08 (0.85, 1.32) | <0.001* | 0.76 (0.43, 1.09) | <0.001* |
| United Kingdom | 1.62 (1.25, 1.99) | <0.001* | 1.81 (1.42, 2.2) | <0.001* | 0.77 (0.58, 0.95) | <0.001* | 0.67 (0.42, 0.93) | <0.001* | 1.68 (1.29, 2.07) | <0.001* | 0.55 (0.28, 0.82) | <0.001* |
| Vanuatu | 1 (0.66, 1.33) | <0.001* | 1.2 (0.84, 1.56) | <0.001* | 0.77 (0.4, 1.14) | <0.001* | 0.93 (0.6, 1.27) | <0.001* | 1.04 (0.69, 1.38) | <0.001* | 0.93 (0.59, 1.26) | <0.001* |
| Paraguay | 0.4 (0.12, 0.69) | 0.006* | 1.07 (0.77, 1.37) | <0.001* | -0.76 (-1.05, -0.46) | <0.001* | -0.81 (-1.11, -0.51) | <0.001* | 0.76 (0.46, 1.05) | <0.001* | -0.88 (-1.19, -0.58) | <0.001* |
| Colombia | 1.48 (1.2, 1.76) | <0.001* | 2.46 (2.1, 2.82) | <0.001* | -0.76 (-0.97, -0.56) | <0.001* | -0.61 (-0.82, -0.39) | <0.001* | 1.94 (1.62, 2.27) | <0.001* | -0.76 (-0.98, -0.54) | <0.001* |
| Papua New Guinea | 1.01 (0.87, 1.15) | <0.001* | 1.28 (1.2, 1.36) | <0.001* | 0.76 (0.7, 0.83) | <0.001* | 0.79 (0.72, 0.86) | <0.001* | 1.08 (0.94, 1.23) | <0.001* | 0.78 (0.71, 0.85) | <0.001* |
| El Salvador | 1.2 (0.92, 1.48) | <0.001* | 2.23 (1.94, 2.51) | <0.001* | -0.75 (-1.02, -0.47) | <0.001* | -0.82 (-1.1, -0.54) | <0.001* | 1.67 (1.4, 1.95) | <0.001* | -0.95 (-1.24, -0.66) | <0.001* |
| Czechia | 2.05 (1.42, 2.67) | <0.001* | 2.46 (1.67, 3.25) | <0.001* | -0.73 (-1.12, -0.35) | <0.001* | -0.42 (-0.73, -0.1) | 0.010* | 2.17 (1.44, 2.9) | <0.001* | -0.74 (-1.04, -0.44) | <0.001* |
| Mauritania | 0.09 (-0.03, 0.21) | 0.16 | 1.11 (0.97, 1.26) | <0.001* | -0.72 (-0.88, -0.57) | <0.001* | -0.96 (-1.13, -0.79) | <0.001* | 0.48 (0.35, 0.6) | <0.001* | -0.99 (-1.16, -0.82) | <0.001* |
| Bosnia and Herzegovina | 2.87 (2.21, 3.53) | <0.001* | 3.52 (2.8, 4.25) | <0.001* | 0.72 (0.18, 1.26) | 0.009* | 0.87 (0.38, 1.35) | <0.001* | 3.12 (2.39, 3.85) | <0.001* | 0.71 (0.2, 1.22) | 0.006* |
| Burundi | -0.36 (-0.7, -0.01) | 0.041* | 0.35 (-0.2, 0.9) | 0.212 | -0.71 (-0.99, -0.43) | <0.001* | -0.89 (-1.19, -0.59) | <0.001* | -0.16 (-0.62, 0.29) | 0.48 | -0.9 (-1.2, -0.61) | <0.001* |
| Myanmar | 0.31 (0.21, 0.4) | <0.001* | 1.14 (1.02, 1.26) | <0.001* | -0.7 (-0.81, -0.58) | <0.001* | -0.87 (-1.02, -0.73) | <0.001* | 0.65 (0.55, 0.75) | <0.001* | -0.92 (-1.06, -0.78) | <0.001* |
| Sierra Leone | 1.24 (1.1, 1.38) | <0.001* | 2 (1.78, 2.22) | <0.001* | 0.7 (0.61, 0.78) | <0.001* | 0.74 (0.62, 0.86) | <0.001* | 1.54 (1.36, 1.72) | <0.001* | 0.72 (0.6, 0.84) | <0.001* |
| Latvia | 2.29 (1.24, 3.36) | <0.001* | 2.57 (1.47, 3.69) | <0.001* | 0.69 (0.1, 1.28) | 0.023* | 0.75 (0.06, 1.46) | 0.034* | 2.43 (1.31, 3.57) | <0.001* | 0.57 (-0.12, 1.26) | 0.106 |
| Lebanon | 2.13 (2, 2.25) | <0.001* | 3.13 (2.98, 3.27) | <0.001* | -0.66 (-0.7, -0.61) | <0.001* | -0.55 (-0.61, -0.48) | <0.001* | 2.6 (2.38, 2.82) | <0.001* | -0.76 (-0.85, -0.67) | <0.001* |
| Guyana | 1.34 (1.03, 1.65) | <0.001* | 1.73 (1.35, 2.1) | <0.001* | 0.66 (0.25, 1.07) | 0.002* | 0.73 (0.36, 1.1) | <0.001* | 1.48 (1.13, 1.83) | <0.001* | 0.71 (0.33, 1.08) | <0.001* |
| Greenland | 0.31 (-0.36, 1) | 0.363 | 0.87 (0.21, 1.55) | 0.010* | -0.65 (-1.33, 0.02) | 0.059 | -0.75 (-1.27, -0.23) | 0.005* | 0.54 (-0.07, 1.14) | 0.082 | -0.82 (-1.34, -0.3) | 0.002* |
| Albania | 2.06 (1.7, 2.42) | <0.001* | 2.7 (2.34, 3.06) | <0.001* | -0.63 (-1.26, 0) | 0.050 | -0.52 (-1.09, 0.04) | 0.069 | 2.36 (2.02, 2.7) | <0.001* | -0.76 (-1.33, -0.18) | 0.010* |
| Germany | 0.2 (-0.14, 0.55) | 0.254 | 0.38 (-0.04, 0.8) | 0.077 | -0.61 (-0.92, -0.31) | <0.001* | -0.63 (-0.87, -0.4) | <0.001* | 0.25 (-0.11, 0.62) | 0.173 | -0.77 (-0.98, -0.56) | <0.001* |
| Equatorial Guinea | 1.97 (1.5, 2.44) | <0.001* | 3.91 (3.21, 4.62) | <0.001* | 0.61 (0.29, 0.92) | <0.001* | 0.23 (-0.11, 0.58) | 0.182 | 2.7 (2.06, 3.33) | <0.001* | 0.17 (-0.17, 0.52) | 0.316 |
| Trinidad and Tobago | 1.27 (0.88, 1.66) | <0.001* | 1.75 (1.32, 2.19) | <0.001* | 0.61 (0.21, 1.01) | 0.003* | 0.64 (0.23, 1.05) | 0.002* | 1.45 (1.01, 1.89) | <0.001* | 0.6 (0.2, 1.01) | 0.004* |
| Guam | 0.03 (-0.58, 0.64) | 0.931 | 0.22 (-0.34, 0.78) | 0.442 | -0.58 (-1.76, 0.62) | 0.344 | -0.16 (-1.17, 0.87) | 0.765 | 0.14 (-0.42, 0.72) | 0.618 | -0.17 (-1.22, 0.89) | 0.746 |
| Chad | 0.82 (0.69, 0.96) | <0.001* | 1.18 (1.1, 1.26) | <0.001* | 0.58 (0.48, 0.69) | <0.001* | 0.52 (0.39, 0.66) | <0.001* | 0.93 (0.85, 1) | <0.001* | 0.52 (0.39, 0.65) | <0.001* |
| Brunei Darussalam | 0.35 (0.18, 0.51) | <0.001* | 0.79 (0.67, 0.9) | <0.001* | -0.55 (-0.71, -0.39) | <0.001* | -0.69 (-0.77, -0.61) | <0.001* | 0.52 (0.39, 0.65) | <0.001* | -0.74 (-0.82, -0.66) | <0.001* |
| Cook Islands | 0.06 (-0.07, 0.19) | 0.364 | 0.24 (0.09, 0.39) | 0.001* | -0.55 (-0.7, -0.41) | <0.001* | -0.64 (-0.78, -0.5) | <0.001* | 0.11 (-0.03, 0.25) | 0.118 | -0.7 (-0.84, -0.55) | <0.001* |
| France | 1.1 (0.88, 1.32) | <0.001* | 1.36 (1.14, 1.58) | <0.001* | -0.54 (-0.69, -0.38) | <0.001* | -0.41 (-0.57, -0.26) | <0.001* | 1.23 (1.01, 1.46) | <0.001* | -0.65 (-0.78, -0.51) | <0.001* |
| Israel | 2.14 (1.57, 2.71) | <0.001* | 2.51 (1.91, 3.1) | <0.001* | 0.54 (0.18, 0.9) | 0.003* | 0.65 (0.1, 1.21) | 0.022* | 2.27 (1.67, 2.87) | <0.001* | 0.45 (-0.06, 0.96) | 0.085 |
| Sri Lanka | 2.26 (1.57, 2.95) | <0.001* | 2.98 (2.26, 3.71) | <0.001* | 0.52 (-0.17, 1.22) | 0.139 | 0.54 (-0.08, 1.17) | 0.087 | 2.55 (1.91, 3.2) | <0.001* | 0.42 (-0.2, 1.04) | 0.187 |
| Uzbekistan | 2.05 (1.55, 2.55) | <0.001* | 2.62 (2, 3.24) | <0.001* | 0.52 (0.12, 0.93) | 0.012* | 0.79 (0.39, 1.2) | <0.001* | 2.28 (1.76, 2.8) | <0.001* | 0.71 (0.31, 1.12) | 0.001* |
| Estonia | 1.74 (0.69, 2.79) | 0.001* | 2.14 (1.1, 3.18) | <0.001* | -0.51 (-1.5, 0.5) | 0.32 | -0.34 (-1.42, 0.74) | 0.534 | 1.9 (0.85, 2.96) | <0.001* | -0.67 (-1.74, 0.42) | 0.229 |
| Sweden | 0.32 (0.19, 0.44) | <0.001* | 0.43 (0.3, 0.55) | <0.001* | -0.51 (-0.73, -0.29) | <0.001* | -0.66 (-0.87, -0.46) | <0.001* | 0.36 (0.24, 0.48) | <0.001* | -0.77 (-1.01, -0.54) | <0.001* |
| Belize | 1.27 (0.65, 1.91) | <0.001* | 1.75 (1.11, 2.4) | <0.001* | 0.51 (0.12, 0.91) | 0.010* | 0.77 (0.44, 1.1) | <0.001* | 1.47 (0.84, 2.11) | <0.001* | 0.74 (0.41, 1.07) | <0.001* |
| Norway | 1.1 (0.87, 1.33) | <0.001* | 1.35 (1.1, 1.59) | <0.001* | -0.5 (-0.94, -0.06) | 0.027* | -0.52 (-1, -0.04) | 0.034* | 1.25 (1.02, 1.48) | <0.001* | -0.77 (-1.22, -0.32) | 0.001* |
| Republic of Moldova | 0.81 (-0.81, 2.45) | 0.331 | 1.17 (-0.46, 2.82) | 0.161 | -0.49 (-1.8, 0.83) | 0.462 | -0.5 (-1.97, 0.99) | 0.51 | 1.01 (-0.62, 2.66) | 0.226 | -0.61 (-2.07, 0.87) | 0.416 |
| Romania | 1.54 (1.33, 1.76) | <0.001* | 2.06 (1.83, 2.29) | <0.001* | -0.49 (-0.88, -0.11) | 0.013* | -0.54 (-0.96, -0.12) | 0.012* | 1.81 (1.59, 2.04) | <0.001* | -0.72 (-1.14, -0.3) | 0.001* |
| Mongolia | 1.05 (0.74, 1.36) | <0.001* | 1.89 (1.63, 2.15) | <0.001* | -0.49 (-0.66, -0.31) | <0.001* | -0.56 (-0.83, -0.29) | <0.001* | 1.48 (1.14, 1.82) | <0.001* | -0.64 (-0.9, -0.37) | <0.001* |
| Micronesia (Federated States of) | 1.26 (1.2, 1.33) | <0.001* | 1.79 (1.68, 1.9) | <0.001* | 0.49 (0.44, 0.54) | <0.001* | 0.49 (0.45, 0.53) | <0.001* | 1.49 (1.38, 1.6) | <0.001* | 0.45 (0.4, 0.51) | <0.001* |
| Morocco | 2.05 (1.96, 2.14) | <0.001* | 3.2 (3.1, 3.31) | <0.001* | 0.49 (0.42, 0.56) | <0.001* | 0.51 (0.46, 0.56) | <0.001* | 2.6 (2.5, 2.7) | <0.001* | 0.43 (0.37, 0.48) | <0.001* |
| Iran (Islamic Republic of) | 2.18 (1.71, 2.65) | <0.001* | 2.86 (2.2, 3.52) | <0.001* | 0.49 (0.33, 0.65) | <0.001* | 0.49 (0.32, 0.66) | <0.001* | 2.56 (1.96, 3.17) | <0.001* | 0.36 (0.14, 0.57) | 0.001* |
| Armenia | 2.07 (1.23, 2.92) | <0.001* | 2.58 (1.72, 3.44) | <0.001* | 0.49 (-0.25, 1.24) | 0.192 | 0.33 (-0.49, 1.15) | 0.432 | 2.31 (1.45, 3.17) | <0.001* | 0.2 (-0.6, 1.02) | 0.623 |
| Cabo Verde | 1.55 (1.3, 1.81) | <0.001* | 2.57 (2.38, 2.77) | <0.001* | 0.48 (0.18, 0.79) | 0.002* | 0.21 (-0.02, 0.44) | 0.07 | 2.04 (1.85, 2.23) | <0.001* | 0.15 (-0.08, 0.38) | 0.205 |
| Malawi | -0.11 (-0.33, 0.11) | 0.309 | 0.51 (0.14, 0.88) | 0.007* | -0.47 (-0.59, -0.35) | <0.001* | -0.66 (-0.79, -0.53) | <0.001* | 0.08 (-0.2, 0.36) | 0.583 | -0.67 (-0.81, -0.54) | <0.001* |
| Serbia | 2.41 (2.04, 2.78) | <0.001* | 2.87 (2.49, 3.25) | <0.001* | 0.46 (-0.04, 0.97) | 0.07 | 0.48 (0.01, 0.95) | 0.047* | 2.66 (2.29, 3.04) | <0.001* | 0.28 (-0.2, 0.77) | 0.249 |
| Bahrain | 1.8 (1.07, 2.53) | <0.001* | 2.73 (1.86, 3.61) | <0.001* | -0.45 (-1.45, 0.56) | 0.381 | -0.54 (-1.54, 0.47) | 0.295 | 2.16 (1.45, 2.88) | <0.001* | -0.63 (-1.67, 0.41) | 0.234 |
| Bahamas | 0.71 (0.31, 1.11) | <0.001* | 0.82 (0.27, 1.38) | 0.004* | 0.45 (0.13, 0.77) | 0.006* | 0.24 (-0.05, 0.53) | 0.102 | 0.74 (0.2, 1.27) | 0.007* | 0.21 (-0.07, 0.5) | 0.146 |
| Yemen | 1.43 (1.14, 1.72) | <0.001* | 2.39 (1.92, 2.85) | <0.001* | 0.44 (0.38, 0.5) | <0.001* | 0.47 (0.38, 0.55) | <0.001* | 1.82 (1.46, 2.18) | <0.001* | 0.42 (0.33, 0.51) | <0.001* |
| Nigeria | 1.01 (0.7, 1.32) | <0.001* | 1.77 (1.37, 2.17) | <0.001* | 0.43 (0.31, 0.56) | <0.001* | 0.31 (0.18, 0.45) | <0.001* | 1.32 (0.95, 1.68) | <0.001* | 0.29 (0.17, 0.41) | <0.001* |
| Kuwait | 1.53 (0.38, 2.7) | 0.009* | 1.96 (0.83, 3.1) | 0.001* | 0.41 (-0.29, 1.11) | 0.255 | 0.16 (-0.62, 0.95) | 0.694 | 1.64 (0.44, 2.84) | 0.007* | 0.01 (-0.77, 0.8) | 0.978 |
| United States of America | 1.32 (0.95, 1.7) | <0.001* | 1.44 (1.05, 1.84) | <0.001* | 0.41 (0.29, 0.52) | <0.001* | 0.62 (0.48, 0.76) | <0.001* | 1.34 (0.96, 1.72) | <0.001* | 0.49 (0.37, 0.61) | <0.001* |
| Palestine | 1.59 (1.27, 1.92) | <0.001* | 2.12 (1.33, 2.91) | <0.001* | 0.41 (0.11, 0.72) | 0.008* | 0.27 (-0.02, 0.55) | 0.064 | 1.78 (1.11, 2.46) | <0.001* | 0.19 (-0.08, 0.47) | 0.17 |
| Eswatini | 0.74 (0.42, 1.05) | <0.001* | 1.22 (0.64, 1.81) | <0.001* | 0.41 (0.05, 0.77) | 0.025* | 0.29 (-0.08, 0.66) | 0.119 | 0.86 (0.47, 1.27) | <0.001* | 0.28 (-0.09, 0.64) | 0.137 |
| Australia | 0.49 (0.07, 0.9) | 0.022* | 0.67 (0.25, 1.08) | 0.002* | -0.4 (-0.52, -0.28) | <0.001* | -0.41 (-0.69, -0.12) | 0.006* | 0.59 (0.16, 1.02) | 0.007* | -0.5 (-0.78, -0.22) | <0.001* |
| Mozambique | 0.97 (0.8, 1.13) | <0.001* | 2 (1.79, 2.21) | <0.001* | 0.4 (0.24, 0.57) | <0.001* | 0.34 (0.16, 0.52) | <0.001* | 1.28 (1.05, 1.5) | <0.001* | 0.3 (0.07, 0.53) | 0.011* |
| Saint Kitts and Nevis | 0.14 (-0.12, 0.39) | 0.292 | 0.33 (0.07, 0.59) | 0.013* | -0.39 (-0.69, -0.09) | 0.010* | -0.7 (-0.9, -0.51) | <0.001* | 0.28 (0.01, 0.55) | 0.044* | -0.76 (-0.95, -0.56) | <0.001* |
| Zambia | 0.38 (0.22, 0.55) | <0.001* | 1.57 (1.4, 1.75) | <0.001* | -0.39 (-0.55, -0.24) | <0.001* | -0.51 (-0.7, -0.32) | <0.001* | 0.79 (0.53, 1.05) | <0.001* | -0.54 (-0.73, -0.36) | <0.001* |
| Fiji | 0.53 (0.24, 0.82) | <0.001* | 0.61 (0.26, 0.96) | 0.001* | 0.38 (0.18, 0.58) | <0.001* | 0.31 (0.1, 0.53) | 0.004* | 0.51 (0.19, 0.83) | 0.002* | 0.31 (0.09, 0.52) | 0.005* |
| Syrian Arab Republic | 1.19 (0.53, 1.86) | <0.001* | 1.88 (1.1, 2.67) | <0.001* | -0.37 (-0.84, 0.1) | 0.119 | -0.62 (-1.14, -0.09) | 0.021* | 1.57 (0.85, 2.3) | <0.001* | -0.74 (-1.27, -0.21) | 0.006* |
| Bulgaria | 1.2 (0.52, 1.88) | 0.001* | 1.33 (0.61, 2.04) | <0.001* | 0.37 (-0.42, 1.16) | 0.365 | 0.34 (-0.25, 0.92) | 0.262 | 1.19 (0.49, 1.91) | 0.001* | 0.24 (-0.33, 0.82) | 0.412 |
| South Sudan | -0.26 (-0.45, -0.08) | 0.005* | 0.01 (-0.29, 0.31) | 0.961 | -0.37 (-0.41, -0.33) | <0.001* | -0.49 (-0.53, -0.45) | <0.001* | -0.19 (-0.41, 0.02) | 0.078 | -0.49 (-0.53, -0.46) | <0.001* |
| Sudan | 1.71 (1.52, 1.89) | <0.001* | 2.83 (2.53, 3.14) | <0.001* | 0.37 (0.33, 0.42) | <0.001* | 0.33 (0.26, 0.4) | <0.001* | 2.21 (1.92, 2.5) | <0.001* | 0.27 (0.22, 0.32) | <0.001* |
| Azerbaijan | 0.73 (0.43, 1.02) | <0.001* | 1.18 (0.86, 1.5) | <0.001* | -0.36 (-0.58, -0.13) | 0.002* | -0.67 (-0.9, -0.45) | <0.001* | 0.93 (0.67, 1.2) | <0.001* | -0.75 (-0.98, -0.53) | <0.001* |
| Djibouti | 1 (0.76, 1.25) | <0.001* | 1.91 (1.56, 2.25) | <0.001* | 0.36 (0.16, 0.57) | <0.001* | 0.26 (0.21, 0.3) | <0.001* | 1.28 (1.02, 1.55) | <0.001* | 0.23 (0.19, 0.27) | <0.001* |
| Andorra | 1.02 (0.78, 1.27) | <0.001* | 1.21 (0.95, 1.46) | <0.001* | -0.34 (-0.54, -0.15) | 0.001* | -0.12 (-0.33, 0.09) | 0.246 | 1.12 (0.86, 1.37) | <0.001* | -0.32 (-0.52, -0.12) | 0.002* |
| Democratic People's Republic of Korea | -0.26 (-0.42, -0.1) | 0.001* | -0.25 (-0.44, -0.06) | 0.011* | -0.32 (-0.36, -0.28) | <0.001* | -0.23 (-0.31, -0.15) | <0.001* | -0.23 (-0.39, -0.06) | 0.006* | -0.23 (-0.28, -0.18) | <0.001* |
| Russian Federation | 1.05 (-0.09, 2.19) | 0.071 | 1.21 (0.13, 2.31) | 0.029* | -0.31 (-1.37, 0.76) | 0.566 | -0.28 (-1.45, 0.9) | 0.64 | 1.16 (0.01, 2.32) | 0.048* | -0.5 (-1.65, 0.67) | 0.4 |
| Congo | 0.27 (0.01, 0.53) | 0.040* | 1.04 (0.64, 1.43) | <0.001* | -0.31 (-0.6, -0.02) | 0.039* | -0.44 (-0.69, -0.2) | <0.001* | 0.55 (0.26, 0.85) | <0.001* | -0.47 (-0.71, -0.22) | <0.001* |
| Netherlands | 1.59 (0.87, 2.32) | <0.001* | 1.81 (1.08, 2.55) | <0.001* | 0.3 (0, 0.6) | 0.049* | 0.41 (0, 0.82) | 0.05 | 1.71 (0.95, 2.47) | <0.001* | 0.2 (-0.19, 0.59) | 0.321 |
| Afghanistan | 0.87 (0.78, 0.95) | <0.001* | 1.79 (1.75, 1.84) | <0.001* | 0.29 (0.24, 0.33) | <0.001* | 0.25 (0.18, 0.31) | <0.001* | 1.16 (1.09, 1.24) | <0.001* | 0.23 (0.16, 0.29) | <0.001* |
| Mexico | 1.95 (1.47, 2.43) | <0.001* | 2.91 (1.99, 3.85) | <0.001* | -0.28 (-0.61, 0.06) | 0.107 | 0.16 (-0.27, 0.6) | 0.462 | 2.4 (1.46, 3.34) | <0.001* | 0.04 (-0.39, 0.47) | 0.853 |
| Central African Republic | -0.31 (-0.58, -0.04) | 0.022* | -0.31 (-0.57, -0.05) | 0.021* | -0.28 (-0.47, -0.1) | 0.002* | -0.38 (-0.57, -0.18) | <0.001* | -0.32 (-0.56, -0.08) | 0.010* | -0.38 (-0.57, -0.19) | <0.001* |
| Cuba | 1.2 (0.37, 2.04) | 0.005* | 1.44 (0.6, 2.28) | 0.001* | -0.27 (-0.97, 0.43) | 0.444 | -0.23 (-1.02, 0.56) | 0.562 | 1.28 (0.43, 2.13) | 0.003* | -0.48 (-1.26, 0.3) | 0.226 |
| Philippines | 0.19 (-0.1, 0.49) | 0.201 | 0.47 (0, 0.94) | 0.05 | -0.27 (-0.43, -0.11) | 0.001* | -0.14 (-0.29, 0.02) | 0.089 | 0.32 (-0.02, 0.66) | 0.066 | -0.16 (-0.32, 0) | 0.055 |
| Democratic Republic of the Congo | 0.07 (-0.06, 0.2) | 0.285 | 0.61 (0.28, 0.95) | <0.001* | -0.27 (-0.34, -0.21) | <0.001* | -0.36 (-0.43, -0.29) | <0.001* | 0.29 (-0.01, 0.6) | 0.054 | -0.39 (-0.46, -0.31) | <0.001* |
| Chile | 2.32 (2.04, 2.6) | <0.001* | 3.31 (2.96, 3.65) | <0.001* | 0.27 (0.08, 0.46) | 0.005* | 0.41 (-0.02, 0.83) | 0.06 | 2.65 (2.37, 2.93) | <0.001* | 0.25 (-0.16, 0.66) | 0.232 |
| Ukraine | 0.92 (-0.09, 1.93) | 0.074 | 1.06 (0.04, 2.08) | 0.041* | -0.26 (-1.23, 0.71) | 0.597 | -0.09 (-1.13, 0.97) | 0.87 | 1.12 (0.11, 2.14) | 0.029* | -0.17 (-1.22, 0.9) | 0.76 |
| India | 1.11 (0.53, 1.69) | <0.001* | 2.43 (1.85, 3.01) | <0.001* | -0.26 (-0.96, 0.44) | 0.461 | -0.14 (-0.69, 0.41) | 0.618 | 1.66 (1.08, 2.25) | <0.001* | -0.2 (-0.75, 0.36) | 0.488 |
| Burkina Faso | 0.78 (0.52, 1.05) | <0.001* | 1.58 (1.25, 1.92) | <0.001* | 0.26 (0.03, 0.49) | 0.030* | 0.25 (-0.03, 0.53) | 0.083 | 1.04 (0.71, 1.37) | <0.001* | 0.23 (-0.02, 0.48) | 0.075 |
| Namibia | 1.27 (0.8, 1.73) | <0.001* | 2.32 (2.08, 2.56) | <0.001* | 0.26 (0, 0.52) | 0.046* | 0.06 (-0.24, 0.35) | 0.702 | 1.63 (1.29, 1.97) | <0.001* | 0.01 (-0.27, 0.3) | 0.946 |
| Kyrgyzstan | 0.99 (0.46, 1.51) | <0.001* | 1.48 (0.98, 1.97) | <0.001* | -0.25 (-0.89, 0.4) | 0.451 | -0.33 (-0.9, 0.24) | 0.257 | 1.23 (0.74, 1.71) | <0.001* | -0.41 (-0.98, 0.17) | 0.163 |
| Belgium | 0.99 (0.71, 1.28) | <0.001* | 1.21 (0.93, 1.49) | <0.001* | -0.25 (-0.55, 0.04) | 0.092 | -0.33 (-0.67, 0.02) | 0.062 | 1.08 (0.79, 1.36) | <0.001* | -0.53 (-0.86, -0.2) | 0.002* |
| Tonga | 0.6 (0.41, 0.8) | <0.001* | 0.81 (0.54, 1.08) | <0.001* | 0.25 (0.15, 0.36) | <0.001* | 0.23 (0.16, 0.29) | <0.001* | 0.63 (0.42, 0.84) | <0.001* | 0.21 (0.15, 0.28) | <0.001* |
| Indonesia | 1.06 (0.9, 1.22) | <0.001* | 1.63 (1.48, 1.78) | <0.001* | 0.24 (0.16, 0.32) | <0.001* | 0.13 (0.09, 0.18) | <0.001* | 1.29 (1.17, 1.41) | <0.001* | 0.09 (0.05, 0.14) | <0.001* |
| Gabon | 0.42 (0.06, 0.79) | 0.023* | 1.22 (0.82, 1.62) | <0.001* | -0.23 (-0.52, 0.06) | 0.117 | -0.43 (-0.75, -0.11) | 0.009* | 0.73 (0.37, 1.09) | <0.001* | -0.46 (-0.78, -0.14) | 0.005* |
| Canada | 1.07 (0.8, 1.33) | <0.001* | 1.26 (0.99, 1.53) | <0.001* | -0.23 (-0.38, -0.08) | 0.003* | -0.06 (-0.23, 0.12) | 0.522 | 1.11 (0.84, 1.38) | <0.001* | -0.24 (-0.41, -0.07) | 0.007* |
| Montenegro | 1.49 (1.32, 1.66) | <0.001* | 1.69 (1.54, 1.85) | <0.001* | 0.23 (0, 0.46) | 0.049* | 0.25 (0.07, 0.43) | 0.006* | 1.58 (1.41, 1.75) | <0.001* | 0.14 (-0.04, 0.31) | 0.122 |
| Tunisia | 1.82 (1.69, 1.96) | <0.001* | 2.64 (2.49, 2.79) | <0.001* | -0.22 (-0.38, -0.06) | 0.006* | -0.13 (-0.29, 0.03) | 0.111 | 2.26 (2.1, 2.42) | <0.001* | -0.28 (-0.44, -0.13) | <0.001* |
| Benin | 0.63 (0.44, 0.82) | <0.001* | 1.29 (1.01, 1.57) | <0.001* | 0.22 (0.14, 0.29) | <0.001* | 0.13 (0.04, 0.22) | 0.004* | 0.86 (0.66, 1.06) | <0.001* | 0.11 (0.02, 0.2) | 0.018* |
| Palau | 0.36 (0.25, 0.48) | <0.001* | 0.5 (0.37, 0.63) | <0.001* | -0.21 (-0.26, -0.15) | <0.001* | -0.22 (-0.27, -0.16) | <0.001* | 0.46 (0.3, 0.61) | <0.001* | -0.26 (-0.31, -0.21) | <0.001* |
| Cambodia | 1.03 (0.95, 1.12) | <0.001* | 2.07 (1.97, 2.18) | <0.001* | -0.21 (-0.25, -0.17) | <0.001* | -0.3 (-0.34, -0.25) | <0.001* | 1.47 (1.35, 1.58) | <0.001* | -0.35 (-0.4, -0.31) | <0.001* |
| Haiti | 0.21 (0.05, 0.36) | 0.008* | 0.84 (0.64, 1.03) | <0.001* | -0.2 (-0.27, -0.12) | <0.001* | -0.29 (-0.42, -0.16) | <0.001* | 0.42 (0.26, 0.57) | <0.001* | -0.3 (-0.43, -0.17) | <0.001* |
| Greece | 1.4 (1.11, 1.69) | <0.001* | 1.61 (1.05, 2.18) | <0.001* | 0.2 (-0.14, 0.55) | 0.248 | 0.37 (-0.04, 0.79) | 0.08 | 1.51 (0.95, 2.07) | <0.001* | 0.22 (-0.15, 0.59) | 0.25 |
| Senegal | 0.56 (-0.07, 1.21) | 0.082 | 1.3 (0.67, 1.93) | <0.001* | 0.19 (-0.31, 0.69) | 0.455 | 0.04 (-0.59, 0.68) | 0.901 | 0.89 (0.28, 1.51) | 0.004* | 0.02 (-0.61, 0.66) | 0.943 |
| Puerto Rico | 1.74 (1.6, 1.87) | <0.001* | 2.14 (1.99, 2.29) | <0.001* | 0.18 (0.07, 0.29) | 0.003* | 0.43 (0.32, 0.54) | <0.001* | 1.88 (1.74, 2.02) | <0.001* | 0.3 (0.19, 0.42) | <0.001* |
| Viet Nam | 2.09 (1.91, 2.27) | <0.001* | 3.13 (2.92, 3.34) | <0.001* | 0.17 (0.11, 0.22) | <0.001* | 0.28 (0.21, 0.36) | <0.001* | 2.6 (2.41, 2.79) | <0.001* | 0.17 (0.1, 0.24) | <0.001* |
| Mali | 0.42 (0.34, 0.5) | <0.001* | 1.29 (1.09, 1.5) | <0.001* | -0.16 (-0.27, -0.05) | 0.003* | -0.25 (-0.39, -0.12) | <0.001* | 0.75 (0.53, 0.97) | <0.001* | -0.28 (-0.42, -0.13) | <0.001* |
| Monaco | 1.08 (0.91, 1.26) | <0.001* | 1.14 (0.96, 1.31) | <0.001* | 0.16 (0.08, 0.25) | <0.001* | 0.2 (0.13, 0.28) | <0.001* | 1.08 (0.93, 1.22) | <0.001* | 0.05 (-0.03, 0.13) | 0.209 |
| Japan | 2.69 (2.27, 3.11) | <0.001* | 3.16 (2.74, 3.58) | <0.001* | -0.15 (-0.34, 0.05) | 0.145 | 0.54 (0.37, 0.71) | <0.001* | 2.89 (2.47, 3.31) | <0.001* | 0.23 (0.03, 0.43) | 0.025* |
| Finland | 1.09 (0.91, 1.26) | <0.001* | 1.33 (1.14, 1.52) | <0.001* | -0.15 (-0.3, 0) | 0.055 | -0.21 (-0.31, -0.1) | <0.001* | 1.18 (1, 1.37) | <0.001* | -0.39 (-0.48, -0.29) | <0.001* |
| United States Virgin Islands | 0.39 (0.29, 0.49) | <0.001* | 0.63 (0.51, 0.75) | <0.001* | -0.15 (-0.27, -0.03) | 0.017* | -0.29 (-0.41, -0.17) | <0.001* | 0.49 (0.36, 0.61) | <0.001* | -0.34 (-0.42, -0.26) | <0.001* |
| Liberia | 0.44 (0.23, 0.65) | <0.001* | 1.36 (1.14, 1.58) | <0.001* | -0.14 (-0.32, 0.04) | 0.12 | -0.23 (-0.41, -0.06) | 0.007* | 0.81 (0.61, 1.01) | <0.001* | -0.26 (-0.42, -0.09) | 0.003* |
| Guinea-Bissau | 0.5 (0.44, 0.56) | <0.001* | 1.13 (1.05, 1.21) | <0.001* | 0.13 (0.06, 0.19) | <0.001* | -0.01 (-0.09, 0.07) | 0.842 | 0.67 (0.54, 0.81) | <0.001* | -0.02 (-0.1, 0.06) | 0.592 |
| United Republic of Tanzania | 0.67 (0.38, 0.97) | <0.001* | 1.45 (1.08, 1.83) | <0.001* | 0.13 (0.02, 0.25) | 0.021* | 0.08 (-0.01, 0.18) | 0.095 | 0.87 (0.39, 1.34) | <0.001* | 0.07 (-0.03, 0.17) | 0.174 |
| Cameroon | 0.47 (0.33, 0.6) | <0.001* | 1.24 (1.02, 1.46) | <0.001* | -0.12 (-0.22, -0.02) | 0.020* | -0.21 (-0.31, -0.12) | <0.001* | 0.75 (0.58, 0.93) | <0.001* | -0.24 (-0.33, -0.15) | <0.001* |
| Ireland | 1.73 (1.26, 2.2) | <0.001* | 2.1 (1.56, 2.64) | <0.001* | -0.11 (-0.37, 0.15) | 0.421 | -0.01 (-0.32, 0.3) | 0.929 | 1.84 (1.35, 2.34) | <0.001* | -0.3 (-0.58, -0.01) | 0.041* |
| Seychelles | 1.08 (0.77, 1.38) | <0.001* | 1.43 (1.15, 1.72) | <0.001* | 0.11 (-0.18, 0.39) | 0.463 | -0.04 (-0.35, 0.28) | 0.803 | 1.2 (0.94, 1.45) | <0.001* | -0.11 (-0.42, 0.21) | 0.505 |
| Somalia | -0.06 (-0.14, 0.03) | 0.212 | 0.2 (-0.08, 0.48) | 0.167 | -0.11 (-0.17, -0.04) | 0.002* | -0.25 (-0.33, -0.18) | <0.001* | -0.04 (-0.31, 0.23) | 0.781 | -0.26 (-0.33, -0.18) | <0.001* |
| Lithuania | 0.87 (0.43, 1.3) | <0.001* | 1.18 (0.09, 2.28) | 0.034* | -0.1 (-0.44, 0.25) | 0.588 | -0.24 (-0.63, 0.16) | 0.249 | 1.04 (0.57, 1.52) | <0.001* | -0.31 (-0.71, 0.09) | 0.124 |
| Togo | 0.25 (0.03, 0.47) | 0.026* | 0.78 (0.47, 1.09) | <0.001* | -0.1 (-0.31, 0.12) | 0.381 | -0.19 (-0.4, 0.01) | 0.066 | 0.47 (0.26, 0.68) | <0.001* | -0.22 (-0.42, -0.01) | 0.037* |
| Angola | 0.56 (0.33, 0.79) | <0.001* | 1.62 (1.32, 1.91) | <0.001* | -0.1 (-0.25, 0.05) | 0.174 | -0.32 (-0.5, -0.14) | 0.001* | 0.95 (0.7, 1.2) | <0.001* | -0.35 (-0.53, -0.17) | <0.001* |
| Timor-Leste | 0.93 (0.51, 1.34) | <0.001* | 1.86 (1.24, 2.48) | <0.001* | -0.09 (-0.35, 0.17) | 0.504 | -0.12 (-0.3, 0.07) | 0.223 | 1.31 (0.78, 1.84) | <0.001* | -0.16 (-0.34, 0.02) | 0.089 |
| Ghana | 0.61 (0.38, 0.84) | <0.001* | 1.45 (1.16, 1.73) | <0.001* | -0.09 (-0.22, 0.04) | 0.164 | -0.15 (-0.31, 0.01) | 0.065 | 0.94 (0.63, 1.25) | <0.001* | -0.21 (-0.35, -0.06) | 0.006* |
| Kiribati | -0.01 (-0.1, 0.09) | 0.854 | 0.11 (-0.04, 0.25) | 0.147 | -0.09 (-0.18, 0) | 0.05 | -0.26 (-0.35, -0.18) | <0.001* | -0.01 (-0.2, 0.19) | 0.931 | -0.27 (-0.36, -0.18) | <0.001* |
| Guinea | 0.48 (0.38, 0.59) | <0.001* | 1.15 (1.01, 1.3) | <0.001* | 0.09 (-0.01, 0.18) | 0.072 | 0.07 (-0.02, 0.17) | 0.137 | 0.71 (0.57, 0.85) | <0.001* | 0.06 (-0.04, 0.16) | 0.231 |
| Bhutan | 1.38 (1.23, 1.53) | <0.001* | 3.06 (2.81, 3.31) | <0.001* | -0.07 (-0.18, 0.03) | 0.182 | -0.34 (-0.48, -0.21) | <0.001* | 2.06 (1.82, 2.29) | <0.001* | -0.42 (-0.55, -0.28) | <0.001* |
| Malaysia | 1.56 (1.38, 1.73) | <0.001* | 2.29 (2.09, 2.49) | <0.001* | -0.06 (-0.25, 0.12) | 0.503 | -0.01 (-0.16, 0.14) | 0.854 | 1.87 (1.7, 2.04) | <0.001* | -0.13 (-0.28, 0.02) | 0.101 |
| Oman | 2.21 (1.56, 2.86) | <0.001* | 3.05 (2.41, 3.7) | <0.001* | 0.06 (-0.2, 0.32) | 0.637 | 0 (-0.31, 0.32) | 0.992 | 2.66 (2.19, 3.13) | <0.001* | -0.18 (-0.49, 0.14) | 0.271 |
| Croatia | 0.44 (0.24, 0.63) | <0.001* | 0.88 (0.65, 1.11) | <0.001* | 0.06 (-0.18, 0.3) | 0.632 | -0.02 (-0.25, 0.21) | 0.846 | 0.61 (0.4, 0.81) | <0.001* | -0.04 (-0.27, 0.19) | 0.745 |
| Spain | 1.33 (1.11, 1.56) | <0.001* | 1.49 (1.04, 1.95) | <0.001* | -0.06 (-0.18, 0.06) | 0.307 | -0.1 (-0.2, 0) | 0.057 | 1.37 (0.93, 1.81) | <0.001* | -0.33 (-0.42, -0.24) | <0.001* |
| Tuvalu | 0.65 (0.56, 0.75) | <0.001* | 1.13 (0.92, 1.34) | <0.001* | -0.06 (-0.11, 0) | 0.054 | -0.1 (-0.16, -0.04) | 0.001* | 0.87 (0.66, 1.08) | <0.001* | -0.13 (-0.19, -0.07) | <0.001* |
| Nauru | 0.56 (0.41, 0.7) | <0.001* | 0.73 (0.55, 0.9) | <0.001* | 0.06 (-0.03, 0.14) | 0.176 | 0.14 (0.04, 0.24) | 0.006* | 0.68 (0.51, 0.85) | <0.001* | 0.11 (0.01, 0.22) | 0.033* |
| Niue | 1.1 (0.94, 1.27) | <0.001* | 1.34 (1.16, 1.53) | <0.001* | 0.05 (-0.14, 0.23) | 0.616 | 0.15 (-0.02, 0.31) | 0.077 | 1.29 (1.12, 1.46) | <0.001* | 0.07 (-0.11, 0.26) | 0.448 |
| Bolivia (Pluractional State of) | 1.23 (0.97, 1.49) | <0.001* | 2.56 (1.93, 3.19) | <0.001* | -0.05 (-0.13, 0.02) | 0.167 | -0.28 (-0.37, -0.19) | <0.001* | 1.75 (1.34, 2.17) | <0.001* | -0.32 (-0.42, -0.22) | <0.001* |
| Samoa | 0.54 (0.36, 0.71) | <0.001* | 0.74 (0.4, 1.09) | <0.001* | 0.05 (-0.04, 0.14) | 0.249 | 0.1 (0, 0.2) | 0.040* | 0.6 (0.26, 0.93) | <0.001* | 0.06 (-0.02, 0.15) | 0.149 |
| New Zealand | 1.18 (0.82, 1.54) | <0.001* | 1.48 (1.12, 1.85) | <0.001* | 0.04 (-0.38, 0.47) | 0.841 | 0.07 (-0.36, 0.49) | 0.766 | 1.31 (0.95, 1.66) | <0.001* | -0.06 (-0.49, 0.36) | 0.775 |
| Madagascar | 0.31 (0.02, 0.61) | 0.039* | 0.83 (0.45, 1.22) | <0.001* | 0.04 (-0.29, 0.38) | 0.809 | -0.1 (-0.38, 0.18) | 0.473 | 0.57 (0.14, 1) | 0.010* | -0.11 (-0.39, 0.16) | 0.422 |
| Comoros | 0.77 (0.48, 1.06) | <0.001* | 1.78 (1.67, 1.9) | <0.001* | 0.03 (-0.44, 0.51) | 0.891 | -0.05 (-0.49, 0.39) | 0.825 | 1.1 (0.78, 1.41) | <0.001* | -0.07 (-0.51, 0.37) | 0.742 |
| Saint Lucia | 0.86 (0.61, 1.11) | <0.001* | 1.28 (1, 1.55) | <0.001* | 0.03 (-0.31, 0.37) | 0.852 | 0.03 (-0.26, 0.32) | 0.849 | 1.03 (0.65, 1.41) | <0.001* | -0.02 (-0.32, 0.28) | 0.897 |
| Libya | 1.31 (0.82, 1.79) | <0.001* | 1.83 (1.3, 2.36) | <0.001* | 0.02 (-0.46, 0.5) | 0.943 | 0.06 (-0.42, 0.53) | 0.818 | 1.55 (1.07, 2.03) | <0.001* | -0.02 (-0.54, 0.49) | 0.93 |
| Nepal | 1.03 (0.92, 1.13) | <0.001* | 2.34 (2.17, 2.51) | <0.001* | -0.02 (-0.09, 0.05) | 0.595 | -0.28 (-0.42, -0.14) | <0.001* | 1.57 (1.42, 1.73) | <0.001* | -0.33 (-0.47, -0.2) | <0.001* |
| San Marino | 1.04 (0.95, 1.13) | <0.001* | 1.22 (1.12, 1.32) | <0.001* | -0.01 (-0.16, 0.14) | 0.881 | 0.22 (0.11, 0.32) | <0.001* | 1.09 (0.99, 1.19) | <0.001* | 0.08 (-0.03, 0.18) | 0.142 |
| Niger | 0.47 (0.23, 0.72) | <0.001* | 1.23 (1.05, 1.41) | <0.001* | 0.01 (-0.12, 0.14) | 0.866 | -0.1 (-0.25, 0.05) | 0.212 | 0.72 (0.46, 0.97) | <0.001* | -0.11 (-0.26, 0.04) | 0.14 |
| Tokelau | 1.16 (1.09, 1.22) | <0.001* | 1.73 (1.64, 1.82) | <0.001* | -0.01 (-0.03, 0.02) | 0.578 | 0.04 (0.01, 0.07) | 0.005* | 1.42 (1.34, 1.51) | <0.001* | -0.02 (-0.05, 0.01) | 0.209 |
| Poland | 1.9 (1.48, 2.31) | <0.001* | 2.43 (1.94, 2.92) | <0.001* | 0 (-0.3, 0.31) | 0.981 | -0.2 (-0.56, 0.15) | 0.26 | 2.2 (1.8, 2.59) | <0.001* | -0.4 (-0.75, -0.04) | 0.028* |

AAPC: average annual percentage change; DALYs: disability-adjusted life-years; YLDs: years lived with disability; YLLs: years of life lost; CI: confidence interval; *: P<0.05.

Table S5 ASR (1990, 2019) and AAPCs (1990-2019) in GBD regions

| **GBD region** | **ASR per 100,000 population (95%UI)** | | **AAPC** | |
| --- | --- | --- | --- | --- |
|  | **1990** | **2019** | **AAPC (%) (95% CI)** | **P Value** |
| **Incidence** |  |  |  |  |
| Andean Latin America | 7.57 (6.06, 8.74) | 9.75 (7.65, 12.67) | 0.77 (0.38, 1.16) | <0.001* |
| Australasia | 9.46 (8.88, 10.03) | 11.26 (9.13, 13.94) | 0.67 (0.34, 1.01) | <0.001* |
| Caribbean | 11.61 (10.82, 12.43) | 17.83 (15.11, 20.97) | 1.59 (1.40, 1.78) | <0.001* |
| Central Asia | 10.77 (10.18, 11.44) | 11.72 (10.47, 13.11) | 0.16 (0.00, 0.32) | 0.044* |
| Central Europe | 13.83 (13.32, 14.55) | 20.52 (17.68, 23.86) | 1.34 (1.05, 1.63) | <0.001* |
| Central Latin America | 4.10 (3.94, 4.26) | 6.4 (5.39, 7.58) | 1.60 (1.36, 1.83) | <0.001* |
| Central Sub-Saharan Africa | 2.82 (2.11, 3.96) | 3.01 (2.14, 4.32) | 0.22 (0.09, 0.35) | 0.001* |
| East Asia | 5.17 (3.92, 6.33) | 6.55 (5.07, 8.8) | 0.83 (0.63, 1.03) | <0.001* |
| Eastern Europe | 20.42 (19.56, 21.44) | 27.50 (23.25, 32.58) | 1.09 (-0.02, 2.21) | 0.054 |
| Eastern Sub-Saharan Africa | 3.27 (2.32, 4.01) | 3.70 (2.64, 4.57) | 0.40 (0.30, 0.50) | <0.001* |
| High-income Asia Pacific | 6.74 (5.95, 7.08) | 11.32 (9.21, 13.68) | 1.77 (1.44, 2.11) | <0.001* |
| High-income North America | 19.15 (18.49, 19.68) | 27.82 (23.11, 33.44) | 1.29 (0.91, 1.67) | <0.001* |
| North Africa and Middle East | 3.10 (2.36, 3.73) | 5.41 (3.71, 6.39) | 1.90 (1.66, 2.15) | <0.001* |
| Oceania | 6.75 (4.79, 8.38) | 8.58 (4.86, 11.31) | 0.82 (0.67, 0.96) | <0.001* |
| South Asia | 2.17 (1.71, 2.74) | 2.94 (2.34, 3.61) | 1.11 (0.56, 1.65) | <0.001* |
| Southeast Asia | 4.70 (3.36, 5.46) | 6.23 (4.10, 7.47) | 0.98 (0.88, 1.09) | <0.001* |
| Southern Latin America | 6.97 (6.59, 7.36) | 8.23 (6.43, 10.38) | 0.58 (0.41, 0.76) | <0.001* |
|  | 3.48 (2.82, 4.05) | 5.08 (3.57, 5.87) | 1.28 (0.67, 1.89) | <0.001* |
| Tropical Latin America | 5.92 (5.62, 6.2) | 6.97 (6.50, 7.48) | 0.64 (0.53, 0.74) | <0.001* |
| Western Europe | 13.14 (12.68, 13.52) | 19.62 (16.98, 22.47) | 1.39 (1.11, 1.67) | <0.001* |
| Western Sub-Saharan Africa | 2.13 (1.73, 3.16) | 2.64 (2.13, 3.58) | 0.74 (0.55, 0.92) | <0.001* |
| **Prevalence** |  |  |  |  |
| Andean Latin America | 35.12 (27.44, 40.73) | 63.08 (49.09, 81.99) | 1.93 (1.57, 2.3) | <0.001* |
| Australasia | 66.19 (61.59, 70.59) | 84.69 (68.67, 104.84) | 0.87 (0.57, 1.18) | <0.001* |
| Caribbean | 71.49 (66.71, 76.18) | 119.94 (101.15, 141.96) | 1.88 (1.69, 2.07) | <0.001* |
| Central Asia | 68.16 (64.21, 72.57) | 82.73 (73.54, 92.85) | 0.56 (0.40, 0.73) | <0.001* |
| Central Europe | 93.43 (90.01, 98.27) | 156.65 (134.98, 182.84) | 1.77 (1.50, 2.03) | <0.001* |
| Central Latin America | 21.89 (21.09, 22.73) | 44.77 (37.48, 53.37) | 2.59 (2.32, 2.85) | <0.001* |
| Central Sub-Saharan Africa | 9.24 (6.8, 12.93) | 12.06 (8.43, 17.39) | 0.93 (0.57, 1.30) | <0.001* |
| East Asia | 29.71 (21.81, 36.65) | 50.67 (39.13, 67.54) | 1.87 (1.65, 2.10) | <0.001* |
| Eastern Europe | 150.08 (143.5, 157.97) | 215.08 (180.88, 255.81) | 1.29 (0.07, 2.53) | 0.039* |
| Eastern Sub-Saharan Africa | 10.33 (7.21, 12.67) | 15.41 (11.22, 19.22) | 1.39 (1.04, 1.73) | <0.001* |
| High-income Asia Pacific | 47.14 (41.55, 49.75) | 91.77 (74.53, 111.17) | 2.29 (1.94, 2.63) | <0.001* |
| High-income North America | 147.65 (143.05, 151.8) | 222.45 (184.63, 267.82) | 1.42 (1.01, 1.82) | <0.001* |
| North Africa and Middle East | 16.04 (11.80, 19.37) | 37.47 (25.06, 44.38) | 2.94 (2.68, 3.19) | <0.001* |
| Oceania | 33.58 (24.13, 41.64) | 45.14 (25.26, 60.10) | 0.99 (0.94, 1.05) | <0.001* |
| South Asia | 7.71 (6.08, 9.66) | 15.57 (12.25, 19.09) | 2.50 (2.10, 2.91) | <0.001* |
| Southeast Asia | 25.28 (17.55, 29.39) | 40.36 (25.81, 48.71) | 1.63 (1.47, 1.80) | <0.001* |
| Southern Latin America | 41.24 (38.74, 43.99) | 57.14 (44.19, 72.67) | 1.15 (0.96, 1.33) | <0.001* |
| Southern Sub-Saharan Africa | 15.75 (12.82, 18.46) | 24.97 (18.05, 29.17) | 1.56 (1.10, 2.01) | <0.001* |
| Tropical Latin America | 30.72 (29.24, 32.23) | 45.80 (42.79, 49.16) | 1.43 (1.28, 1.58) | <0.001* |
| Western Europe | 98.31 (95.17, 101.18) | 156.49 (135.18, 179.28) | 1.60 (1.30, 1.91) | <0.001* |
| Western Sub-Saharan Africa | 7.46 (5.98, 10.64) | 11.25 (8.92, 14.91) | 1.40 (1.06, 1.74) | <0.001* |
| **Deaths** |  |  |  |  |
| Andean Latin America | 4.53 (3.67, 5.21) | 3.53 (2.80, 4.63) | -0.85 (-0.95, -0.74) | <0.001* |
| Australasia | 2.62 (2.46, 2.77) | 2.40 (2.13, 2.65) | -0.27 (-0.50, -0.04) | 0.022* |
| Caribbean | 4.54 (4.18, 4.99) | 5.68 (4.86, 6.62) | 0.83 (0.30, 1.37) | 0.002* |
| Central Asia | 4.02 (3.80, 4.26) | 3.20 (2.88, 3.57) | -0.79 (-1.08, -0.49) | <0.001* |
| Central Europe | 4.23 (4.05, 4.47) | 3.79 (3.28, 4.36) | -0.38 (-0.59, -0.17) | <0.001* |
| Central Latin America | 2.08 (1.97, 2.17) | 1.84 (1.57, 2.16) | -0.39 (-0.63, -0.15) | 0.001* |
| Central Sub-Saharan Africa | 2.10 (1.59, 2.93) | 1.97 (1.42, 2.80) | -0.26 (-0.31, -0.21) | <0.001* |
| East Asia | 2.35 (1.80, 2.86) | 1.19 (0.93, 1.65) | -2.31 (-2.44, -2.18) | <0.001* |
| Eastern Europe | 4.45 (4.27, 4.64) | 3.92 (3.39, 4.51) | -0.37 (-1.12, 0.37) | 0.326 |
| Eastern Sub-Saharan Africa | 2.48 (1.78, 3.09) | 2.41 (1.70, 3.01) | -0.09 (-0.18, 0.00) | 0.056 |
| High-income Asia Pacific | 1.94 (1.71, 2.04) | 1.47 (1.30, 1.58) | -0.96 (-1.13, -0.79) | <0.001* |
| High-income North America | 2.92 (2.76, 3.01) | 3.23 (3.03, 3.38) | 0.34 (0.23, 0.46) | <0.001* |
| North Africa and Middle East | 1.62 (1.25, 1.99) | 1.58 (1.17, 1.85) | -0.08 (-0.25, 0.09) | 0.368 |
| Oceania | 3.52 (2.45, 4.39) | 4.18 (2.44, 5.5) | 0.58 (0.52, 0.64) | <0.001* |
| South Asia | 1.57 (1.23, 2.02) | 1.47 (1.16, 1.87) | -0.20 (-0.71, 0.31) | 0.449 |
| Southeast Asia | 2.31 (1.73, 2.73) | 2.21 (1.60, 2.60) | -0.15 (-0.26, -0.05) | 0.003* |
| Southern Latin America | 2.99 (2.82, 3.15) | 2.4 (2.19, 2.62) | -0.76 (-0.89, -0.63) | <0.001* |
| Southern Sub-Saharan Africa | 2.09 (1.65, 2.48) | 2.77 (1.92, 3.18) | 0.96 (0.43, 1.49) | <0.001* |
| Tropical Latin America | 3.08 (2.89, 3.22) | 2.40 (2.20, 2.57) | -0.81 (-0.90, -0.71) | <0.001* |
| Western Europe | 2.61 (2.46, 2.69) | 2.59 (2.35, 2.74) | 0.00 (-0.15, 0.16) | 0.968 |
| Western Sub-Saharan Africa | 1.54 (1.24, 2.32) | 1.66 (1.36, 2.27) | 0.28 (0.20, 0.35) | <0.001* |
| **DALYs** |  |  |  |  |
| Andean Latin America | 113.36 (90.87, 130.54) | 85.55 (66.72, 112.49) | -0.96 (-1.07, -0.85) | <0.001* |
| Australasia | 61.71 (58.26, 65.17) | 56.30 (50.79, 61.96) | -0.29 (-0.61, 0.03) | 0.079 |
| Caribbean | 119.52 (109.4, 131.55) | 146.98 (124.22, 173.54) | 0.76 (0.28, 1.25) | 0.002* |
| Central Asia | 112.56 (106.46, 119.13) | 89.73 (80.18, 100.92) | -0.81 (-1.08, -0.53) | <0.001* |
| Central Europe | 108.58 (104.28, 114.78) | 96.48 (83.29, 111.56) | -0.41 (-0.64, -0.19) | <0.001* |
| Central Latin America | 50.41 (48.50, 52.56) | 47.36 (40.25, 55.58) | -0.20 (-0.41, 0.02) | 0.073 |
| Central Sub-Saharan Africa | 53.40 (40.06, 73.87) | 48.58 (34.5, 69.88) | -0.37 (-0.43, -0.31) | <0.001* |
| East Asia | 68.79 (50.13, 84.60) | 35.70 (28.39, 47.85) | -2.22 (-2.36, -2.08) | <0.001* |
| Eastern Europe | 124.15 (118.61, 130.58) | 110.79 (95.86, 128.31) | -0.32 (-1.16, 0.54) | 0.467 |
| Eastern Sub-Saharan Africa | 61.55 (43.74, 76.34) | 56.64 (40.16, 70.28) | -0.28 (-0.37, -0.20) | <0.001* |
| High-income Asia Pacific | 49.96 (42.00, 52.95) | 42.38 (38.85, 45.96) | -0.57 (-0.74, -0.39) | <0.001* |
| High-income North America | 72.79 (69.27, 76.36) | 85.62 (79.99, 91.48) | 0.56 (0.44, 0.68) | <0.001* |
| North Africa and Middle East | 40.91 (31.33, 49.31) | 39.33 (28.15, 46.59) | -0.14 (-0.30, 0.03) | 0.103 |
| Oceania | 94.6 (65.12, 118.8) | 113.23 (63.01, 151.27) | 0.61 (0.55, 0.67) | <0.001* |
| South Asia | 36.89 (29.05, 46.71) | 36.23 (28.51, 45.91) | -0.03 (-0.55, 0.50) | 0.925 |
| Southeast Asia | 63.15 (45.15, 75.00) | 59.84 (39.99, 71.06) | -0.18 (-0.23, -0.14) | <0.001* |
| Southern Latin America | 72.81 (68.69, 76.97) | 56.68 (51.56, 62.19) | -0.88 (-1.00, -0.75) | <0.001* |
| Southern Sub-Saharan Africa | 49.39 (39.85, 58.05) | 62.63 (44.04, 73.04) | 0.78 (0.20, 1.36) | 0.008* |
| Tropical Latin America | 71.87 (68.47, 75.08) | 57.25 (53.41, 61.27) | -0.72 (-0.82, -0.62) | <0.001* |
| Western Europe | 62.16 (59.26, 64.85) | 63.23 (58.28, 67.78) | 0.07 (-0.02, 0.15) | 0.116 |
| Western Sub-Saharan Africa | 37.38 (30.15, 54.05) | 38.46 (31.19, 51.7) | 0.10 (0.01, 0.2) | 0.033* |
| **YLDs** |  |  |  |  |
| Andean Latin America | 2.99 (1.98, 4.12) | 4.53 (2.91, 6.86) | 1.33 (1.00, 1.67) | <0.001* |
| Australasia | 4.63 (3.29, 6.23) | 5.66 (3.79, 8.03) | 0.72 (0.41, 1.03) | <0.001* |
| Caribbean | 5.31 (3.77, 7.03) | 8.46 (5.84, 11.32) | 1.71 (1.52, 1.90) | <0.001* |
| Central Asia | 5.02 (3.55, 6.69) | 5.78 (4.03, 7.74) | 0.38 (0.22, 0.53) | <0.001* |
| Central Europe | 6.63 (4.74, 8.80) | 10.48 (7.26, 14.21) | 1.56 (1.30, 1.82) | <0.001* |
| Central Latin America | 1.78 (1.29, 2.35) | 3.16 (2.17, 4.31) | 2.06 (1.81, 2.31) | <0.001* |
| Central Sub-Saharan Africa | 1.02 (0.65, 1.59) | 1.18 (0.73, 1.91) | 0.50 (0.22, 0.79) | 0.001* |
| East Asia | 2.32 (1.46, 3.32) | 3.43 (2.17, 5.15) | 1.36 (1.15, 1.57) | <0.001* |
| Eastern Europe | 10.19 (7.29, 13.42) | 14.27 (9.75, 20.18) | 1.22 (-0.09, 2.54) | 0.068 |
| Eastern Sub-Saharan Africa | 1.18 (0.72, 1.69) | 1.44 (0.92, 2.10) | 0.71 (0.40, 1.03) | <0.001* |
| High-income Asia Pacific | 3.33 (2.35, 4.45) | 5.98 (3.90, 8.59) | 2.00 (1.66, 2.35) | <0.001* |
| High-income North America | 9.83 (7.09, 12.95) | 14.34 (9.91, 19.69) | 1.31 (0.93, 1.69) | <0.001* |
| North Africa and Middle East | 1.34 (0.85, 1.91) | 2.66 (1.62, 3.7) | 2.37 (2.12, 2.63) | <0.001* |
| Oceania | 2.85 (1.76, 4.05) | 3.68 (1.88, 5.51) | 0.86 (0.80, 0.92) | <0.001* |
| South Asia | 0.80 (0.52, 1.14) | 1.28 (0.83, 1.84) | 1.70 (1.32, 2.08) | <0.001* |
| Southeast Asia | 2.07 (1.27, 2.88) | 2.98 (1.77, 4.23) | 1.28 (1.14, 1.43) | <0.001* |
| Southern Latin America | 3.16 (2.23, 4.24) | 4.02 (2.58, 5.75) | 0.85 (0.69, 1.02) | <0.001* |
| Southern Sub-Saharan Africa | 1.40 (0.94, 1.94) | 2.09 (1.32, 2.86) | 1.43 (0.95, 1.91) | <0.001* |
| Tropical Latin America | 2.49 (1.81, 3.28) | 3.32 (2.35, 4.42) | 1.07 (0.95, 1.18) | <0.001* |
| Western Europe | 6.68 (4.81, 8.92) | 10.22 (7.08, 13.73) | 1.45 (1.11, 1.80) | <0.001* |
| Western Sub-Saharan Africa | 0.79 (0.52, 1.18) | 1.05 (0.69, 1.56) | 0.99 (0.78, 1.20) | <0.001* |
| **YLLs** |  |  |  |  |
| Andean Latin America | 110.36 (88.77, 126.59) | 81.02 (63.97, 106.37) | -1.06 (-1.17, -0.95) | <0.001* |
| Australasia | 57.08 (53.81, 59.98) | 50.64 (45.88, 55.41) | -0.38 (-0.69, -0.07) | 0.015* |
| Caribbean | 114.21 (104.92, 125.43) | 138.52 (116.80, 164.37) | 0.72 (0.24, 1.19) | 0.003* |
| Central Asia | 107.55 (101.77, 113.82) | 83.94 (75.09, 93.90) | -0.87 (-1.15, -0.59) | <0.001* |
| Central Europe | 101.95 (98.49, 107.41) | 86.00 (74.12, 99.41) | -0.60 (-0.82, -0.37) | <0.001* |
| Central Latin America | 48.63 (46.78, 50.70) | 44.20 (37.40, 51.72) | -0.31 (-0.53, -0.10) | 0.005* |
| Central Sub-Saharan Africa | 52.38 (39.16, 72.64) | 47.40 (33.7, 67.83) | -0.39 (-0.44, -0.33) | <0.001* |
| East Asia | 66.46 (48.19, 82.49) | 32.27 (25.06, 43.38) | -2.45 (-2.59, -2.30) | <0.001* |
| Eastern Europe | 113.95 (109.55, 119.27) | 96.52 (82.90, 112.22) | -0.49 (-1.33, 0.36) | 0.255 |
| Eastern Sub-Saharan Africa | 60.38 (43.01, 74.97) | 55.20 (39.09, 68.38) | -0.3 (-0.39, -0.22) | <0.001* |
| High-income Asia Pacific | 46.63 (39.27, 49.11) | 36.40 (33.60, 38.66) | -0.85 (-1.02, -0.68) | <0.001* |
| High-income North America | 62.97 (60.68, 64.58) | 71.28 (68.20, 73.99) | 0.43 (0.32, 0.54) | <0.001* |
| North Africa and Middle East | 39.57 (30.36, 47.53) | 36.67 (26.36, 43.49) | -0.26 (-0.43, -0.10) | 0.002* |
| Oceania | 91.75 (63.05, 115.28) | 109.55 (60.82, 146.95) | 0.60 (0.54, 0.66) | <0.001* |
| South Asia | 36.10 (28.39, 45.72) | 34.95 (27.54, 44.41) | -0.07 (-0.60, 0.46) | 0.783 |
| Southeast Asia | 61.09 (43.60, 72.69) | 56.85 (37.52, 67.65) | -0.24 (-0.29, -0.19) | <0.001* |
| Southern Latin America | 69.65 (65.89, 73.31) | 52.66 (48.17, 57.34) | -0.98 (-1.10, -0.85) | <0.001* |
| Southern Sub-Saharan Africa | 47.99 (38.69, 56.26) | 60.54 (42.53, 70.67) | 0.76 (0.18, 1.35) | 0.010* |
| Tropical Latin America | 69.38 (66.14, 72.44) | 53.93 (50.44, 57.54) | -0.80 (-0.90, -0.70) | <0.001* |
| Western Europe | 55.48 (53.31, 56.92) | 53.02 (49.57, 55.63) | -0.16 (-0.27, -0.05) | 0.005* |
| Western Sub-Saharan Africa | 36.60 (29.49, 53.01) | 37.41 (30.25, 50.37) | 0.08 (-0.01, 0.17) | 0.091 |

ASR: age-standardized rates; AAPC: average annual percentage change; *: P<0.05.

Table S6：Prediction result by Norpred model

| **Age** | **Rate per 100,000 population** | | | | |
| --- | --- | --- | --- | --- | --- |
|  | **2020-2024** | **2025-2029** | **2030-2034** | **2035-2039** | **2040-2044** |
| **Incidence** |  |  |  |  |  |
| *Age-standardized* | 9.77 | 9.50 | 9.29 | 9.15 | 8.99 |
| 20 to 24 | 0.35 | 0.32 | 0.30 | 0.30 | 0.29 |
| 25 to 29 | 0.78 | 0.74 | 0.70 | 0.69 | 0.67 |
| 30 to 34 | 1.72 | 1.55 | 1.50 | 1.47 | 1.44 |
| 35 to 39 | 3.41 | 3.22 | 2.99 | 2.94 | 2.90 |
| 40 to 44 | 5.84 | 5.63 | 5.43 | 5.15 | 5.08 |
| 45 to 49 | 11.83 | 11.30 | 11.09 | 10.87 | 10.38 |
| 50 to 54 | 23.42 | 22.31 | 21.66 | 21.54 | 21.16 |
| 55 to 59 | 34.73 | 32.95 | 31.81 | 31.25 | 31.09 |
| 60 to 64 | 42.96 | 41.53 | 39.86 | 38.91 | 38.25 |
| 65 to 69 | 47.97 | 46.43 | 45.33 | 43.94 | 42.92 |
| 70 to 74 | 49.86 | 49.64 | 48.50 | 47.80 | 46.36 |
| 75 to 79 | 45.66 | 48.17 | 48.39 | 47.71 | 47.03 |
| 80 to 84 | 40.60 | 40.98 | 43.69 | 44.31 | 43.68 |
| 85 to 89 | 35.27 | 35.73 | 36.42 | 39.26 | 39.83 |
| 90 to 94 | 28.47 | 26.82 | 27.46 | 28.31 | 30.64 |
| 95 plus | 28.50 | 26.23 | 24.95 | 25.83 | 26.64 |
| **Deaths** |  |  |  |  |  |
| *Age-standardized* | 1.99 | 1.88 | 1.80 | 1.75 | 1.70 |
| 20 to 24 | 0.03 | 0.02 | 0.02 | 0.02 | 0.02 |
| 25 to 29 | 0.07 | 0.06 | 0.06 | 0.06 | 0.05 |
| 30 to 34 | 0.15 | 0.14 | 0.13 | 0.13 | 0.12 |
| 35 to 39 | 0.32 | 0.30 | 0.29 | 0.28 | 0.27 |
| 40 to 44 | 0.68 | 0.64 | 0.62 | 0.61 | 0.60 |
| 45 to 49 | 1.35 | 1.23 | 1.19 | 1.18 | 1.17 |
| 50 to 54 | 2.51 | 2.28 | 2.14 | 2.11 | 2.11 |
| 55 to 59 | 4.26 | 3.89 | 3.64 | 3.49 | 3.45 |
| 60 to 64 | 7.10 | 6.72 | 6.28 | 6.01 | 5.79 |
| 65 to 69 | 10.23 | 9.41 | 9.06 | 8.64 | 8.29 |
| 70 to 74 | 13.04 | 12.45 | 11.65 | 11.40 | 10.89 |
| 75 to 79 | 14.97 | 14.85 | 14.40 | 13.69 | 13.40 |
| 80 to 84 | 17.92 | 17.25 | 17.34 | 17.05 | 16.24 |
| 85 to 89 | 20.58 | 20.07 | 19.59 | 19.94 | 19.62 |
| 90 to 94 | 24.97 | 23.59 | 23.31 | 23.04 | 19.62 |
| 95 plus | 31.43 | 29.66 | 28.41 | 28.43 | 19.62 |

Figure S1


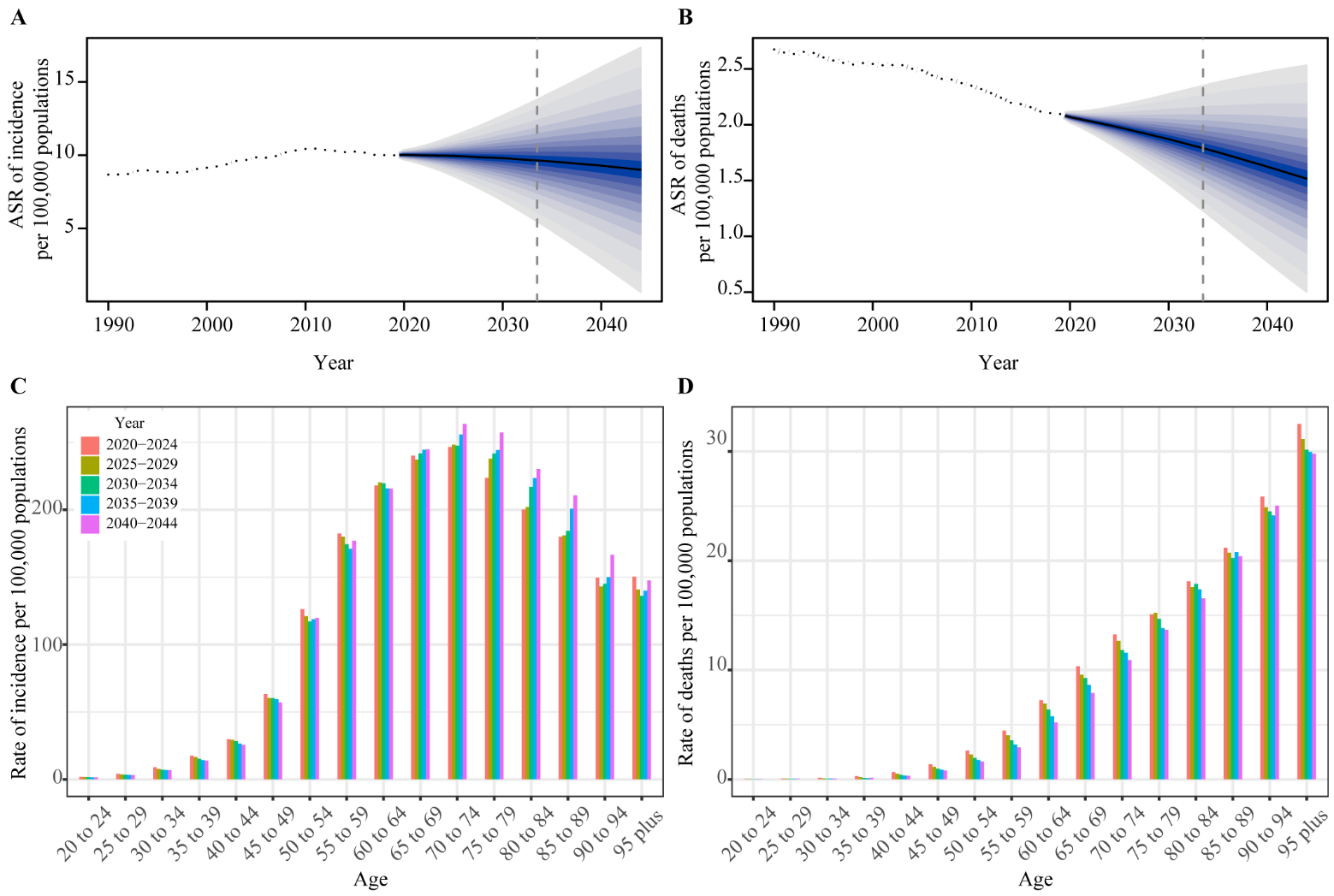


Table S7：Prediction result by BAPC model

| **Age** | **Rate per 100,000 population** | | | | |
| --- | --- | --- | --- | --- | --- |
|  | **2020-2024** | **2025-2029** | **2030-2034** | **2035-2039** | **2040-2044** |
| **Incidence** |  |  |  |  |  |
| *Age-standardized* | 10.00 | 9.89 | 9.71 | 9.46 | 9.15 |
| 20 to 24 | 1.76 | 1.60 | 1.49 | 1.43 | 1.42 |
| 25 to 29 | 4.11 | 3.62 | 3.37 | 3.23 | 3.20 |
| 30 to 34 | 8.96 | 7.85 | 7.16 | 6.86 | 6.77 |
| 35 to 39 | 17.58 | 16.63 | 15.18 | 14.26 | 13.85 |
| 40 to 44 | 29.87 | 29.29 | 28.39 | 26.62 | 25.54 |
| 45 to 49 | 63.18 | 60.28 | 60.19 | 59.53 | 57.02 |
| 50 to 54 | 126.24 | 121.07 | 116.99 | 118.76 | 119.75 |
| 55 to 59 | 182.34 | 180.06 | 174.46 | 171.10 | 177.02 |
| 60 to 64 | 218.05 | 220.26 | 219.60 | 215.83 | 215.72 |
| 65 to 69 | 240.12 | 237.13 | 241.78 | 244.48 | 244.83 |
| 70 to 74 | 246.57 | 248.22 | 247.41 | 255.84 | 263.59 |
| 75 to 79 | 223.57 | 237.83 | 241.63 | 244.25 | 257.37 |
| 80 to 84 | 200.13 | 202.02 | 216.90 | 223.47 | 230.19 |
| 85 to 89 | 179.98 | 180.89 | 184.31 | 200.72 | 210.72 |
| 90 to 94 | 149.44 | 143.07 | 145.11 | 149.99 | 166.48 |
| 95 plus | 150.30 | 140.75 | 136.03 | 139.99 | 147.49 |
| **Deaths** |  |  |  |  |  |
| *Age-standardized* | 2.03 | 1.93 | 1.82 | 1.70 | 1.57 |
| 20 to 24 | 0.15 | 0.13 | 0.11 | 0.09 | 0.08 |
| 25 to 29 | 0.36 | 0.29 | 0.25 | 0.22 | 0.20 |
| 30 to 34 | 0.73 | 0.43 | 0.33 | 0.36 | 0.37 |
| 35 to 39 | 1.54 | 0.97 | 0.63 | 0.62 | 0.70 |
| 40 to 44 | 3.34 | 2.56 | 2.02 | 1.75 | 1.67 |
| 45 to 49 | 6.97 | 5.73 | 4.90 | 4.38 | 4.01 |
| 50 to 54 | 13.16 | 11.35 | 9.84 | 8.88 | 8.19 |
| 55 to 59 | 22.22 | 20.09 | 17.82 | 15.88 | 14.61 |
| 60 to 64 | 36.19 | 34.67 | 31.91 | 28.81 | 26.07 |
| 65 to 69 | 51.70 | 47.83 | 46.34 | 43.19 | 39.47 |
| 70 to 74 | 66.28 | 63.37 | 59.16 | 57.93 | 54.58 |
| 75 to 79 | 75.46 | 76.19 | 73.42 | 69.19 | 68.46 |
| 80 to 84 | 90.59 | 87.88 | 89.39 | 86.91 | 82.76 |
| 85 to 89 | 105.9 | 103.68 | 101.27 | 103.9 | 102.04 |
| 90 to 94 | 129.27 | 124.40 | 122.58 | 120.74 | 125.12 |
| 95 plus | 162.56 | 155.71 | 150.75 | 149.76 | 148.98 |
